# Copper-mediated peptide arylation selective for the N-terminus†

**DOI:** 10.1039/d0sc02933e

**Published:** 2020-09-14

**Authors:** Mary K. Miller, Haopei Wang, Kengo Hanaya, Olivia Zhang, Alex Berlaga, Zachary T. Ball

**Affiliations:** Department of Chemistry, Rice University Houston TX 77005 USA zb1@rice.edu

## Abstract

Polypeptides present remarkable selectivity challenges for chemical methods. Amino groups are ubiquitous in polypeptide structure, yet few paradigms exist for reactivity and selectivity in arylation of amine groups. This communication describes the utilization of boronic acid reagents bearing certain *o*-electron withdrawing groups for copper-mediated amine arylation of the N-terminus under mild conditions and primarily aqueous solvent. The method adds to the toolkit of boronic acid reagents for polypeptide modification under mild conditions in water that shows complete selectivity for the N-terminus in the presence of lysine side chains.

## Abstract 2

The discovery of unique Chan-Lam coupling reactivity of arylboronic acids containing an *ortho*-sulfonamide group allows site-specific tailoring of peptide structure.

## Graphical abstract

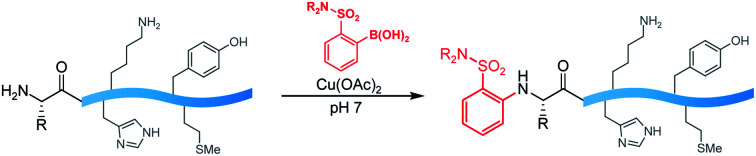

## Introduction

Methods for selective modification of polypeptides and proteins allow access to drug conjugates, imaging probes, and hybrid materials.^1,2^ At the same time, polypeptides represent a stringent test of chemists' ability to control reactivity and chemoselectivity in a diverse, polyfunctional environment. The amino (–NH_2_) group is a common bioconjugation site, found primarily at the N-terminus and at lysine side chains.

Cross-coupling reactivity, typically mediated by transition metal complexes, have become important tools for N–C bond formation, and they represent an interesting approach to selective modification of peptides and proteins.^3–7^ Chemoselective cysteine coupling has been achieved with a variety of transition metal-mediated approaches,^8–11^ and tyrosine is selectively modified with π-allyl intermediates.^12^ In contrast, cross-coupling at amine groups in polypeptides are limited to a few special cases, including arylpalladium reagents that mediate arylation of lysine side chains in non-aqueous solvents^13^ and arylation of individual amino acid derivatives with Cu^14–16^ or Pd,^17,18^ typically at elevated temperatures under non-aqueous conditions. Perhaps surprisingly, cross-coupling at amino groups (N-terminus and/or lysine) under physiologically relevant aqueous conditions remains an unsolved problem, and selectivity questions for the common case of multiple amino groups are largely unaddressed. While copper is well known to bind N-terminal motifs,^19–21^ these coordination complexes have not been utilized as intermediates in productive bond-forming catalysis, and indeed binding to the amino terminus is typically employed as a protecting group to block reactivity. Herein, we describe arylation with remarkable selectivity for the amino terminus, without competing reactivity at lysine. Simple copper(ii) salts mediate the reaction with arylboronic acid reagents under mild conditions in aqueous buffer.

**Fig. 1:**
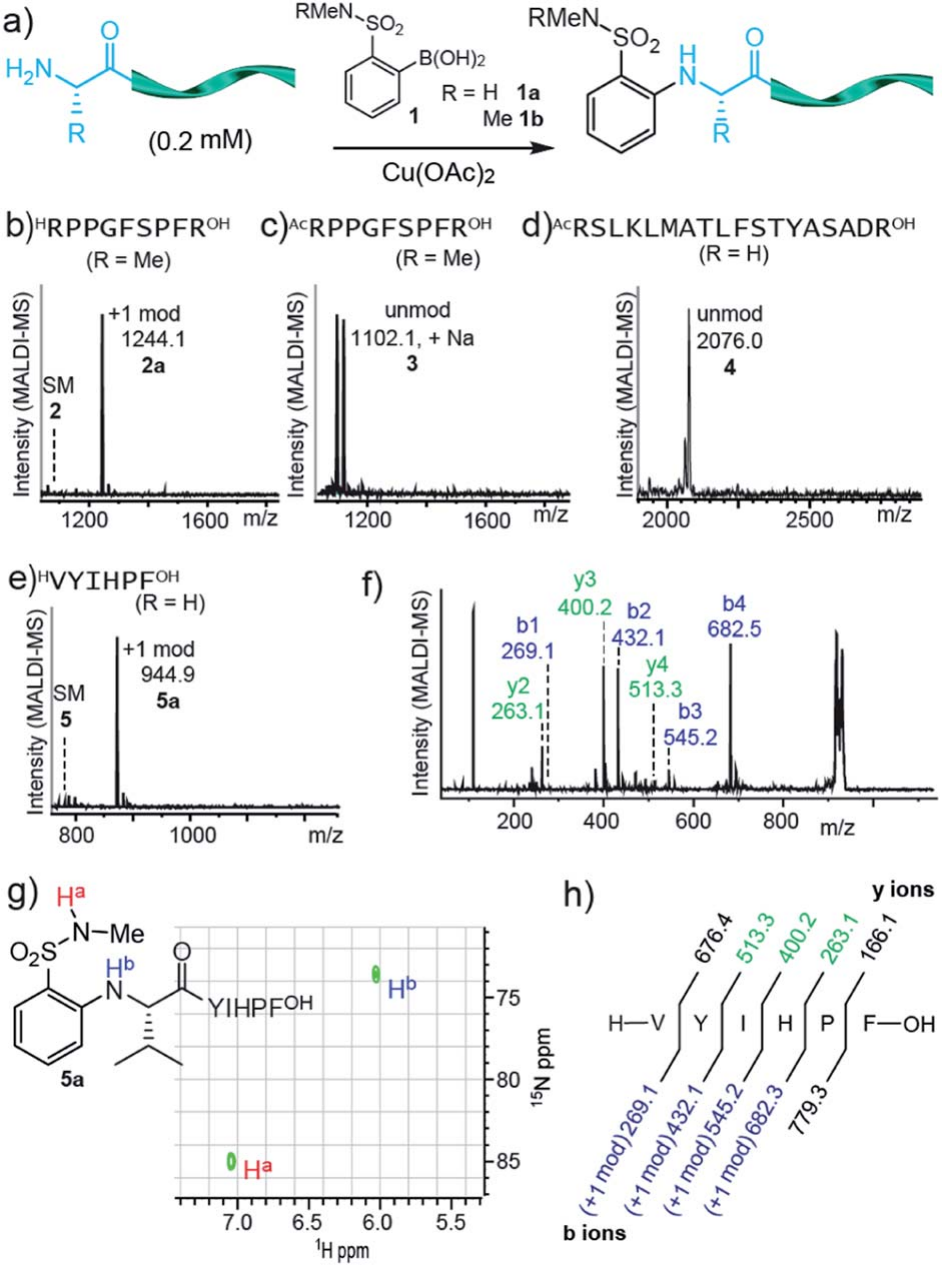
(a) Modification of peptides **2–5** with boronic acids **1a** and **1b**. Conditions: peptide (0.2 mM), boronic acid **1a/b** (2 mM) and Cu(OAc)_2_ (0.1 mM) in NMM buffer (10 mM, pH 9.0) with 30% TFE at 37 °C for 18 h. For **5**, HEPES buffer (10 mM, pH = 7.0) with 20% acetonitrile was employed. (b–e) MALDI-MS spectrum (crude) for reaction of peptide **2–5**. (f) MS/MS spectrum of product **5a**. (g) ^1^H–^15^N HSQC NMR spectrum of product **5a**. (h) Sequence and fragmentation ladder of product **5a**. Observed b and y ions are indicated.

During investigations into boronic acid couplings with backbone amide N–H bonds,^3,4,22^ we observed a switch in chemoselectivity with certain *ortho*-substituted arylboronic acids. With sulfonamide-substituted reagent **1**, we observed no reactivity at amide N–H bonds, but instead observed highly chemoselective arylation of the N-terminal amino group (Fig. 1a and b). We decided to investigate this unique selectivity, mindful that N-terminal cross-coupling could serve as a useful complement to alternative N-terminal modification methods—such as aldehyde condensation,^23–29^ pH-controlled acylation,^30–33^ N-terminal oxidation,^34^ and diazo transfer^33^—and to other emerging concepts for amine-selective bioconjugation.^35^

## Results and discussion

Remarkably, primary amine groups of lysine side chain were completely unreactive (Fig. 1d), and an N-terminally acetylated analog of a reactive sequence resulted in no modification, consistent with reaction at an amino group. MALDI-MS/MS (Fig. S27 and S28†) verified N-terminal reactivity in these cases. The structure of modified peptide **5a** was confirmed by MALDI-MS/MS (Fig. 1f and h) and ^15^N HSQC experiments (Fig. 1g), and MALDI-MS/MS also established N-terminal selectivity for peptides with multiple amino groups.

Encouraged by these observations, we set out to understand the scope of the reaction conditions using a model peptide, **5** (Table 1). Yields were determined by HPLC analysis with an internal standard (see ESI and Fig. S3† for details). For reactions with peptide **5**, pH = 7.0 was best; increasing or decreasing pH resulted in decreased yields (entry 1, 3–4). Pleasantly, the reaction was scalable, and the arylated peptide (entry 2) was isolated in 68% yield, similar to that observed by HPLC on small scale under identical conditions.

**Table 1:** Scope of the reaction conditions

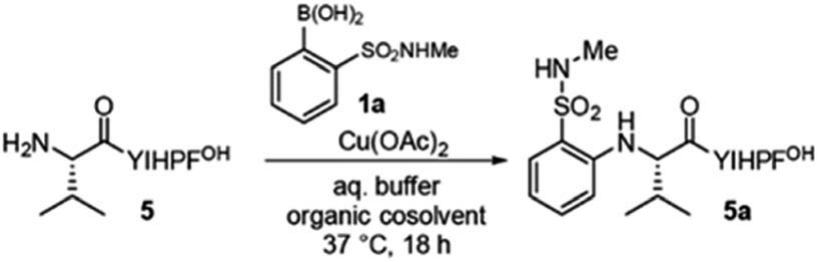				
Entry	pH	Buffer	Cosolvent	Yield^a^ (%)
1	6.0	NMM	30% TFE	15
2	7.0	NMM	30% TFE	66^b^ (68)^c^
3	8.0	NMM	30% TFE	33
4	9.0	NMM	30% TFE	18
5	7.0	NMM	None	53
6	7.0	NMM	30% DMSO	70
7	7.0	NMM	30% MeCN	87
8	7.0	NMM	40% MeCN	44
9	7.0	Tris	20% MeCN	11
10	7.0	HEPES	20% MeCN	97

a Yield calculated by RP-HPLC.

b 5 mg scale reaction.

c Isolated yield.

While the reaction can be performed under strictly aqueous conditions (entry 5), the addition of an organic cosolvent improved yields (entry 7). The nature of the buffer impacts reaction efficiency. The yield increased to near-quantitative levels (97%) for reactions in HEPES buffer (entry 10), while Tris, which contains a primary amine, was a poor buffer choice (entry 9).

**Fig. 2:**
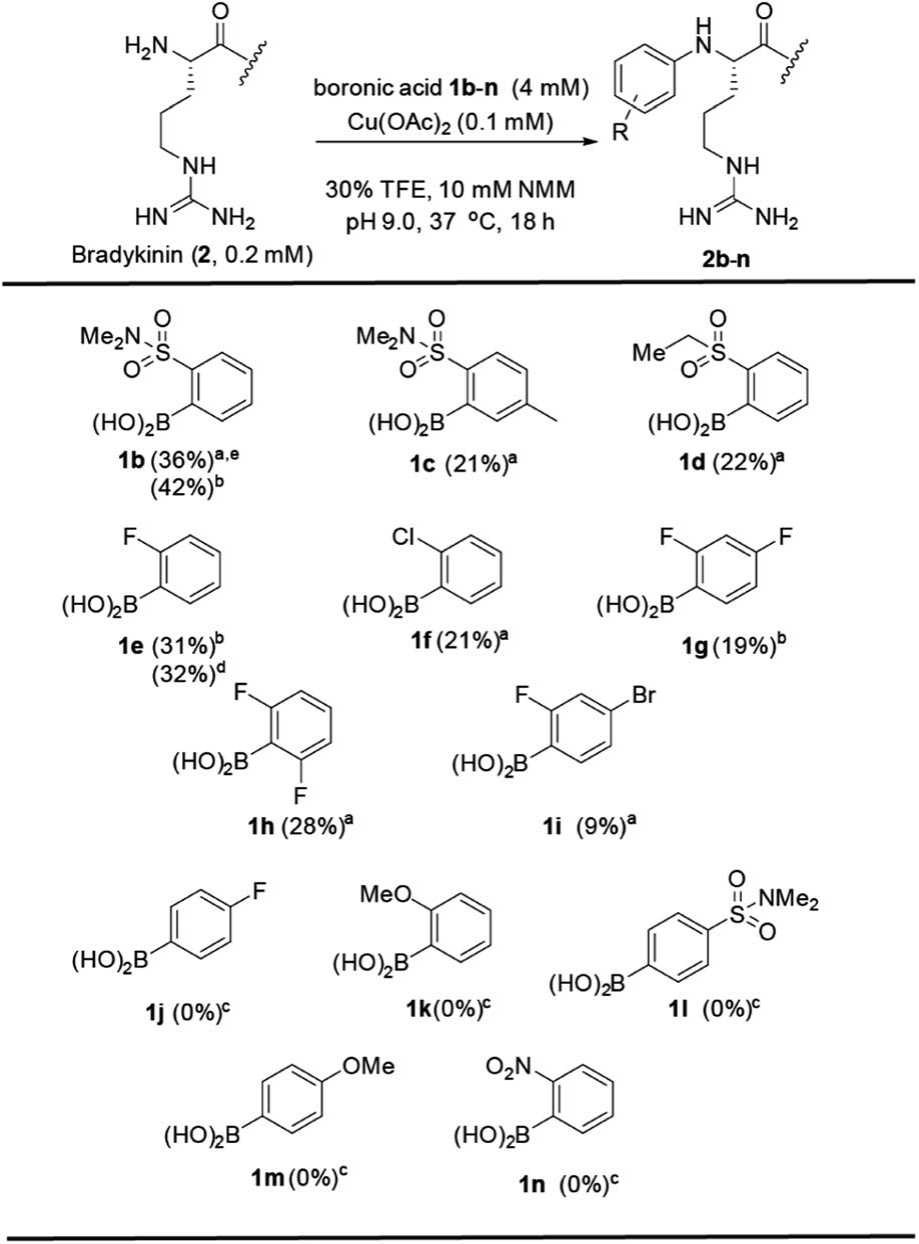
Scope of the boronic acid reagents. Conditions: Bradykinin (**2**) (0.2 mM), boronic acid **1b–n** (4 mM), and Cu(OAc)_2_ (0.1 mM) in NMM buffer (10 mM, pH = 9.0) at 37 °C for 18 h. ^a^RP-HPLC yield determined using internal standards. ^b^Isolated yield on a 10 mg scale. ^c^Yield determined by ESI-MS. ^d^Yield determined using peak area of a known concentration of isolated product. ^e^Boronic acid was added as seven aliquots over 25 h.

Examining the scope of boronic acids revealed a structure–reactivity relationship (Fig. 2). Arylation products with peptide **2** were observed only with select electron-withdrawing *ortho* substituents: sulfonamide group (**1b–c**), sulfone group (**1d**) and halogen groups (**1e–i**). This trend is quite different from that observed with metal-catalyzed reactions of boronic acids with backbone amide^3^ or cysteine^8^ side chains. Additional substitution at distal positions (**1c**, **1g**, **1i**) is tolerated. The successful coupling with **1i**, for example, introduces an arylbromide handle for later elaboration. The reaction has a strict requirement for *ortho* substitution (**1l**). Surprisingly, no product was observed with an *ortho*-nitro group, despite the excellent reactivity of this compound in both cysteine^8^ and amide N–H^5^ arylation. These observations implicate a substantially different reactivity type in the present N-terminal reactivity.^36,37^

To determine the tolerance of the reaction for different N-terminal residues, we synthesized variants at the N-terminal residue (**5–13**, Table 2). The reaction tolerates a wide variety of N-terminal residues (entries 1–5, 7–11), including bulky residues (tryptophan, valine, and leucine), charged residues (arginine, aspartate) and glycine. Proline, which contains a secondary amine (entry 6), was not tolerated. In all cases, only a single product is observed. HPLC analysis shows no evidence of coupling at sites other than the N-terminus, or of any side products, even for peptides with potentially reactive N-terminal side chains (*i.e.***4**, **8**) (see Fig. S6–S13†). The modest yields observed in a few cases (*i.e.***8**, **12**) are the result of incomplete conversion or, more commonly, partial starting material decomposition into unknown species.

**Table 2:** Scope N-terminal residues

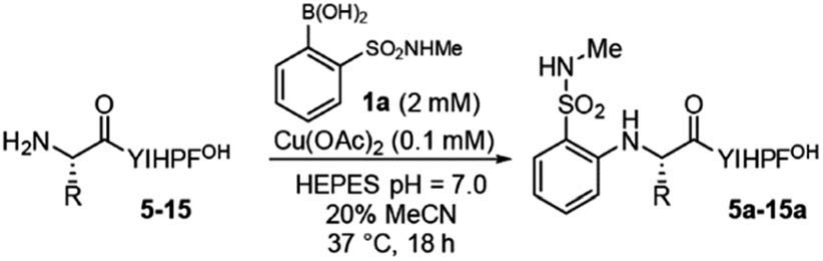			
Entry	R_1_	Yield^a^ (%)	
1	Val (**5**)	97	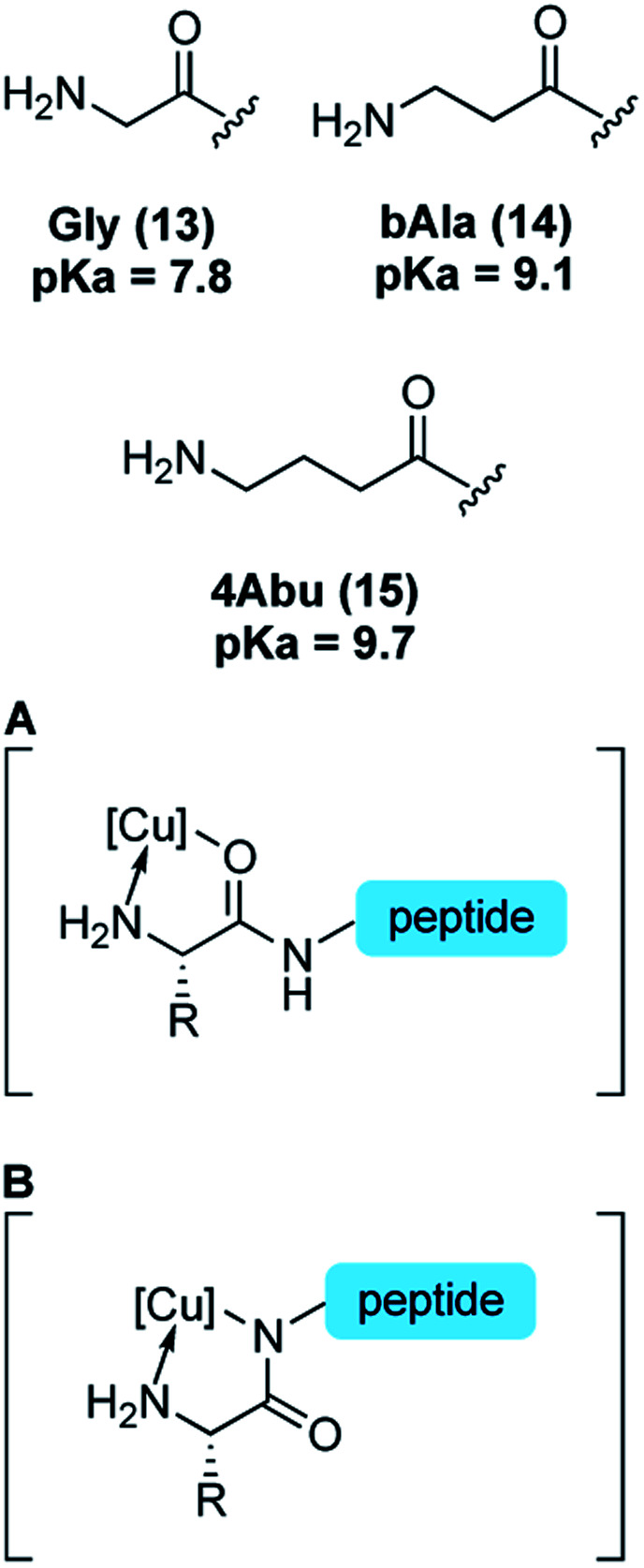
2	Leu (**6**)	80^b^	
3	Phe (**7**)	50	
4	Trp (**8**)	44	
5	Arg (**9**)	96	
6	Pro (**10**)	<5	
7	Ser (**11**)	93	
8	Asp (**12**)	64	
9	Gly (**13**)	96	
10	bAla (**14**)	64	
11	4Abu (**15**)	<5	

a Yield calculated by RP-HPLC

b 10 mg scale

Reactivity at N-terminal amino groups in the presence of lysine side-chain amines is an interesting chemoselectivity. We hypothesized that the origin of chemoselectivity could be either p*K*_a_ differences^38^ or the intermediacy of a chelation complex (Table 2, at right) of the N-terminal amine, not possible at lysine side chains. To probe this, we examined peptides with unnatural amino acids that would require larger ring-chelate structures: β-alanine (bAla) (entry 10) and γ-aminobutyric acid (4Abu) (entry 11). Relative to glycine, these peptide variants have a higher p*K*_a_ (Table 2).^39^ We found that the bAla peptide, capable of forming a 6-membered ring chelate, retained reactivity, while the 4Abu variant—which would require at least a 7-membered ring intermediate—was unreactive. The chelation-driven selectivity model seems most in accord with these results, although additional study is warranted. In this context, it is worth noting that the reaction does not exhibit the hallmark increasing reactivity with increasing pH that is typically observed in amine functionalization governed by p*K*_a_.

Reactivity studies also shed light on the nature of a putative *κ*^2^ copper binding. While copper binding to N-terminal sequences is well studied,^19–21^ canonical structures typically adopt N-bound amidate structures (Table 2, **B**). In N-terminal arylation reported here, peptides with proline as the second amino acid, which have no amide N–H and thus cannot adopt ATCUN-like amidate structures, nonetheless are competent reaction partners (Table 3, entries 1–2). This observation would seem to indicate that neutral, O-bound proximal amide groups (Table 2, **A**) are competent species in catalysis.

**Table 3:** Additional peptide substrate examples

Entry	Peptide	Yield^a^ (%)
1	H–RPKPQQWFWLL–NH_2_ (**16**)	45
2	H–RPPGFSPFR–OH (**2**)	36
3	H–DRVYIHPFHL–OH (**17**)	20
4	H–MEVGWYRSPFSRVVHLYRNGK–OH (**18**)	18

a Yield calculated by RP-HPLC

We next examined reactivity of other peptide sequences (Table 3). A number of naturally occurring peptide sequences were amenable to this reaction. A 21-mer peptide indicates that the reaction tolerates quite lengthy sequences (entry 4), hinting at potential use in more demanding bioconjugation challenges.

**Fig. 3:**
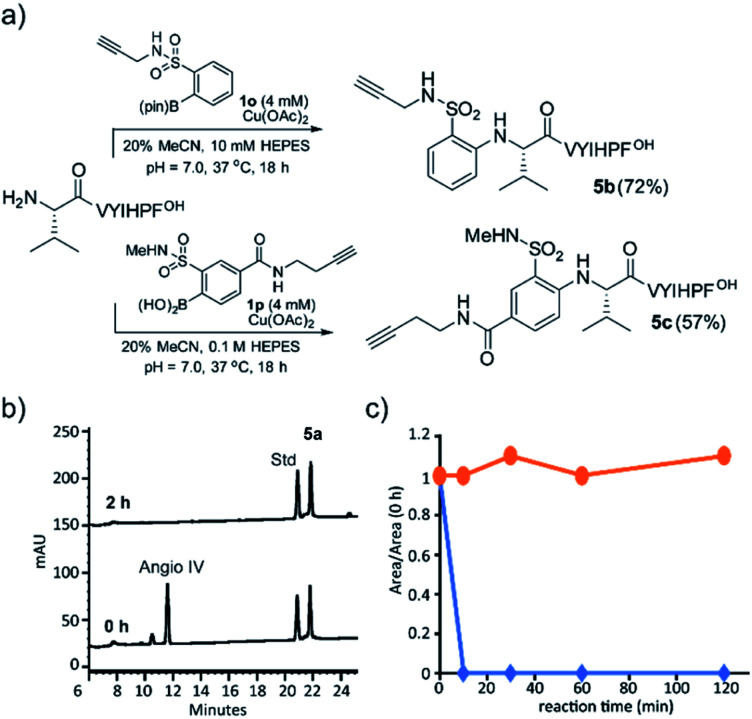
(a) Modification of peptide **5** with boronate ester **1o** and **1p**. Yield calculated by RP-HPLC. (b) RP-HPLC analysis of the enzymatic degradation of angiotensin IV (**5**) and arylated angiotensin IV (**5a**) with aminopeptidase I. Conditions: 100 μM **5**, 40 μM **5a**, 0.08 μg mL^−1^ aminopeptidase I, and 20 μM Co(OAc)_2_ in NaOAc buffer (5 mM, pH = 6.0) at 95 °C. Internal standard **19** for quantification. (c) Peptidase activity: time course for aminopeptidease I cleavage of arylated angiotensin IV (**5a**, orange) and angiotensin IV (**5**, blue).

The alkyne group is one of the most useful and general handles for manipulation of biomolecules, and we were gratified to find that boronic acids containing an alkyne handle retained efficient reactivity. A sulfonamide-linked alkyne boronic acid was an efficient reagent for N-terminal arylation (Fig. 3a).

Arylation significantly alters the N-terminal charge state, since the product aniline is expected to be uncharged under physiological conditions. We decided to investigate whether the charge and structural perturbation afforded by the *N*-arylation engendered peptide stability towards enzymatic degradation. Using aminopeptidase I, an enzyme that liberates the N-terminal residue from peptides and proteins, we followed the reaction of both angiotensin IV **5** and its arylated analog **5a** with Pfu aminopeptidase I and found that after 10 minutes **5** was completely consumed (Fig. 3b) while the **5a** remained stable even after 2 h incubation (Fig. 3c).

## Conclusion

In conclusion, copper(ii) salts together with boronic acids bearing *ortho*-sulfonamide groups induce N–H arylation that is specific for the N-terminus. The reaction proceeds under neutral conditions in water and allows arylation of a wide variety of N-terminal residues. The reactivity is indicative of a new selectivity paradigm for copper-catalyzed amine functionalization that relies on local structure.

## Conflicts of interest

There are no conflicts to declare.

## Supplementary Material

SC-011-D0SC02933E-s001
