# Supplementary material for: Copper-mediated peptide arylation selective for the N-terminus
Source: Chem Sci. 2020 Sep 14;11(38):10501–5. doi: 10.1039/d0sc02933e (PMC8162437; doi:10.1039/d0sc02933e)
Supplement: SC-011-D0SC02933E-s001 [file SC-011-D0SC02933E-s001.pdf]

Electronic Supplementary Material (ESI) for Chemical Science.
This journal is © The Royal Society of Chemistry 2020

Supporting Information

Copper-mediated peptide arylation selective for the N-terminus

Mary K. Miller‡, Haopei Wang‡, Kengo Hanaya, Olivia Zhang, Alex W. Berlaga, and Zachary T. Ball*

Department of Chemistry, Rice University, Houston, TX, 77005, United States

Table of Contents

General information and Methods 2

HPLC Quantification with Internal Standards 5

General Experimental Procedures 6

Peptide Synthesis and Arylation 6

Preparation of Reagents 8

Supplementary Data 14

For Figure 1 14

For Figure 2 22

For Figure 3 29

For Table 1 31

For Table 2 38

For Table 3 40

UV-visible Absorption Spectroscopy 43

Characterization- HPLC, ESI-MS, ^{1}H and ^{13}C NMR 46

HPLC and ESI-MS of Synthesized peptides from Table 2 46

ESI-MS of Synthesized Compounds 52

^{1}H and ^{13}C NMR spectra of synthesized compounds 56

General information and Methods

Chemicals

All chemicals used were purchased from commercial suppliers and used without further purification. Bradykinin (**2**) (H-RPPGFSPFR-OH) was purchased from Chem-Impex Int'l. Inc. (cat. #07913). Myosin H chain fragment, mouse (**4**) (Ac-RSLKLMATLFSTYASADR-OH) was purchased from Anaspec (AS-62554). Angiotensin IV (**5**) (H-VYIHPF-OH) was purchased from VWR (cat. #89143-064). Spantide I (RPKPQQFFGLM-NH_2) (**16**) was purchased from Fisher (cat. #50-259-551). Angiotensin I (**17**) (H-DRVYIHPFHL-OH) (A9650) from Sigma. MOG peptide 35-55 (**18**) (H-MEVGWYRSPFSRVVHLYRNGK-OH) was purchased from Anaspec (AS-60130-1). IL-8 inhibitor (**S19**) was purchased from Anaspec (AS-62401). The *N*-methylmorpholine (NMM) buffer (pH 7.0–8.0) was prepared by addition of NMM (TCI, M0370) to MilliQ water. The pH of the NMM solution was adjusted to the desired pH by addition of aq HCl. The 4-(2-hydroxyethyl)-1-piperazineethanesulfonic acid (HEPES) buffer (pH 6.0–9.0) was prepared by dissolution of the compound (Oakwood Chemicals, 047861) in MilliQ water, and the pH was adjusted by addition of aq NaOH or HCl. It was discovered that freshly prepared buffer can be important for optimal reactivity.

Instrumentation

Reverse-phase HPLC (RP-HPLC) was performed on a Shimadzu CBM-20a instrument with Phenomenex Jupiter 4µ Proteo 90A (250 mm×4.6 mm for analytical scale) and Phenomenex Jupiter 4µ Proteo 90A (250×15 mm for preparative scale) column. The flow rate was 1 mL/min for analytical scale and 8 mL/min for preparative scale. A gradient of acetonitrile/water with 0.1% trifluoroacetic acid (TFA) was employed. Compounds were detected by UV detector at 220 nm or 310 nm.

General Procedure for RP-HPLC Analysis: To the crude peptide reactions was added an internal standard of known concentration. The crude reaction mixture with added internal standard was analyzed using analytical RP-HPLC at 310 nm (unless otherwise specified).

ESI-MS was conducted on a Bruker Daltonics MicroTOF spectrometer.

MALDI-TOF MS was conducted on Bruker Daltonics Autoflex Speed-MALDI-TOF/TOF spectrometer for protein experiments. A sample was mixed with sinapic acid (20 mg/mL solution in 50:50:0.1 H_2O/MeCN/TFA).

^{1}H and ^{13}C NMR spectra were obtained on Bruker AVANCE 500 or AVANCE 600 spectrometer.

UV-Vis spectra were obtained on a Cary spectrophotometer.

Enzymatic Degradation with Aminopeptidase I

Stability Test of Peptides to Aminopeptidase I

To a solution of angiotensin IV **5** (25 µL, 2.0 mM in H_2O) and arylated angiotensin IV **5a** (11.9 µL, 1.7 mM in 20% aq. MeCN) and $Co(OAc)_2$ (50 µL, 200 µM in H_2O) in NaOAc buffer (338 µL, 5 mM, pH = 6.0) was added aminopeptidase I (75 µL, 0.5 mg/mL in Tris buffer (50 mM, pH = 6.0). A 50-µL aliquot was immediately removed after addition of the enzyme and was quenched with EDTA (5 µL, 1 M in H_2O). Internal standard 2-amino-*N,N*'-dimethylbenzenesulfonamide **19** was added (1.5 µL, 4 mM in DMSO) and the 0 h timepoint was analyzed by RP-HPLC (20-60% MeCN over 28 min) at 220 nm and 310 nm. The reaction mixture was then heated to 95 °C and 50-µL aliquots were removed and quenched with EDTA (5 µL, 1 M in H_2O) at 10 min, 30 min, 1 h, and 2 h. After quenching, the internal standard **19** was added (1.5 µL, 4 mM in DMSO) and analyzed by RP-HPLC (20-60% MeCN over 28 min) at 220 nm and 310 nm. The area of the starting material peaks was compared to the corresponding area at 0 h.

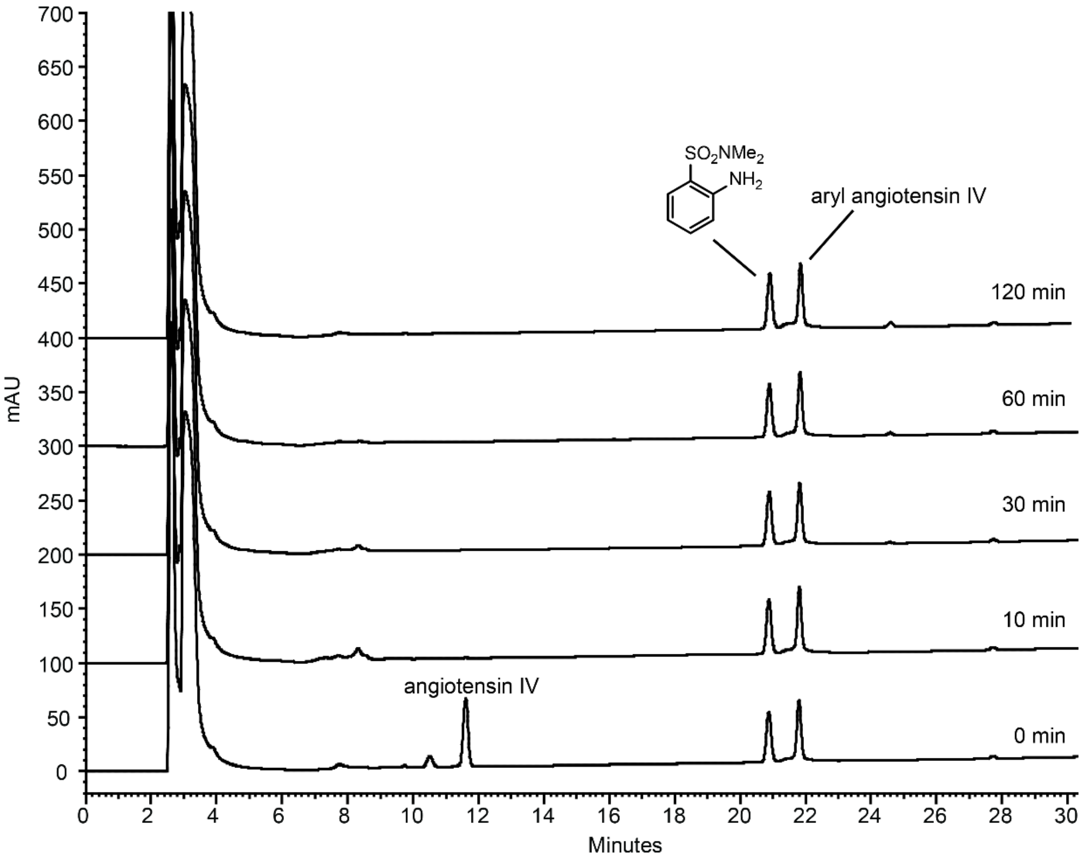

Figure S1. RP-HPLC trace of the reaction of angiotensin IV (**5**) and arylated angiotensin IV (**5a**) at 220 nm at various time points. Ramp: 20-60% MeCN in 28 mins.

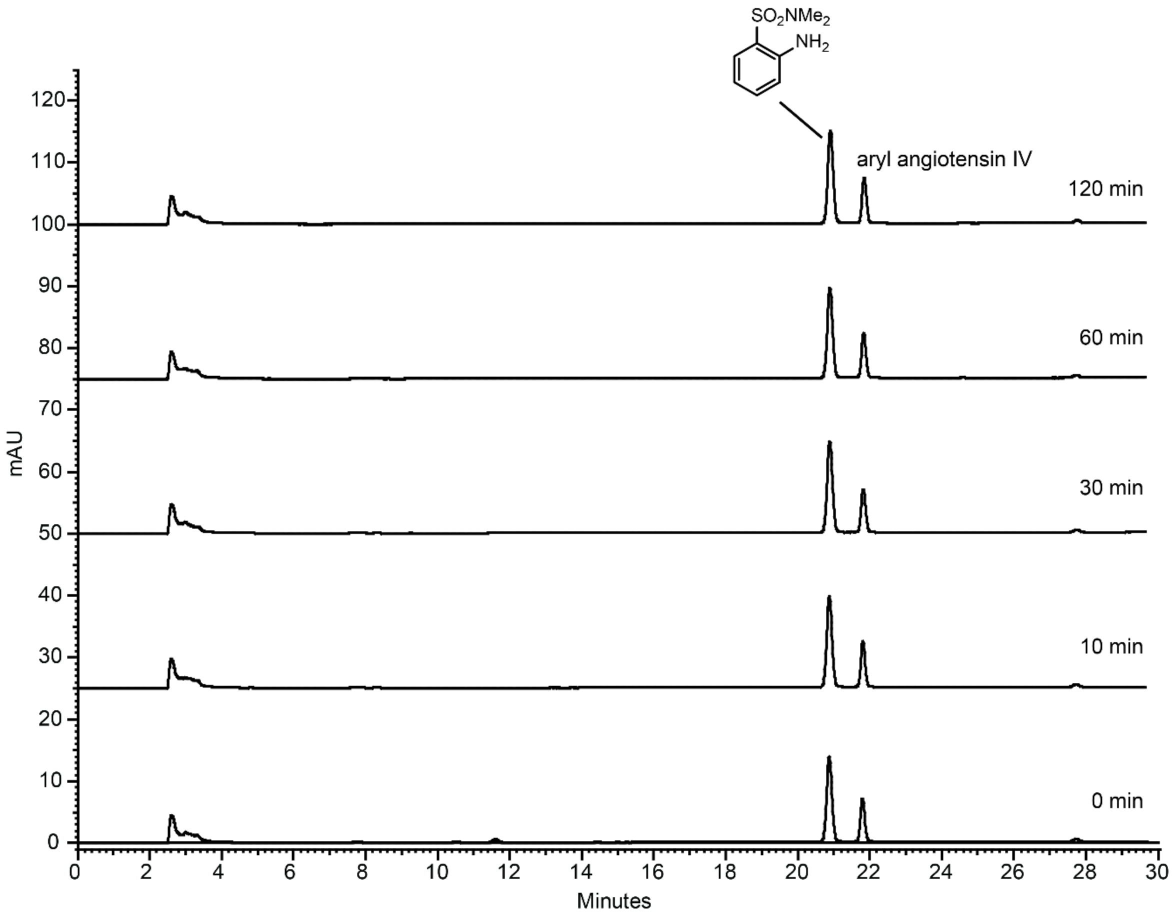

Figure S2. RP-HPLC trace of the reaction of angiotensin IV (**5**) and arylated angiotensin IV (**5a**) at 310 nm at various time points. Ramp: 20-60% MeCN in 28 mins.

HPLC Quantification with Internal Standards

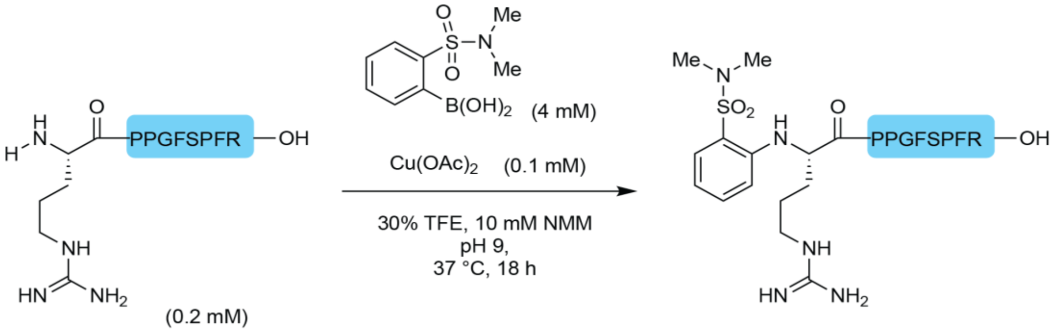

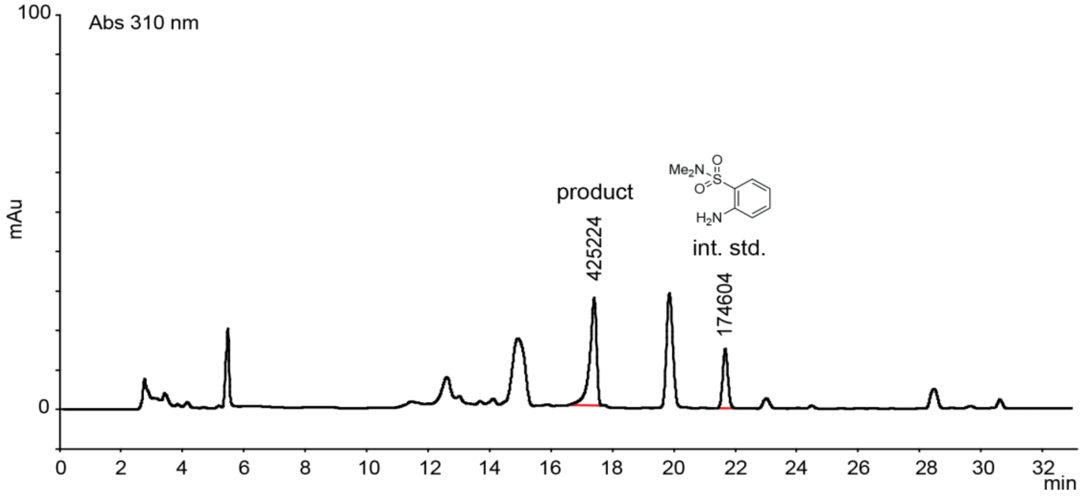

$$\%\ \text{yield} = \frac{\left(\frac{\text{Area}_{\text{prod}}}{\text{Area}_{\text{int. std.}}}\right)\times[\text{int. std.}]}{\text{yield}_{\text{theor.}}} \times 100$$

Area of product = 425224

Area of internal standard = 174604

Concentration of internal standard = 29.6 μM

Expected concentration of product = 200 μM

$$\%yield = \frac{\left(\frac{425224}{174604}\right)\times 29.6\ \mu M}{200\ \mu M} \times 100\% = 36\%$$

Figure S3. Sample calculation of yield calculated by analytical RP-HPLC.

General Experimental Procedures

Peptide Synthesis and Arylation

Peptide Synthesis:
Peptides from Table 2 were synthesized using standard solid phase peptide synthesis protocols[1] using rink amide resin (P3Biosystems, 52001) and Fmoc-amino acids purchased from NovaBiochem and used without further purification. Peptides were purified by reverse-phase HPLC and characterized by ESI-MS.

General procedure for N-terminal arylation

Arylation of Angiotensin IV (5) with boronic acid 1a: Preparation of 5a
Analytical scale: Angiotensin IV (**5**) (16 μL, 2.5 mM in H_2O) was incubated with (2-(*N*-methylsulfamoyl)phenyl)boronic acid **1a** (8 μL, 50 mM in DMSO) and $Cu(OAc)_2$ (8 μL, 2.5 mM in H_2O) in HEPES buffer (10 mM, pH = 7.0, 128 μL) with 20% acetonitrile (40 μL) at 37 °C for 18 h. The reactions were quenched with 1 μL 0.2 M EDTA. To the reaction mixture was added 2-amino-*N,N*'-dimethylbenzenesulfonamide **19** as an internal standard (1.5 μL, 4 mM in DMSO) and the yield was determined by RP-HPLC analysis (20-60% MeCN over 28 min) at 310 nm.

Preparative scale: Angiotensin IV (**5**) (4.9 mg, 0.063 mmol) was dissolved in NMM buffer (10 mM, pH = 7.0, 20.9 mL) and 2,2,2-trifluoroethanol (9.5 mL). To this was added (2-(*N*-methylsulfamoyl)phenyl)boronic acid (**1a**) (14.6 mg, 0.0679 mmol) as a solution in 1.25 mL DMSO. $Cu(OAc)_2$ (15.8 μL of a 200 mM stock in MQ H_2O) was finally added, and the reaction mixture was incubated at 37 °C for 18 h. The reaction mixture was quenched with EDTA (9.5 μL of a 1-M solution in H_2O) and a 200 μL aliquot was removed for analytical HPLC analysis, after which the remaining solution was concentrated by a gentle flow of nitrogen to approximately 1 mL. The product was purified by RP-HPLC for preparatory scale (20-60% MeCN over 40 min) to afford the arylated angiotensin IV **5a·TFA** (4.7 mg, 68%).

Arylation of Bradykinin (2) with boronic acid 1b: Preparation of 2a
Preparative Scale: Bradykinin, acetate salt (**2**) (10.6 mg, 0.0095 mmol) and $Cu(OAc)_2$ (0.9 mg, 0.0050 mmol) was dissolved in NMM buffer (10 mM, pH = 9.0, 35 mL). To this was added (2-(N,N-methylsulfamoyl)phenyl)boronic acid (**1b**) (43.3 mg, 0.19 mmol) and 2 mL DMSO. Then, 2,2,2-trifluoroethanol (15 mL) was added, and the reaction mixture was incubated at 37 °C for 18 h. Ethylenediaminetetraacetic acid tetrasodium salt (Na_4-EDTA, dihydrate) (16 mg, 0.038 mmol) was added, and the mixture was stirred at rt for 10 min. The reaction mixture was concentrated by a gentle flow of nitrogen to ~1 mL. The product was purified by RP-HPLC for preparatory scale (20-60% MeCN over 28 min) to afford the arylated bradykinin **2a** as the bis-TFA salt (5.8 mg, 42%).

Optimization of arylation conditions (Table 1)
The "General procedure for N-terminal arylation" was followed, with specific reactions conditions altered as indicated in the Table. The yield was determined by RP-HPLC analysis (20-60% MeCN over 28 min) at 310 nm as described.

Peptide Library Reaction Screening (Table 2)
A mixture of angiotensin IV-derived peptide **5-15** (16 μL, 2.5 mM in MQ H_2O) was incubated with (2-(*N*-methylsulfamoyl)phenyl)boronic acid **1a** (8 μL, 50 mM in DMSO) and $Cu(OAc)_2$ (8 μL, 2.5 mM in MQ H_2O) in HEPES buffer (10 mM, pH = 7.0, 128 μL) with 20% acetonitrile (40 μL) at 37 °C for 18 h. The reactions were quenched with 1 μL 0.2 M EDTA. To the reaction mixture was added 2-amino-*N,N*'-dimethylbenzenesulfonamide **19** as an internal standard (1.5 μL, 4 mM in DMSO) and the yield was determined by RP-HPLC analysis (20-60% MeCN over 28 min) at 310 nm.

Arylation of additional peptides (Table 3)

Arylation of Substance P (16) with boronic acid 1a: Preparation of 16a
A mixture of peptide **16** (8 μL, 2.5 mM in H_2O) was incubated with boronic acid **1a** (4 μL, 50 mM in DMSO) and $Cu(OAc)_2$ (4 μL, 2.5 mM in H_2O) in NMM buffer (54 μL, 10 mM, pH = 7.0) with 30% DMSO (30 μL) at 37 °C for 18 h. The reactions were quenched with 1 μL 0.1 M EDTA. To the crude reaction mixture was added 2-amino-*N,N*'-dimethylbenzenesulfonamide **19** was added as an internal standard (1.5 μL, 4 mM in DMSO), and the yield was determined by RP-HPLC analysis (20-60% MeCN over 28 min) at 310 nm.

Arylation of Angiotensin I (17) with boronic acid 1a: Preparation of 17a
A mixture of peptide **17** (8 μL, 2.5 mM in H_2O) was incubated with boronic acid **1a** (4 μL, 50 mM in DMSO) and $Cu(OAc)_2$ (4 μL, 2.5 mM in MQ H_2O) in HEPES buffer (64 μL, 10 mM, pH = 7.0) with 20% DMSO (20 μL) at 37 °C for 18 h. The reactions were quenched with 1 μL 0.1 M EDTA. To the reaction micxture was added 2-amino-*N,N*'-dimethylbenzenesulfonamide **19** as an internal standard (1.5 μL, 4 mM in DMSO) and the yield was determined by RP-HPLC analysis (20-60% MeCN over 28 min) at 310 nm.

Arylation of MOG 35-55 (18) with boronic acid 1a: Preparation of 18a
A mixture of peptide **18** (8 μL, 2.5 mM in H_2O) was incubated with boronic acid **1a** (4 μL, 50 mM in DMSO) and $Cu(OAc)_2$ (4 μL, 2.5 mM in H_2O) in HEPES buffer (10 mM, pH = 7.0, 54 μL) with 30% MeCN (30 μL) at 37 °C for 18 h. The reactions were quenched with 1 μL 0.1 M EDTA. To the reaction mixture was added 2-amino-*N,N*'-dimethylbenzenesulfonamide **19** as an internal standard (1.5 μL, 4 mM in DMSO) and the yield was determined by RP-HPLC analysis (20-60% MeCN over 28 min) at 310 nm.

Investigation of boronic acid scope (Figure 2)
A mixture of bradykinin **2** (9.6 μL, 2.5 mM in H_2O) was incubated with boronic acids **1b-1n**. (9.6 μL, 50 mM in DMSO) and $Cu(OAc)_2$ (2.4 μL, 5 mM in H_2O) in NMM buffer (62.4 μL, pH 9) with 30% 2,2,2-trifluoroethanol (36 μL) at 37 °C for 18 h. The reactions were quenched with 1 μL 0.35

M EDTA. Internal standard for each boronic acid was added (2.02 μL, 12 mM in DMSO) and the yield was determined by RP-HPLC analysis (20-60% MeCN over 28 min) at various absorbance.

Preparation of Reagents

Synthesis of known compounds:

2-amino-N,N-dimethyl-benzenesulfonamide (**19**) was prepared according to a previously reported protocol.[2]
N-benzylbenzenesulfonamide (**S4**) was prepared according to a previously reported protocol.[3]
3-(chlorosulfonyl)-4- (methylamino)-benzoic acid (**S10**) was prepared according to a previously reported protocol.[4]

***N*-benzyl-2-(4,4,5,5-tetramethyl-1,3,2-dioxaborolan-2-yl)benzenesulfonamide (S5)**

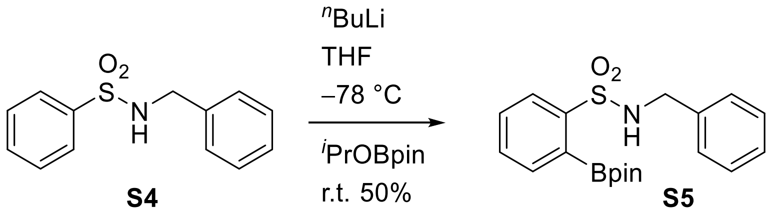

To a solution of **S4** (742 mg, 3.00 mmol)[2] in dry THF (7 mL) at −78 °C was added *n*-butyllithium (1.4 M solution in hexane, 4.7 mL, 6.63 mmol). After stirring at −78 °C for 3 h, 2-isopropoxy-4,4,5,5-tetramethyl-1,3,2-dioxaborolane (2.45 mL, 12.0 mmol) was added. The resulting solution was warmed to rt and stirred for 2.5 h. After quenching with with H_2O (10 mL) and sat. aq. NH_4Cl (20 mL), the mixture was extracted with AcOEt (20 mL x 3). The combined organic layers were washed with brine (20 mL), dried over Na_2SO_4, filtered, and evaporated. The resulting residue was purified by silica gel column chromatography (hexane/AcOEt, 25:1 to 3:1) to afford **S5** as colorless oil (557 mg, 50%). ^{1}H NMR (600 MHz, CD_3CN): δ 7.88 (dd, J = 7.34 Hz, 1.33 Hz 1H), 7.69 (dd, J = 7.34 Hz, 1.33 Hz, 1H), 7.61-7.55 (m, 2H), 7.26-7.20 (m, 3H), 7.19-7.17 (m, 2H), 6.32 (t, J = 6.11 1H), 4.06 (d, *J* = 6.27 Hz, 2H), 1.34 (s, 12H). ^{13}C NMR (151 MHz, $CDCl_3$): δ 144.6, 138.2, 135.7, 132.7, 131.2, 129.4, 128.8, 128.7, 128.4, 86.0, 47.8, 25.0. ESI-MS calcd for $C_{19}H_{24}BNaNO_4S$ $[M+H]^+$: 374.3 found 374.2.

tert-butyl benzyl((2-(4,4,5,5-tetramethyl-1,3,2-dioxaborolan-2-yl)phenyl)sulfonyl)carbamate (S6)

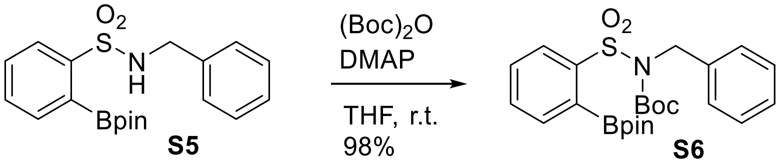

To a suspension of **S5** (557 mg, 1.49 mmol) and DMAP (18 mg, 0.15 mmol) in THF (3 mL) was added a solution of $(Boc)_2O$ (489 mg, 2.24 mmol) in THF (1 mL) at 0 °C. After stirring at r.t. overnight, the reaction mixture was evaporated. The residue was purified by silica gel column chromatography (hexane/AcOEt, 5:1) to afford **S6** as a colorless solid (695 mg, 98%). ^{1}H NMR (600 MHz, CD_3CN): δ 8.02 (d, J = 7.89, 1H), 7.67-7.65 (m, 2H), 7.60-7.59 (m, 1H), 7.43-7.37 (m, 4H), 7.31 (t, J = 7.16, 1H), 5.06 (s, 2H), 1.37 (s, 12H), 1.23 (s, 9H) ^{13}C NMR (151 MHz, DMSO-d_6): δ 151.9, 143.8, 139.3, 134.7, 133.5, 130.6, 130.2, 129.4, 128.3, 128.2, 85.6, 85.3, 51.0, 28.0, 25.1. ESI-MS calcd for $C_{24}H_{33}BNO_6S$ $[M+H]^+$: 474.4, found 474.2.

tert-butyl ((2-(4,4,5,5-tetramethyl-1,3,2-dioxaborolan-2-yl)phenyl)sulfonyl)carbamate (S7)

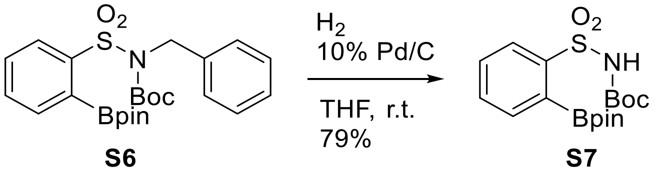

To a solution of **S6** (300 mg, 0.634 mmol) in THF (2.5 mL) was added 10% Pd on carbon (50 mg). The slurry was stirred under an H_2 atmosphere at rt overnight. The reaction mixture was filtered through Celite and the filtrate evaporated. As analysis of the crude material indicated remaining starting material, the resulting residue was again dissolved in THF (2.5 mL), and 10% Pd on carbon (102 mg) was added. The slurry was stirred under an H_2 atmosphere at rt overnight. The reaction mixture was filtered through Celite and the filtrate evaporated. The resulting residue was purified by silica gel column chromatography (hexane/AcOEt, 5:1 to 1:1) to afford **S7** as a colorless oil (193 mg, 79%). ^{1}H NMR (500 MHz, $CDCl_3$): δ 8.18 (br, 1H), 8.13 (dd, J = 1.8, 6.8 Hz, 1H), 7.83 (dd, J = 1.7, 6.5 Hz, 1H), 7.59 (ddd, J = 1.7, 6.5, 7.5 Hz, 1H), 7.56 (ddd, J = 1.8, 6.8, 7.5 Hz, 1H), 1.41 (s, 12H), 1.35 (s, 9H). ^{13}C NMR (125 MHz, $CDCl_3$): δ 149.6, 143.2, 136.0, 132.5, 130.4, 129.4, 85.3, 83.6, 28.0, 24.9. ESI-MS calcd for $C_{17}H_{25}BNO_6S$ $[M–H]^–$: 382.1, found 382.1.

N-(prop-2-yn-1-yl)-2-(4,4,5,5-tetramethyl-1,3,2-dioxaborolan-2-yl)benzenesulfonamide (1o)

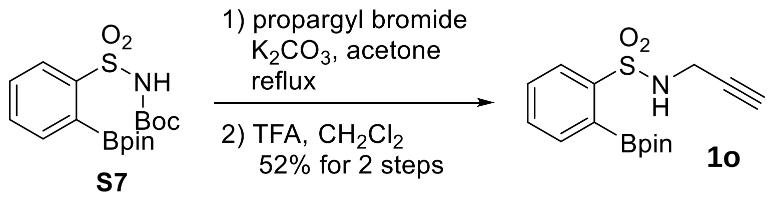

To a solution of **S7** (89 mg, 0.23 mmol) in acetone (1.5 mL) were added propargyl bromide (19 µL, 0.252 mmol) and K_2CO_3 (39 mg, 0.28 mmol). The reaction mixture was heated to reflux overnight. The reaction mixture was diluted with AcOEt (20 mL), and the mixture was washed with H_2O (10 mL) and brine (10 mL). The organic layer was dried over Na_2SO_4, filtered, and evaporated to obtain a colorless oil (86 mg), which was used for the next reaction without further purification.

To a solution of crude sulfonamide (86 mg) in CH_2Cl_2 (1.5 mL) was added TFA (1 mL). The reaction mixture was stirred at r.t. for 4 h and was evaporated. The resulting residue was purified by silica gel column chromatography (hexane/AcOEt, 5:1) to afford **1o** as a colorless oil (39 mg, 52% for 2 steps). ^{1}H NMR (600 MHz, CD_3CN): δ 1H NMR δ 7.92-7.91 (m, 1H), 7.72-7.71 (m, 1H), 7.63-7.58 (m, 2H), 6.3 (t, J = 5.70, 1H), 3.78 (dd, J = 5.91, 2.50, 2H), 2.24 (t, J = 2.50, 1H), 1.39 (s, 12H). ^{13}C NMR (151 MHz, CD_3CN) δ 114.1, 135.2, 132.5, 130.8, 128.4, 85.7, 79.1, 73.2, 32.9, 24.6. ESI-MS calcd for $C_{15}H_{21}BNO_4S$ $[M+H]^+$: 322.2, found 322.1.

4-bromo-3-(chlorosulfonyl)benzoic acid (S8)

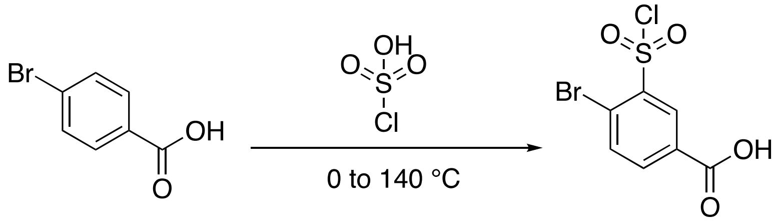

S8

Adapting from a previous procedure,[5] to a round bottom flask was added chlorosulfonic acid (9.9 mL, 149.2 mmol). The flask was cooled to 0 °C, and 4-bromobenzoic acid (2 g, 9.95 mmol) was added slowly. The mixture was allowed to warm to rt and heated at 140 °C for 19 h under nitrogen. After 19 h, the mixture was allowed to cool to rt and was poured over ice slowly. The precipitate was washed with distilled H_2O, and dried under vacuum to afford a off-white solid (2.6 g, 87%). ^{1}H NMR (600 MHz, DMSO-d_6) δ 8.45 (d, 1H, J = 2.1 Hz), 7.74–7.69 (m, 2H). ^{13}C NMR (151 MHz, DMSO-d_6): δ 166.6, 147.2, 134.5, 130.7, 129.9, 129.4, 124.9. ESI-MS: calcd for $C_7H_4BrClO_4S$ $[M-H]^-$ 296.9, found 296.9.

4-bromo-3-(*N*-methylsulfamoyl)benzoic acid (S9)

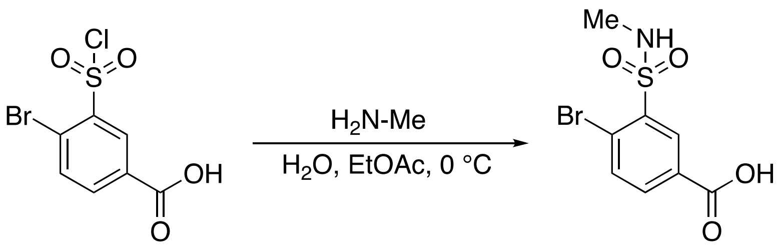

S8 **S9**

Compound **S8** (500 mg, 1.67 mmol) was dissolved in ethyl acetate (4 mL). Methylamine (40% in water, 3.0 mL) was added, and the mixture was stirred at 0 °C for 3 h. The mixture was neutralized with 4M HCl in dioxane. The reaction was extracted with ethyl acetate (10 mL x 2). The organic phase was then washed with brine (5 mL x 2), dried with Na_2SO_4. The solvent was removed under reduced pressure. The crude material was recrystallized from acetonitrile to afford orange crystals (320.7 mg, 59%). ^{1}H NMR (600 MHz, Acetone-*d*$_6$) δ 8.70 (d, 1H, J = 2.09 Hz), 8.13 (dd, 1H, J = 8.19, 2.09 Hz), 8.03 (d, 1H, J = 8.19 Hz), 6.76 (d, 1H, J = 4.35 Hz), 2.64 (d, 3H, J = 5.1 Hz). ^{13}C NMR (151 MHz, Acetone-*d*$_6$): δ 166.0, 140.2, 136.9, 135.1, 133.2, 131.4, 125.6, 29.3. ESI-MS: calcd for $C_8H_8BrNO_4S$ $[M-H]^-$ 291.9, found 291.9.

(4-(but-3-yn-1-ylcarbamoyl)-2-(N-methylsulfamoyl)phenyl)boronic acid (1p)

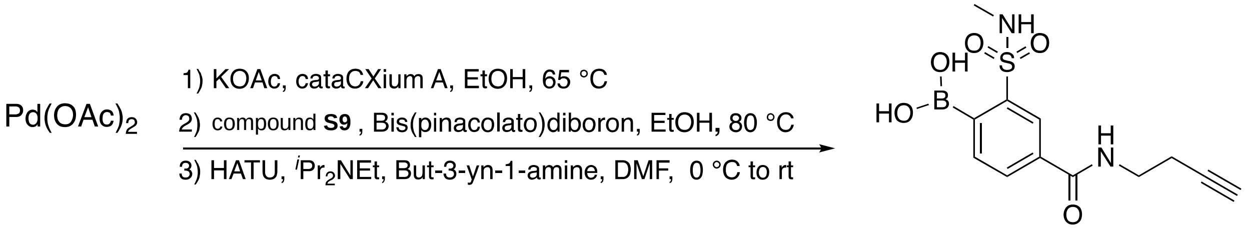

1p

*Suzuki coupling between compound **S9** and B$_2$pin$_2$*[6]

To a 20-mL vial was added $Pd(OAc)_2$ (0.79 mg, 0.0035 mmol), oven-dried KOAc (52.4 mg, 0.53 mmol), and cataCXium A (6.6 mg, 0.0184 mmol). The mixture was purged with N_2 gas three times. Degassed EtOH (2 mL) was added to the mixture, and the mixture was heated at 65 °C for 1 h. A mixture of compound **S9** (40 mg, 0.14 mmol) and B_2pin_2 (69.1 mg, 0.27 mmol) was purged with N_2 gas and then dissolved in degassed EtOH (0.8 mL) before adding to the reaction. The reaction was heated at 80 °C for 20 h. After 20 h, the reaction was quenched with 1 M HCl (12 mL), and extracted with ethyl acetate (10 mL x 3). The organic layer was washed with brine (15 mL), and dried with Na_2SO_4. The solvent was removed under reduced pressure, and the crude material was dried under vacuum and used in the next step without further purification.

HATU coupling with But-3-yn-1-amine

HATU (74.5 mg, 0.196 mmol) was added to the crude material and the mixture was dissolved by DMF (0.7 mL). *N,N*-diisopropylethylamine (97.6 μL, 0.56 mmol) was added at 0 °C, the mixture was stirred at rt for 10 min. But-3-yn-1-amine (13.8 μL, 0.168 mmol) was added at 0 °C, and the reaction was allowed to warm to rt and stirred for 24 h. The solvent was removed by a gentle flow of N_2 gas, and the crude material was purified by reverse-phase HPLC (10 - 30%

MeCN over 22 min). Lyophilization of the collected fraction afford **1p** as a white solid (7.6 mg, 18%) The product was contaminated with pinacol. ^{1}H NMR (600 MHz, MeOD-d_4) δ 8.89 (t, 1H, J = 4.6 Hz), 8.27 (d, 1H, J = 1.2 Hz), 8.03 (dd, 1H, J = 7.8, 1.5 Hz), 7.58 (d, 1H, J = 7.8 Hz), 3.54 (dt, 2H, J = 5.8, 6.9 Hz), 3.35 (s, 2H), 2.56 (s, 3H), 2.52 (td, 2H, J = 7.1, 2.7 Hz), 2.33 (t, 1H, J = 2.7 Hz).
^{13}C NMR (151 MHz, MeOD-d_4): δ 168.8, 143.8, 136.81, 136.77, 133.0, 131.3, 127.0, 82.3, 71.1, 40.4, 29.3, 19.9. ESI-MS: calcd for $C_{12}H_{15}BN_2O_5S$ $[M+H]^+$ 311.1, found 311.1.

4-(methylamino)-3-(N-methylsulfamoyl)benzoic acid (S11)

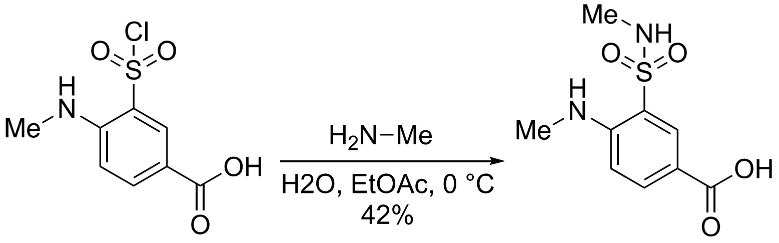

S10 **S11**

Compound **S11** (500 mg, 2.00 mmol) was dissolved in ethyl acetate (5 mL). Methylamine (40% in water, 4.0 mL) was added, and the mixture was stirred at 0 °C for 3 h. The mixture was neutralized with 4M HCl in dioxane. The reaction was extracted with ethyl acetate (10 mL x 2). The organic phase was then washed with brine (5 mL x 2), dried with Na_2SO_4. The solvent was removed under reduced pressure. The crude material was washed with cold acetonitrile to afford white solid (203.4 mg, 42%). ^{1}H NMR (600 MHz, DMSO-d_6) δ 12.59 (s, 1H), 8.15 (d, 1H, J = 2.1 Hz), 7.93 (dd, 1H, J = 8.8, 2.0 Hz), 7.57 (dt, 1H, J = 5.1, 4.9 Hz), 6.81 (d, 1H, J = 8.9 Hz), 6.48 (dt, 1H, J = 4.9, 4.7 Hz), 2.88 (d, 3H, J = 4.9 Hz), 2.37 (d, 3H, J = 5.0 Hz). ^{13}C NMR (151 MHz, DMSO-d_6): δ 166.6, 149.3, 135.0, 131.9, 118.7, 116.3, 111.2, 29.9, 28.3. ESI-MS: calcd for $C_9H_{12}N_2O_4S$ $[M-H]^-$ 243.1, found 243.1.

***N*-(but-3-yn-1-yl)-4-(methylamino)-3-(*N*-methylsulfamoyl)benzamide (S12)**

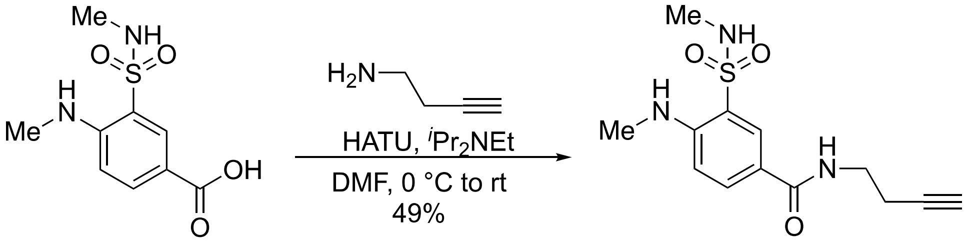

S11 **S12**

Compound **S11** (34.1 mg, 0.140 mmol), and HATU (74.5 mg, 0.196 mmol) were dissolved by DMF (0.7 mL). *N,N*-diisopropylethylamine (97.5 μL, 0.560 mmol) was added at 0 °C, the mixture was stirred at rt for 10 min. But-3-yn-1-amine (45.7 μL, 0.559 mmol) was added at 0 °C, and the reaction was allowed to warm to rt and stirred for 20 h. The solvent was removed by a gentle flow of N_2 gas, and the crude material was purified by reverse-phase HPLC (20 - 60% MeCN

over 28 min) to afford **S12** as a clear oil (20.1 mg, 49%). ^{1}H NMR (600 MHz, Acetone-d_6) δ 8.21 (d, 1H, J = 2.2 Hz), 8.03 (br s, 1H), 8.00 (dd, 1H, J = 8.8, 2.2 Hz), 6.86 (d, 1H, J = 8.8 Hz), 6.48 (br s, 1H), 3.53 (dt, 2H, J = 6.0, 7.1 Hz), 2.96 (s, 3H), 2.51 (s, 3H), 2.49 (td, 2H, J = 7.2, 2.7 Hz), 2.41 (t, 1H, J = 2.7 Hz). ^{13}C NMR (151 MHz, Acetone-d_6): δ 166.6, 149.9, 133.9, 130.7, 121.7, 120.2, 111.9, 82.7, 71.0, 39.8, 30.3, 29.3, 19.9. ESI-MS: calcd for $C_{13}H_{17}N_3O_3S$ $[M+H]^+$ 296.1, found 296.1.

Supplementary Data

For Figure 1

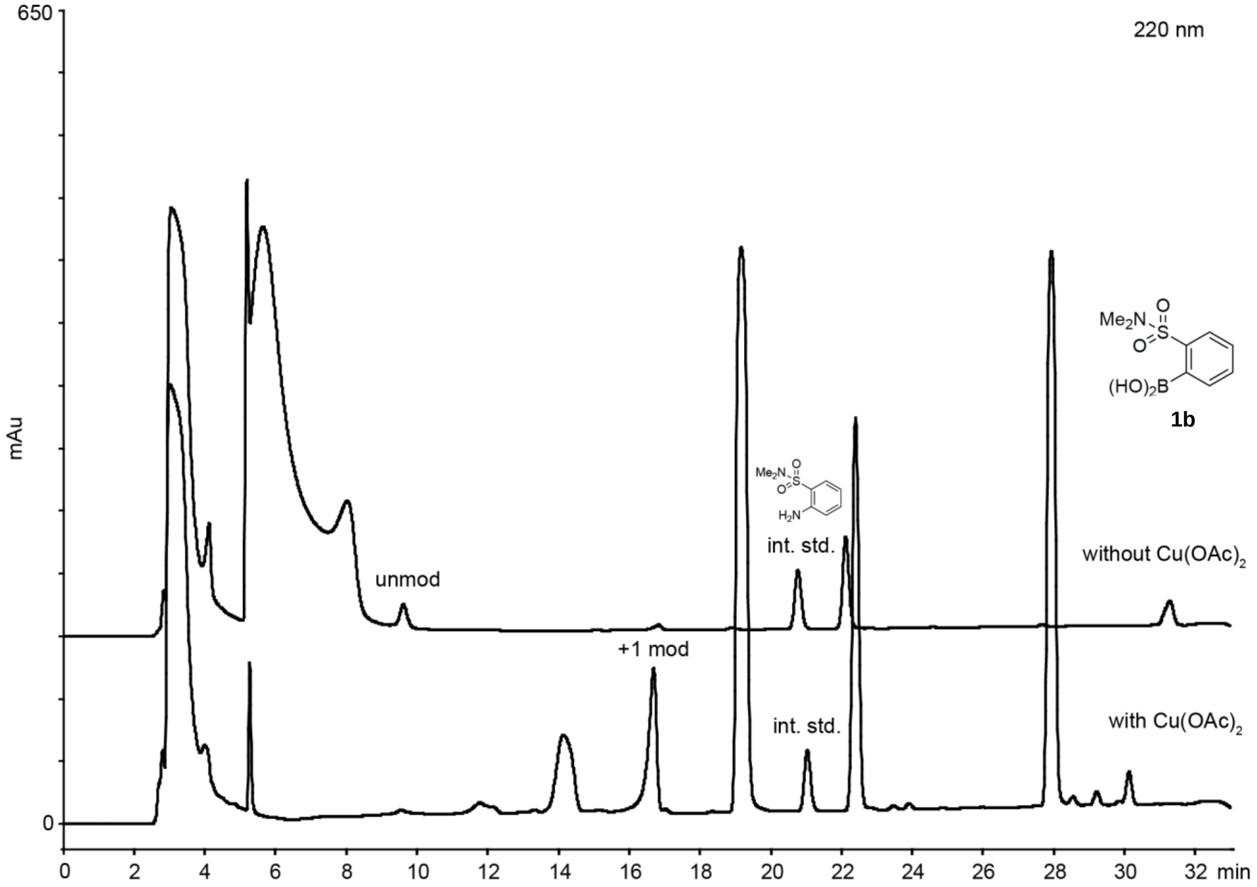

Figure S4. RP-HPLC trace of the reaction between bradykinin (**2**) and **1b** without and with $Cu(OAc)_2$ at 220 nm. Ramp: 20-60% MeCN 28 mins.

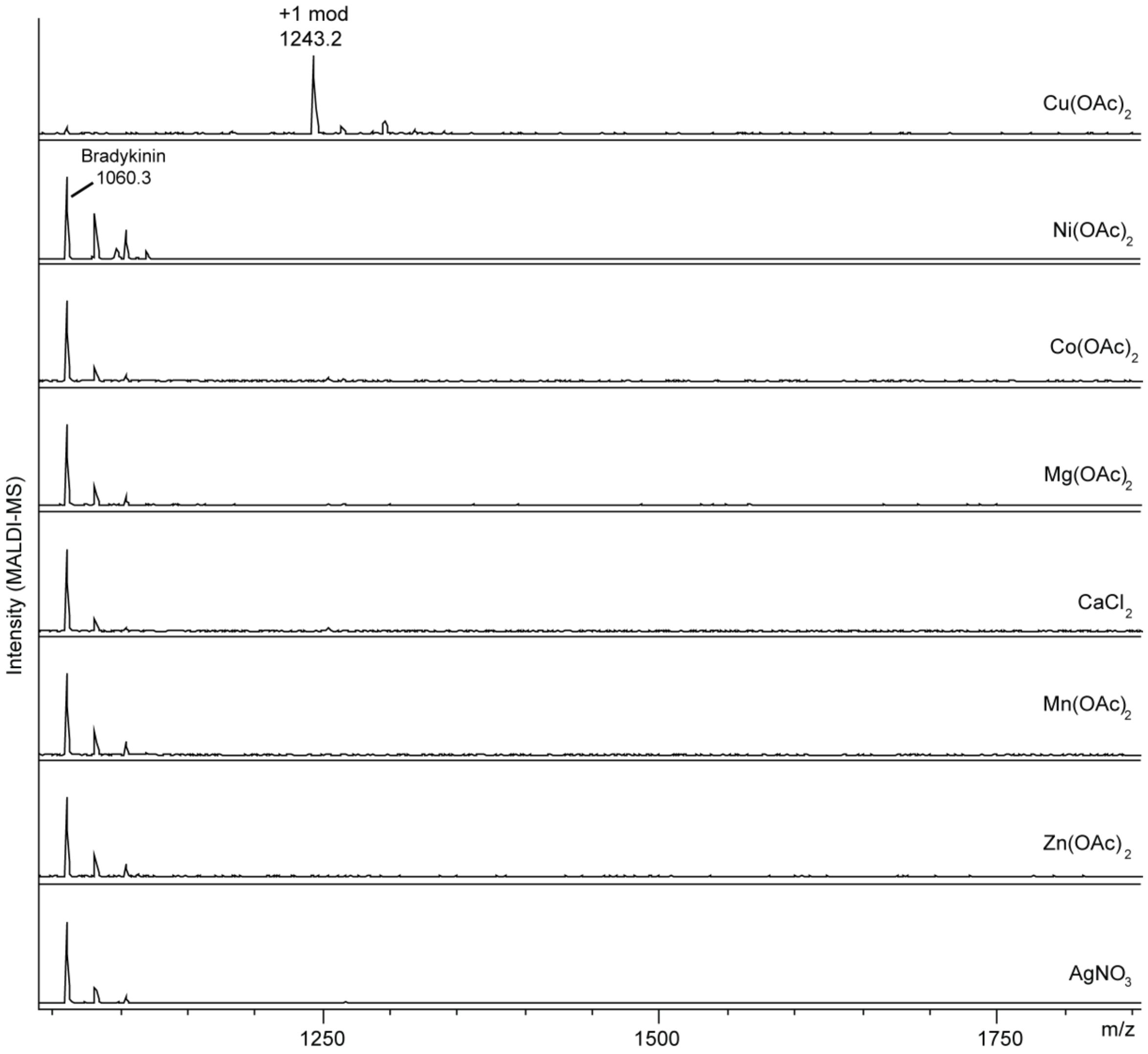

Figure S5. MALDI-MS spectra for the crude reaction mixture of bradykinin **2** with various metal salts and 2-(N,N-dimethylsulfonamide)phenylboronic acid (**1b**). Unmodified peptide was observed at 1060.3 ($[M+H]^+$). Modified peptide was observed at 1243.2. Sinapic acid was used as a matrix.

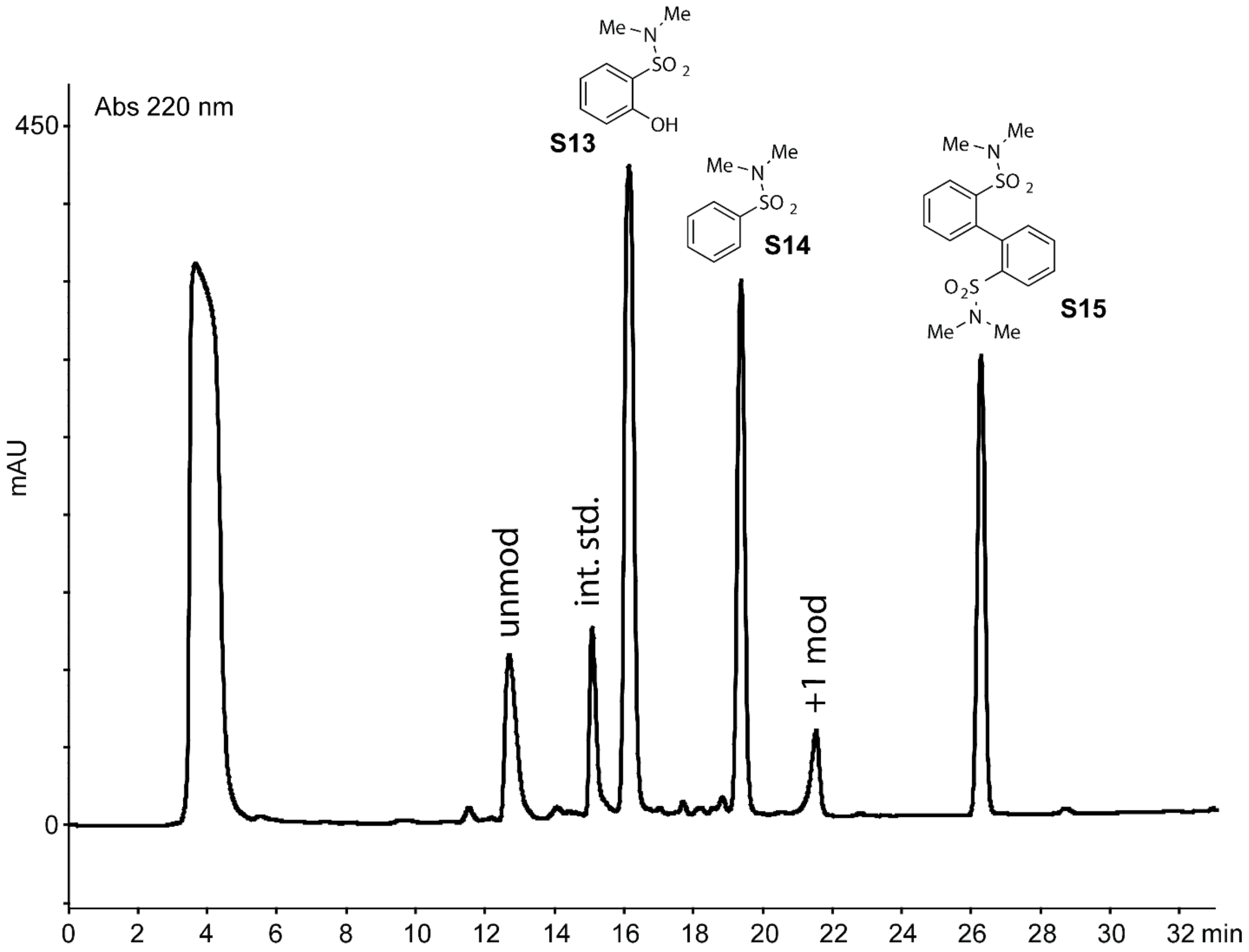

Figure S6. RP-HPLC trace of reaction between Bradykinin (**2**) and (2-(N,N-methylsulfamoyl)phenyl)boronic acid (**1b**) mediated by copper. Angiotensin IV (**5**) peptide was added as internal standard after the reaction was quenched. Ramp: 20-60% MeCN 28min. Side products (**S13**, **S14**, and **S15**) were isolated and characterized by ^{1}H NMR spectroscopy. ^{1}H spectra of *N*,*N*-dimethylbenzenesulfonamide (**S14**) and N^2,N^2,$N^{2'}$,$N^{2'}$-tetramethyl-[1,1'-biphenyl]-2,2'-disulfonamide (**S15**) are consistent with previous literature.[7, 8]

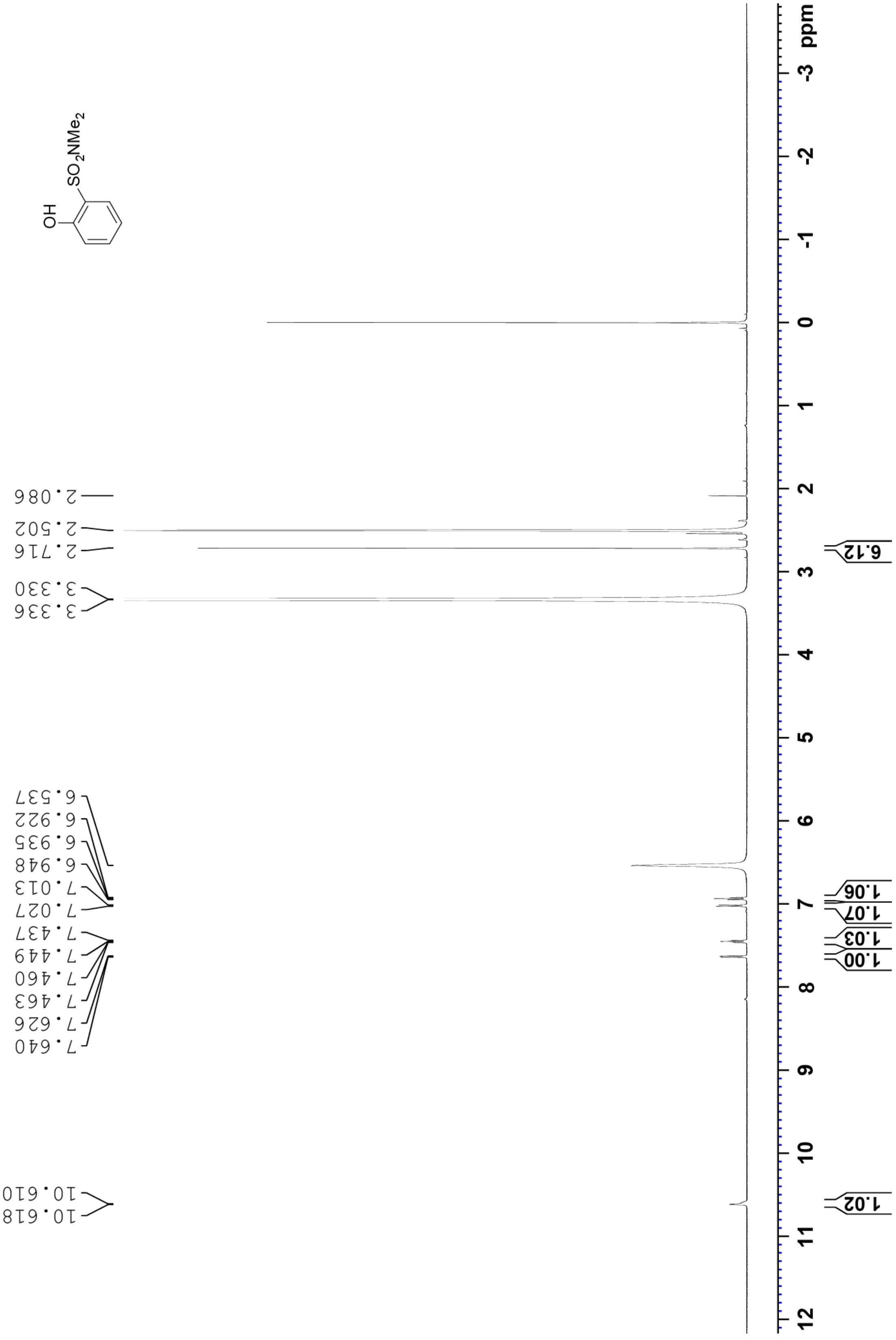

Figure S7. ^{1}H NMR spectrum of 2-hydroxy-*N*,*N*-dimethylbenzenesulfonamide (**S13**) in DMSO-d_6

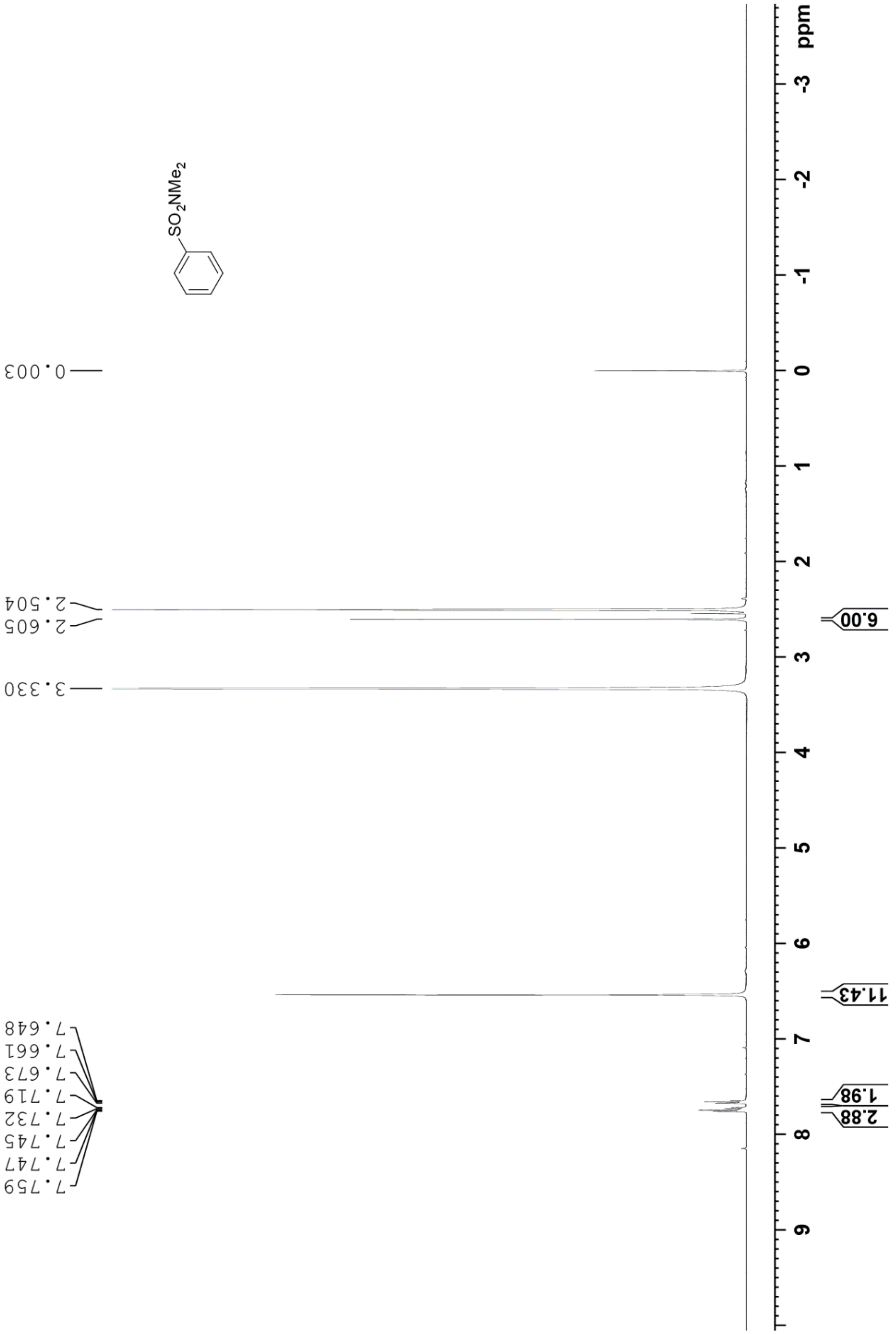

Figure S8. ^{1}H NMR spectrum of *N*,*N*-dimethylbenzenesulfonamide (**S14**) in DMSO-d_6

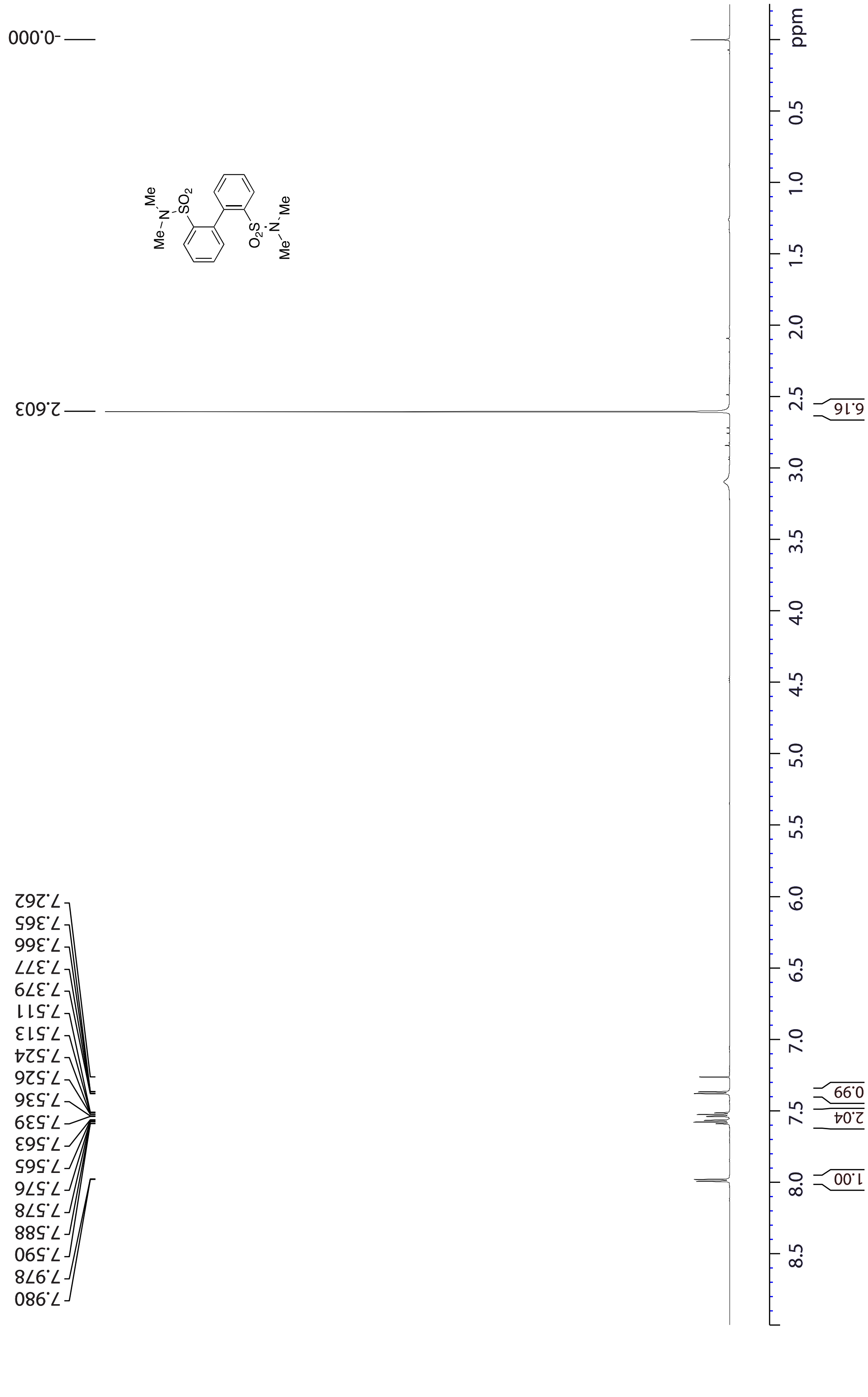

Figure S9. ^{1}H NMR spectrum of *N*^{2},*N*^{2},*N*$^{2'}$,*N*$^{2'}$-tetramethyl-[1,1'-biphenyl]-2,2'-disulfonamide (**S15**) in $CDCl_3$

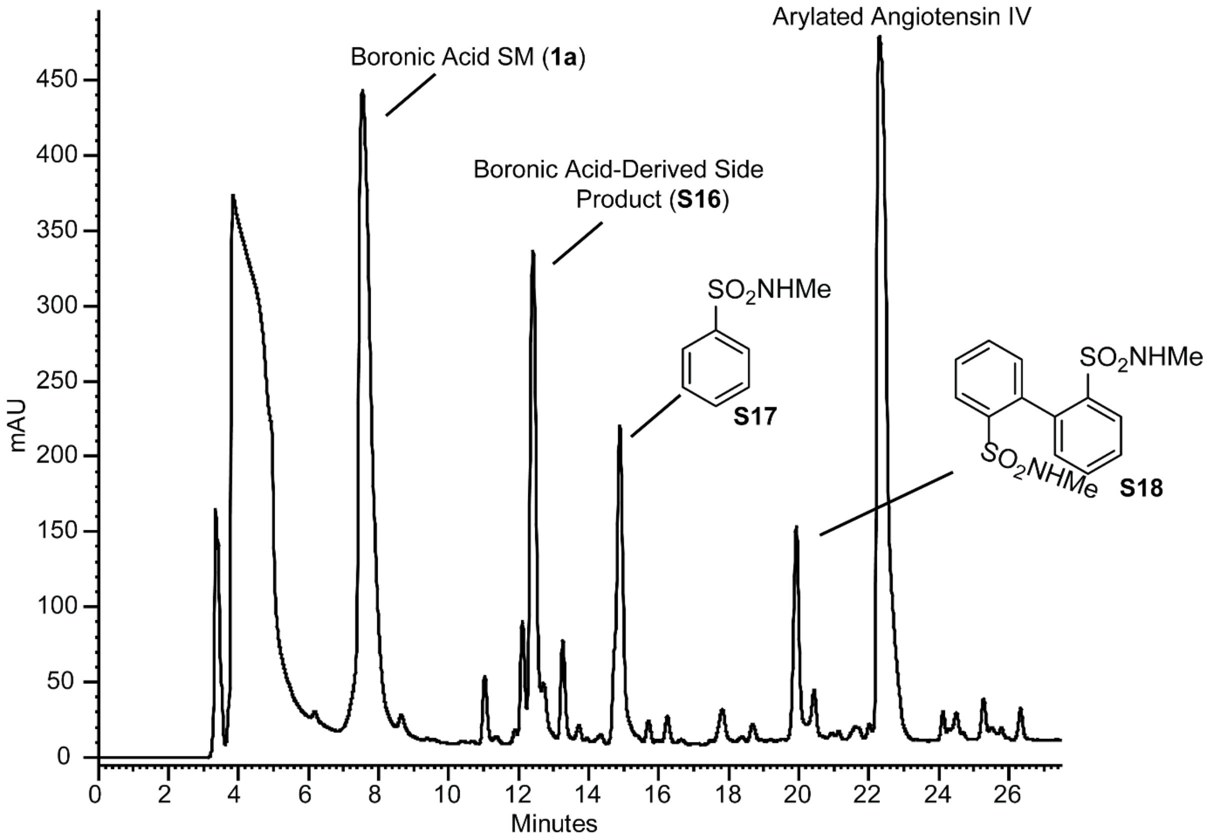

Figure S10. RP-HPLC trace of reaction between angiotensin IV (**5**) (10 mg scale) and boronic acid (**1a**) mediated by copper. Ramp: 20-60% MeCN 28min. Side products (**S16-S18**) were isolated and characterized by ESI-MS. After 18 h the reaction was quenched with EDTA and concentrated 5X by gentle flow of nitrogen before side products were purified and characterized.

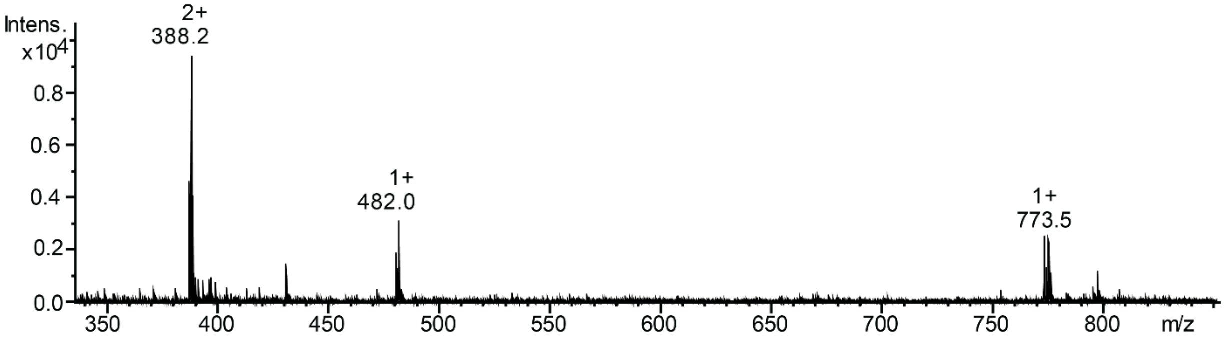

Figure S11. ESI-MS of **S16** (positive mode)

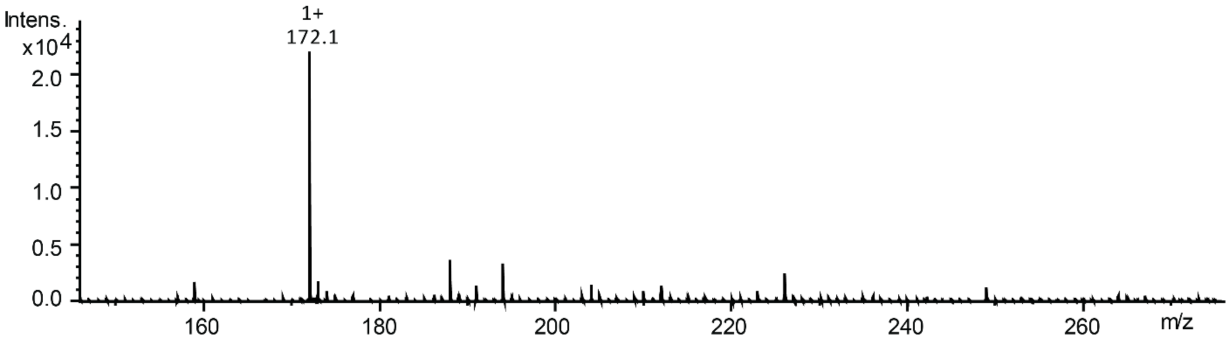

Figure S12. ESI-MS of **S17** (positive mode). m/z 172.1 corresponds to $[M+H]^+$.

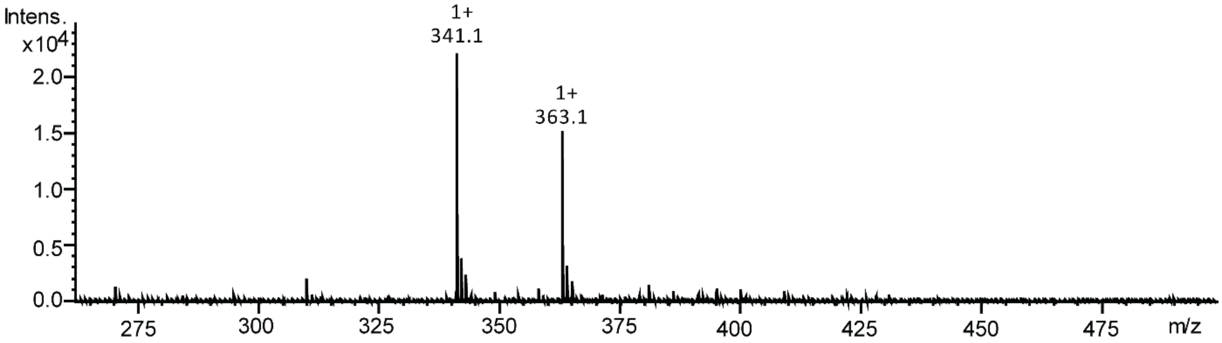

Figure S13. ESI-MS of **S18** (positive mode). m/z 341.1 and 363.1 correspond to $[M+H]^+$ and $[M+Na]^+$, respectively.

For Figure 2

Boronic Acid 1b

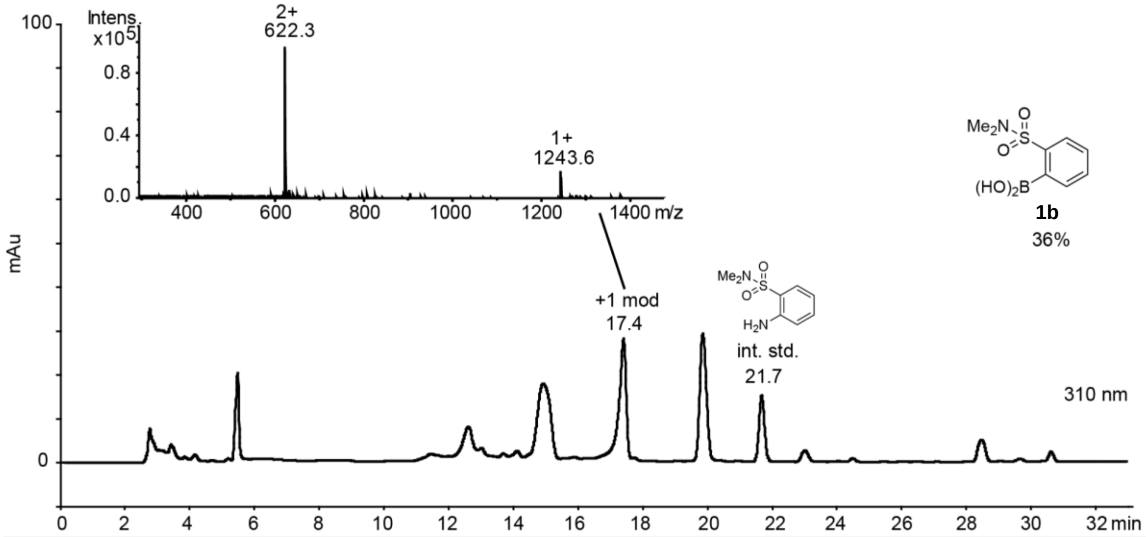

Figure S14. RP-HPLC trace of the reaction of peptide **2** with boronic acid **1b** at 310 nm. Insert: ESI-MS spectrum of modified peptide **2a**. m/z 622.3 and 1243.6 correspond to $[M+2H]^{2+}$ and $[M+H]^{+}$, respectively. Ramp: 20-60% MeCN 28 mins. * Boronic acid-derived byproduct: 2-(N,N-dimethylsulfonamidyl)-phenol.

Boronic Acid 1c

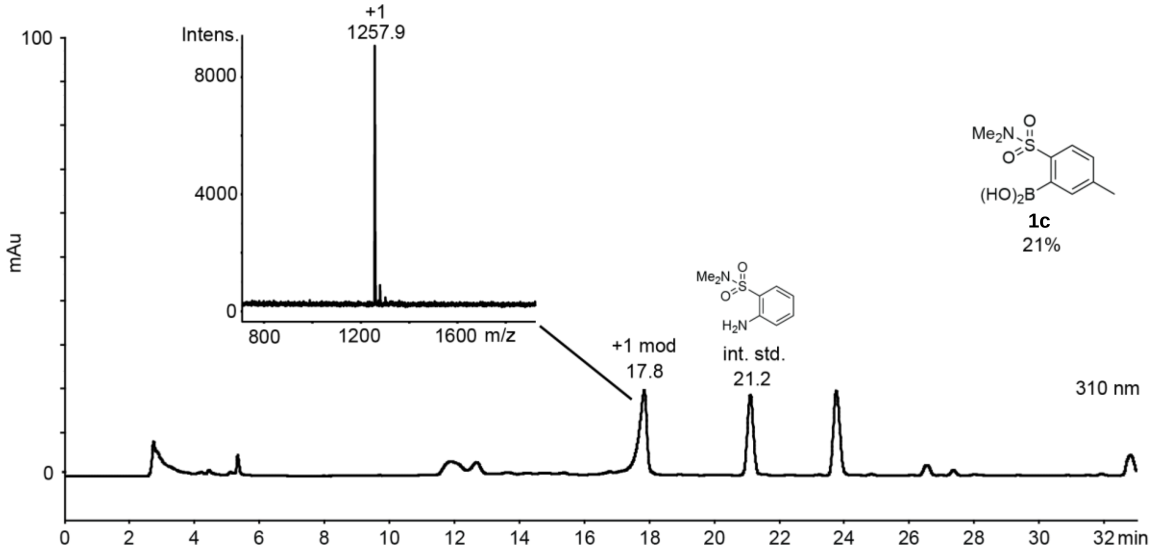

Figure S15. RP-HPLC trace of the reaction of peptide **2** with boronic acid **1c** at 310 nm. Insert: ESI-MS spectrum of modified peptide **2b**. m/z 1257.9 corresponds $[M+H]^{+}$. Ramp: 20-60% MeCN 28 mins.

Boronic Acid 2d

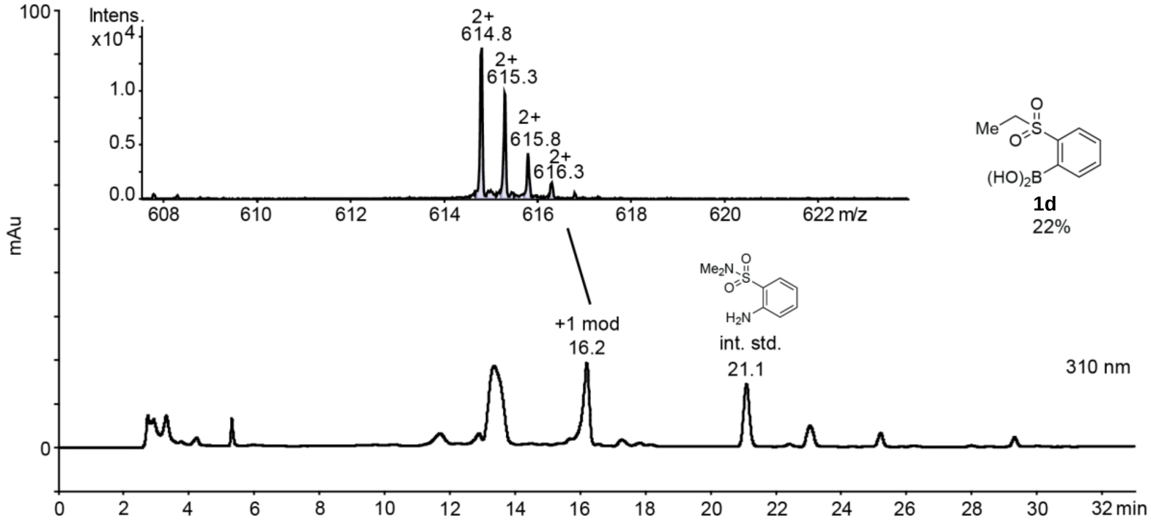

Figure S16. RP-HPLC trace of the reaction of peptide **2** with boronic acid **1d** at 310 nm. Insert: ESI-MS spectrum of modified peptide **2c**. m/z 614.8 corresponds to $[M+2H]^{2+}$. Ramp: 20-60% MeCN 28 mins.

Boronic Acid 1e (10 mg scale)

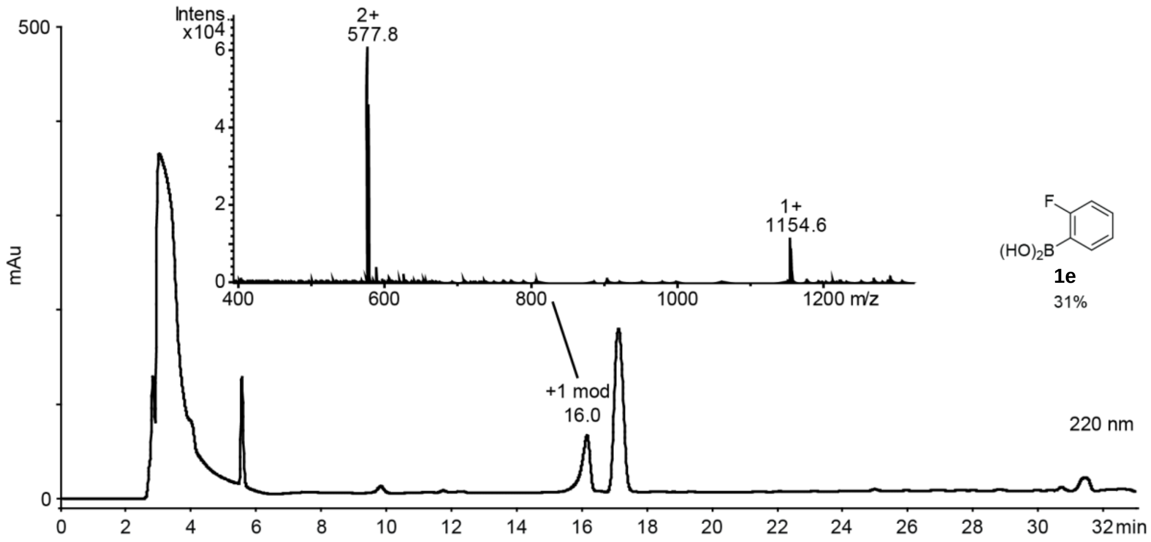

Figure S17. RP-HPLC trace of the reaction of peptide **2** with boronic acid **1e** at 220 nm. Isolated yield of a 10-mg scale reaction. Insert: ESI-MS spectrum of modified peptide **2d**. m/z 577.8 and 1154.6 correspond to $[M+2H]^{2+}$ and $[M+H]^{+}$, respectively. Ramp: 20-60% MeCN 28 mins.

Boronic Acid 1e (120 μL scale)

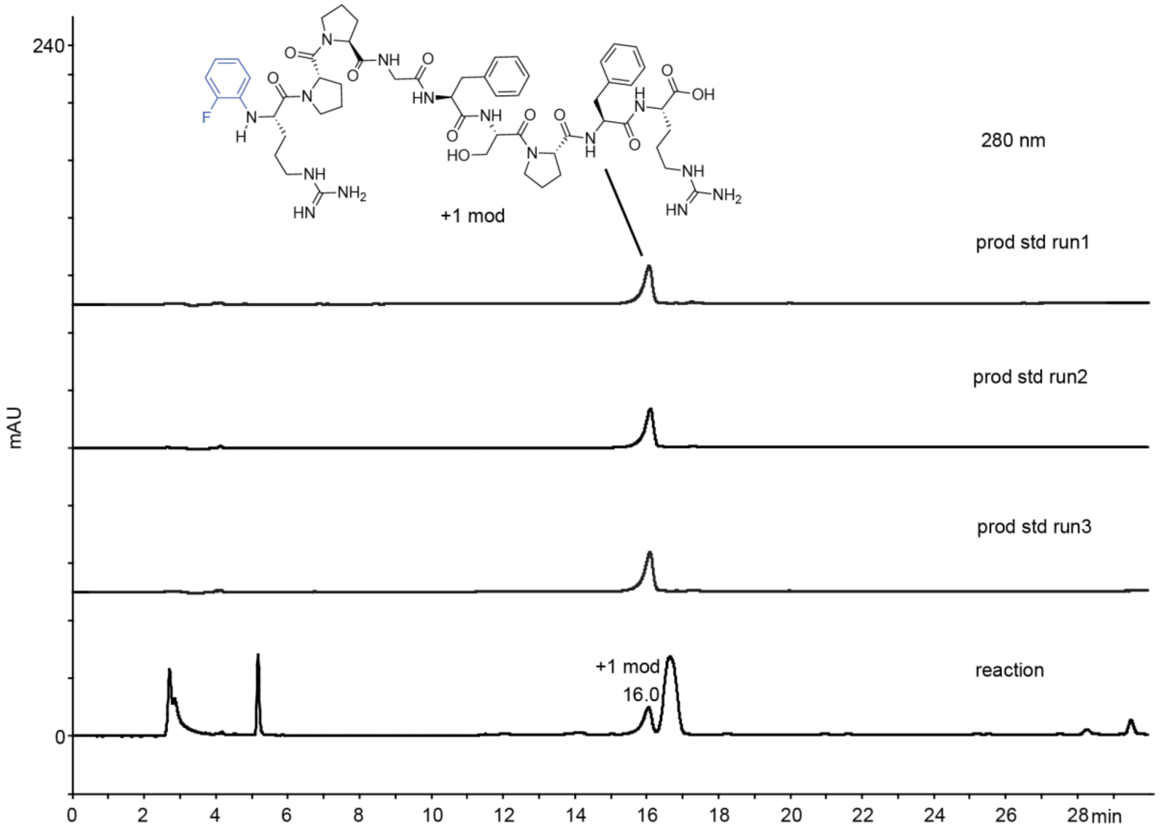

	Area in 280 nm channel	Average of run1 ,2,3	Yield
Prod std run1 (0.1 mM)	226680	229780	
Prod std run2 (0.1 mM)	229732		
Prod std run3 (0.1 mM)	232927		
Reaction (0.2 mM s.m.)	147367		32%

Figure S18. RP-HPLC trace of the reaction of peptide **2** with boronic acid **1e** at 280 nm (Total reaction volume 120 μL). Table: Peak area of 0.1 mM isolated modified peptide was calculated 3 times and an average was obtained. The average was used to calculate the concentration of modified peptide in a 0.2 mM reaction mixture. Ramp: 20-60% MeCN 28 mins.

Boronic Acid 1f

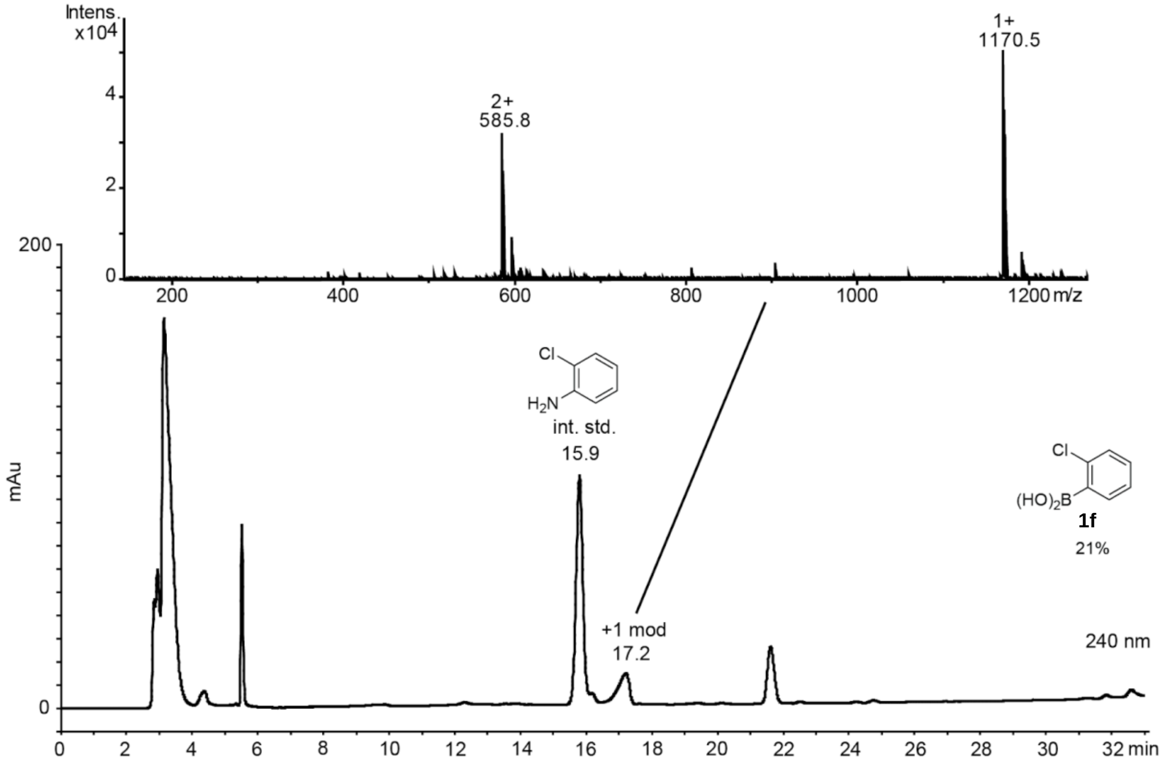

Figure S19. RP-HPLC trace of the reaction of peptide **2** with boronic acid **1f** at 240 nm. Insert: ESI-MS spectrum of modified peptide **2e**. m/z 585.8 and 1170.5 correspond to $[M+2H]^{2+}$ and $[M+H]^{+}$, respectively. Ramp: 20-60% MeCN 28 mins.

Boronic acid 1g (10 mg scale)

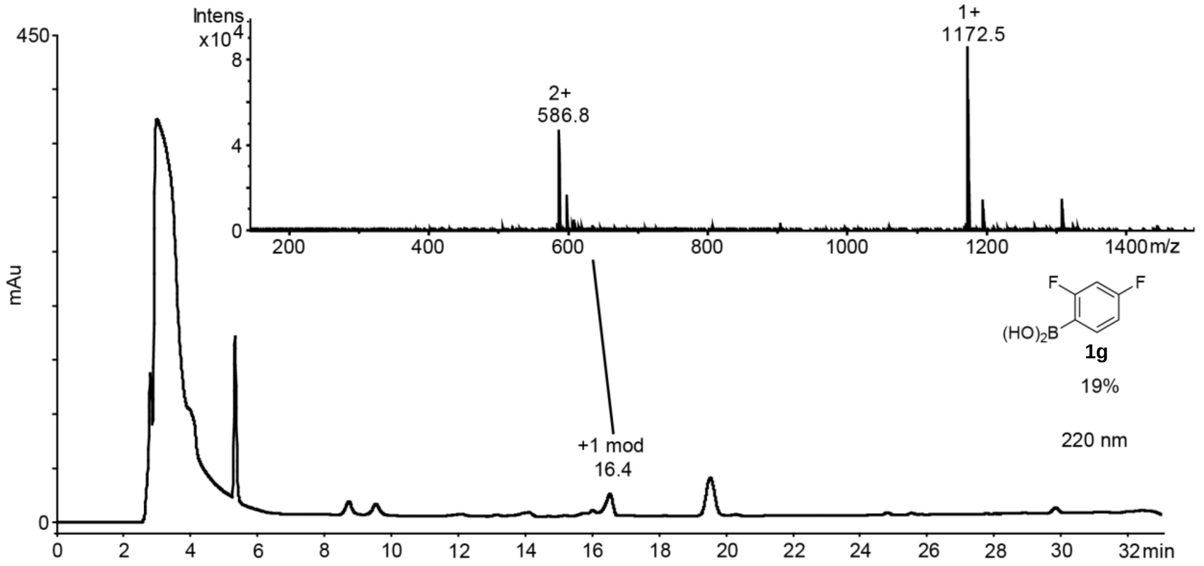

Figure S20. RP-HPLC trace of the reaction of peptide **2** with boronic acid **1g** at 220 nm. Isolated yield of a 10-mg scale reaction. Insert: ESI-MS spectrum of modified peptide **2f**. m/z 586.8 and 1172.5 correspond to $[M+2H]^{2+}$ and $[M+H]^{+}$, respectively. Ramp: 20-60% MeCN 28 mins.

Boronic Acid 1h

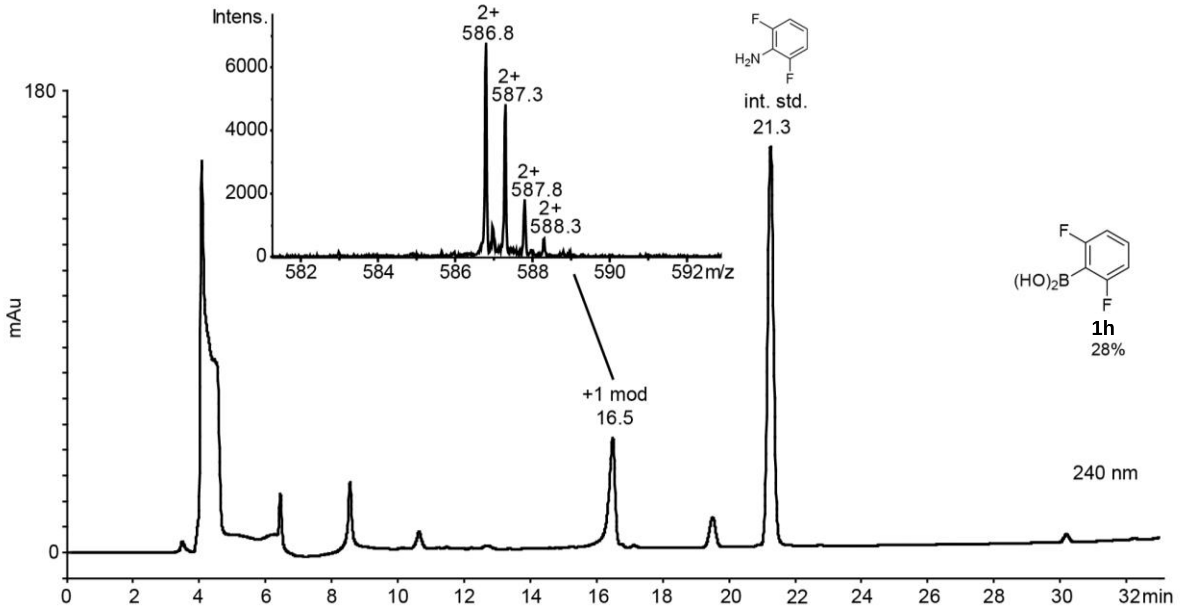

Figure S21. RP-HPLC trace of the reaction of peptide **2** with boronic acid **1h** at 240 nm. Insert: ESI-MS spectrum of modified peptide **2g**. m/z 586.8 corresponds to $[M+2H]^{2+}$. Ramp: 20-60% MeCN 28 mins.

Boronic Acid 1i

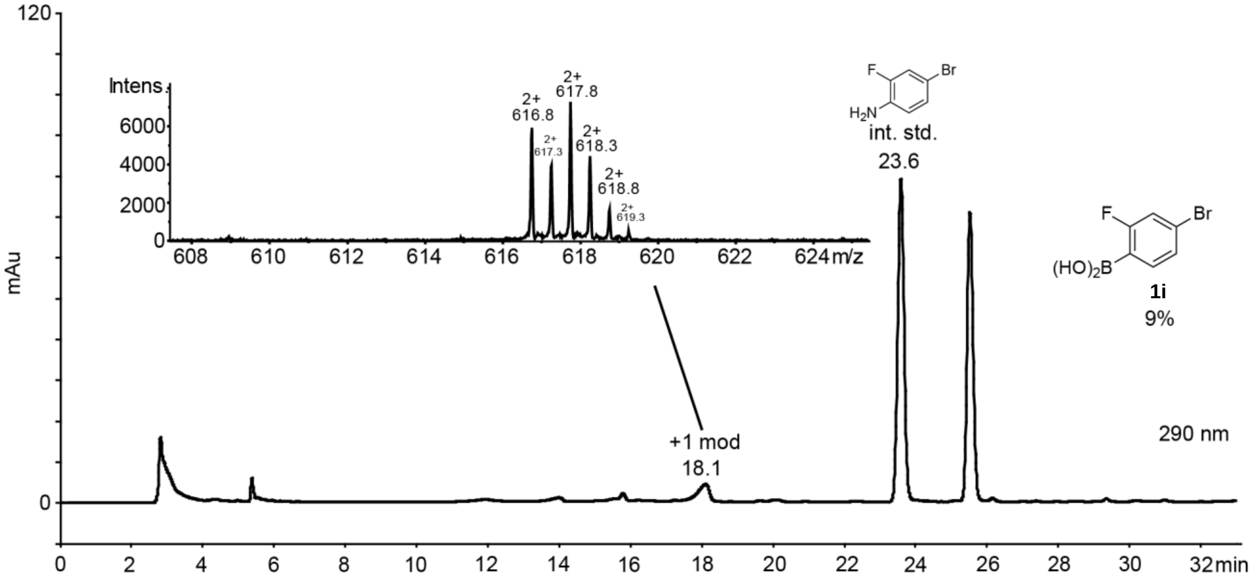

Figure S22. RP-HPLC trace of the reaction of peptide **2** with boronic acid **1i** at 290 nm. Insert: ESI-MS spectrum of modified peptide **2h**. m/z 616.8 corresponds to $[M+2H]^{2+}$. Ramp: 20-60% MeCN 28 mins.

Boronic Acids 1j-n

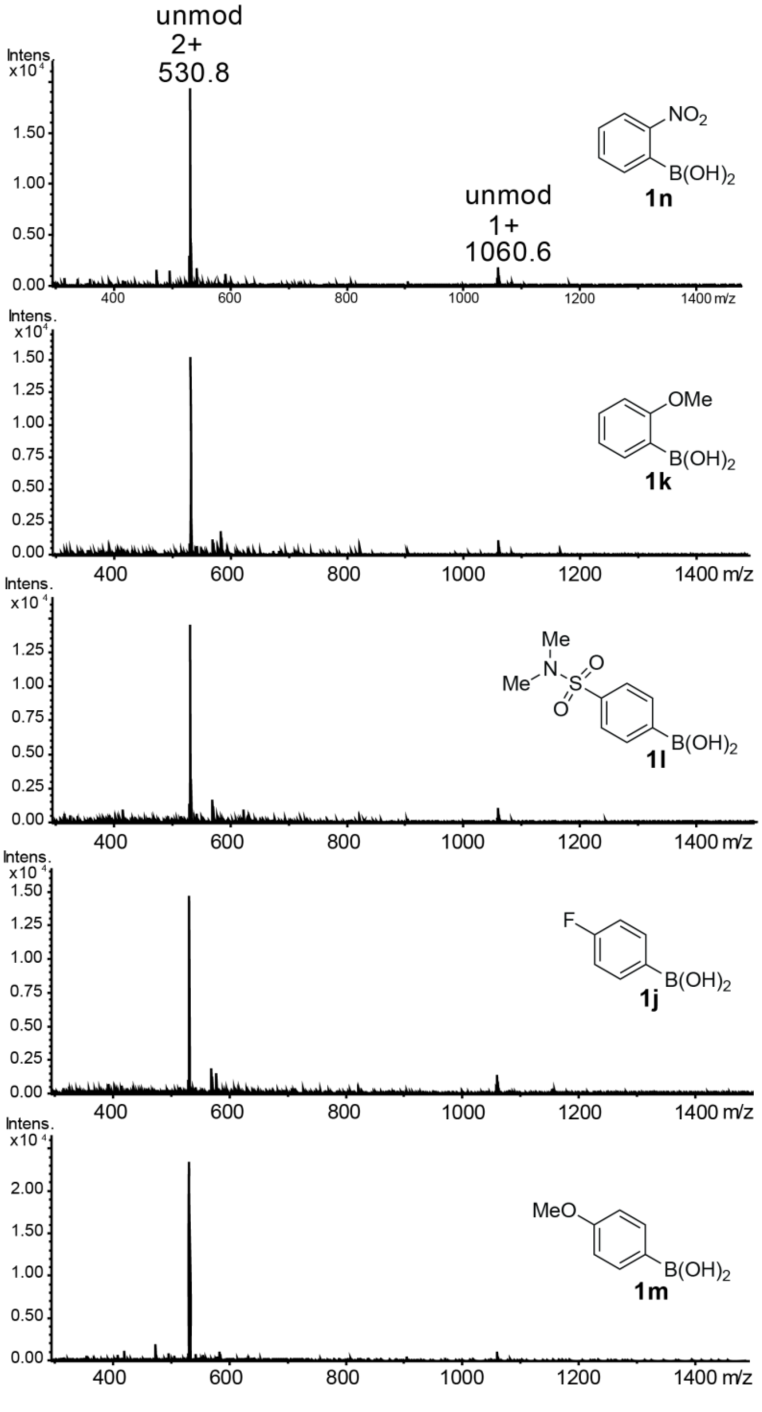

Figure S23. Copper(II)-promoted modification of Bradykinin (**2**) with phenylboronic acid derivatives **1j-1n**. Peaks at 1060.6 m/z correspond to unmodified bradykinin ("unmod") ($[M+H]^+$). Peaks at 530.8 m/z correspond to unmodified bradykinin ("unmod") ($[M+2H]^{2+}$).

For Figure 3

Modification of Angiotensin IV (5) with boronate ester 1o

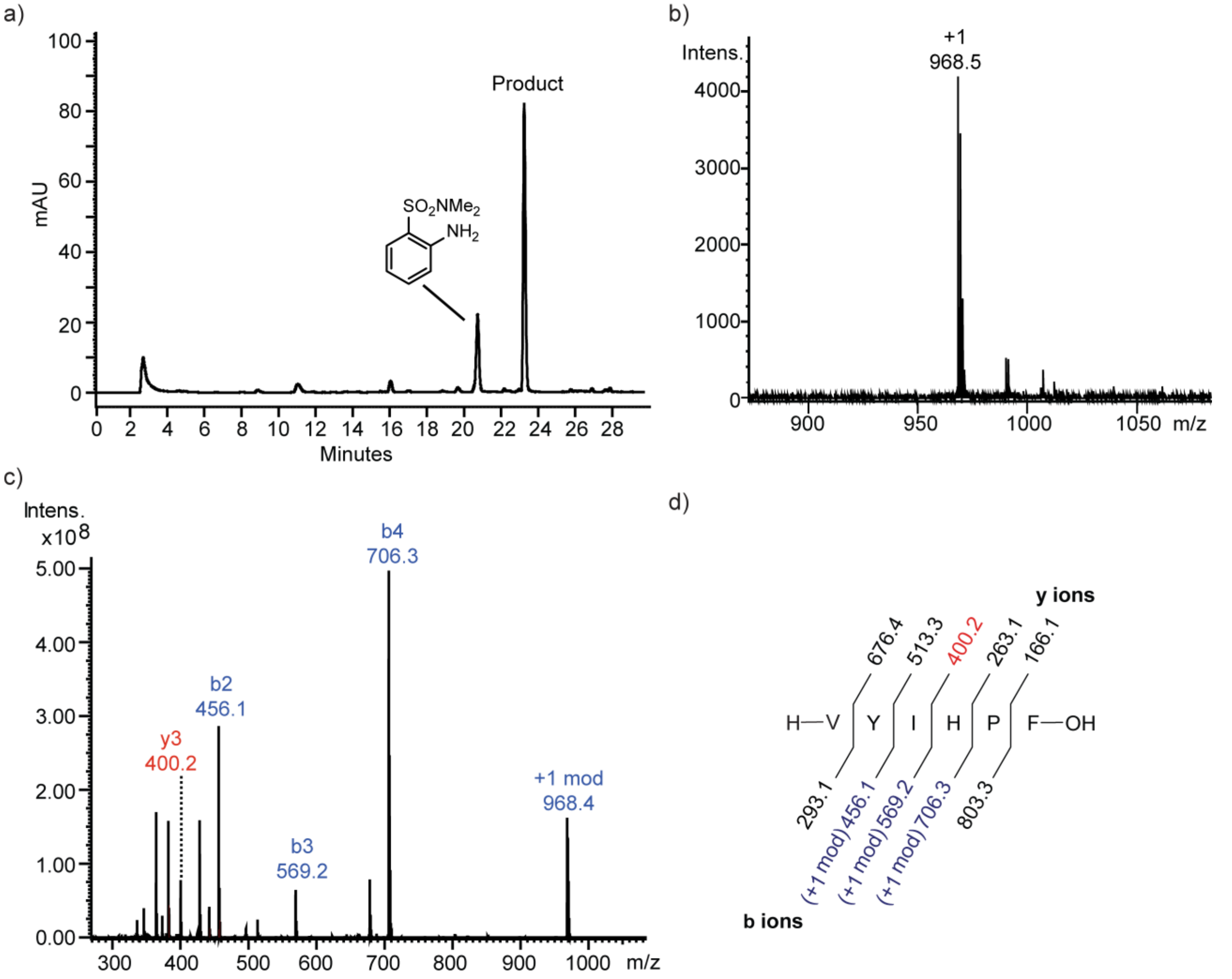

Figure S24. (a) HPLC trace for the modification of peptide **5** with boronic acid **1o** at 310 nm. Ramp: 20-60% MeCN 28 mins. (b) MALDI-MS of the crude reaction mixture of peptide **5**. Modified product **5b** was noted at 968.5 m/z. Sinapic acid was used as a matrix. (c) LC-MS/MS spectrum of peptide **5** modified with boronic acid **1o** modified at the N-terminus. The selected peaks are labeled. (d) Sequence and fragmentation ladder of peptide **5** modified with boronic acid **1o**. Observed *b* and *y* ions are indicated.

Modification of Angiotensin IV (5) with boronate ester 1p

a)

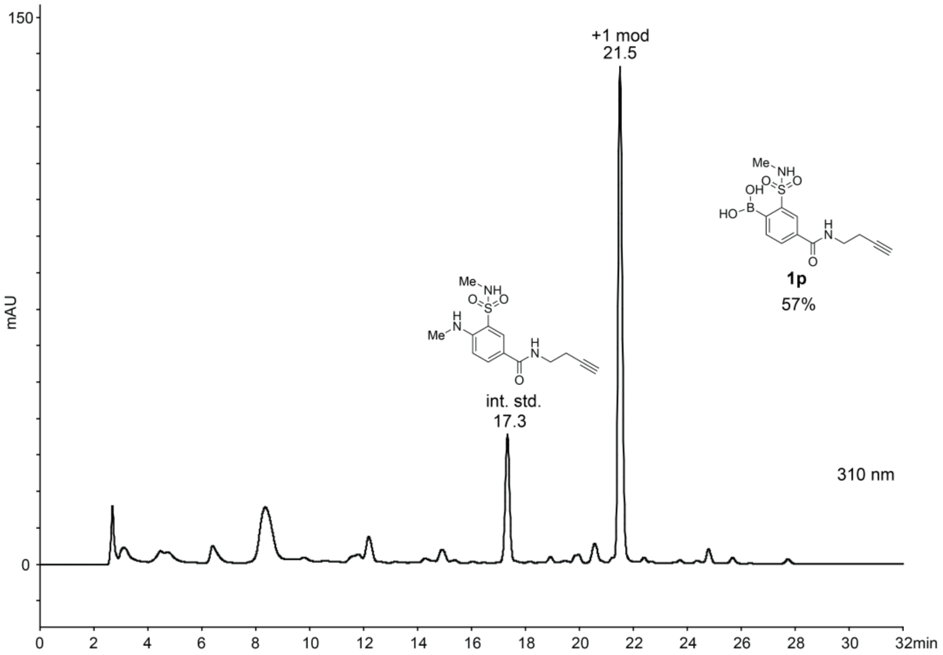

b)

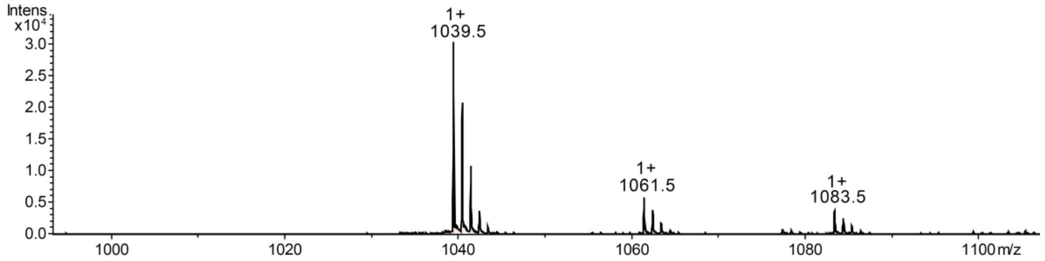

Figure S25. (a) RP-HPLC trace of the reaction of peptide **5** with boronic acid **1p** at 310 nm. Ramp: 20-60% MeCN 28 mins. (b) ESI-MS spectrum of modified peptide **5c**. m/z 1039.5 and 1061.5 corresponds to $[M+H]^+$ and $[M+Na]^+$, respectively.

For Table 1

Temperature

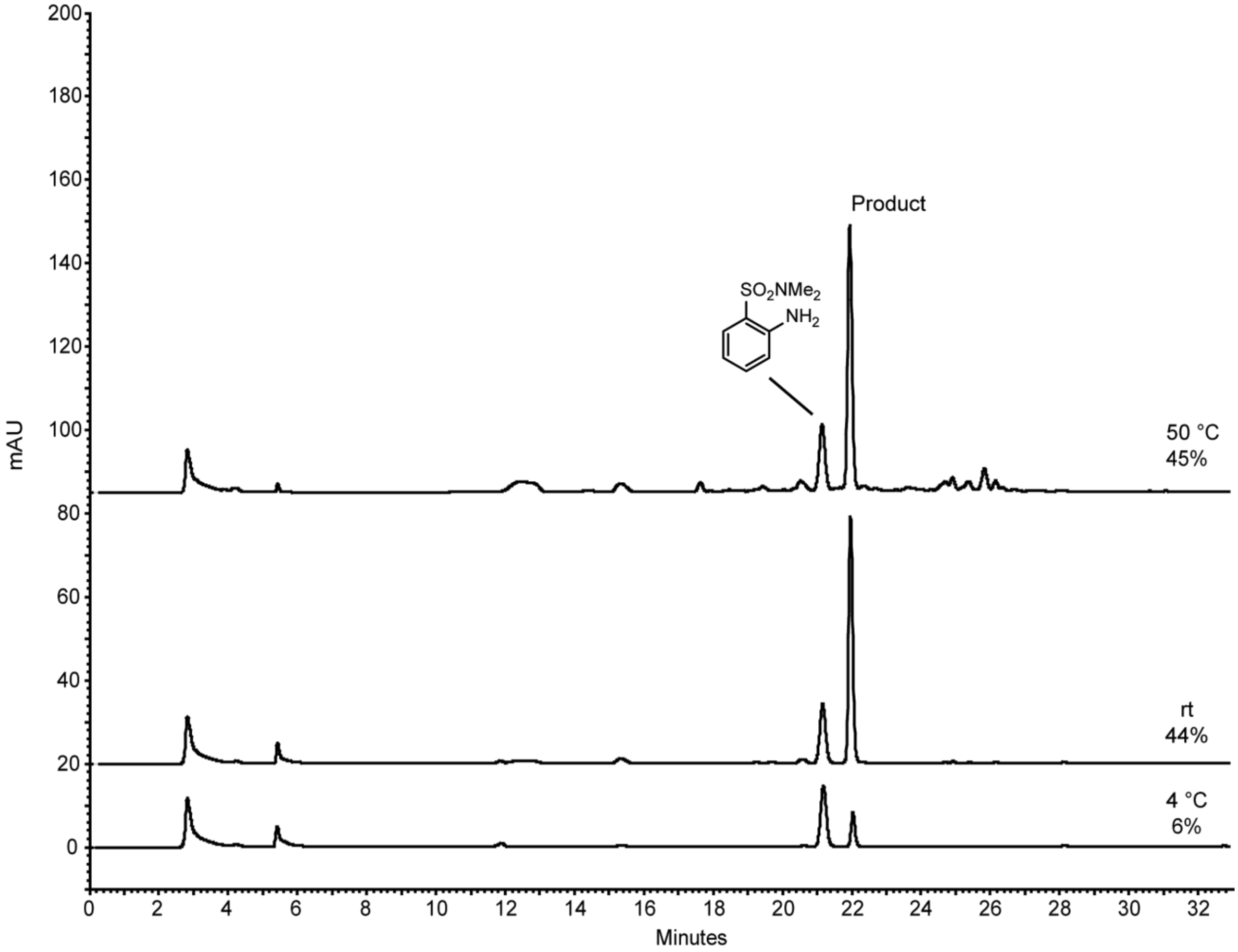

Figure S26. RP-HPLC trace of the reaction of angiotensin IV (**5**) at various temperatures at 310 nm. Ramp: 20-60% MeCN 28 mins.

pH

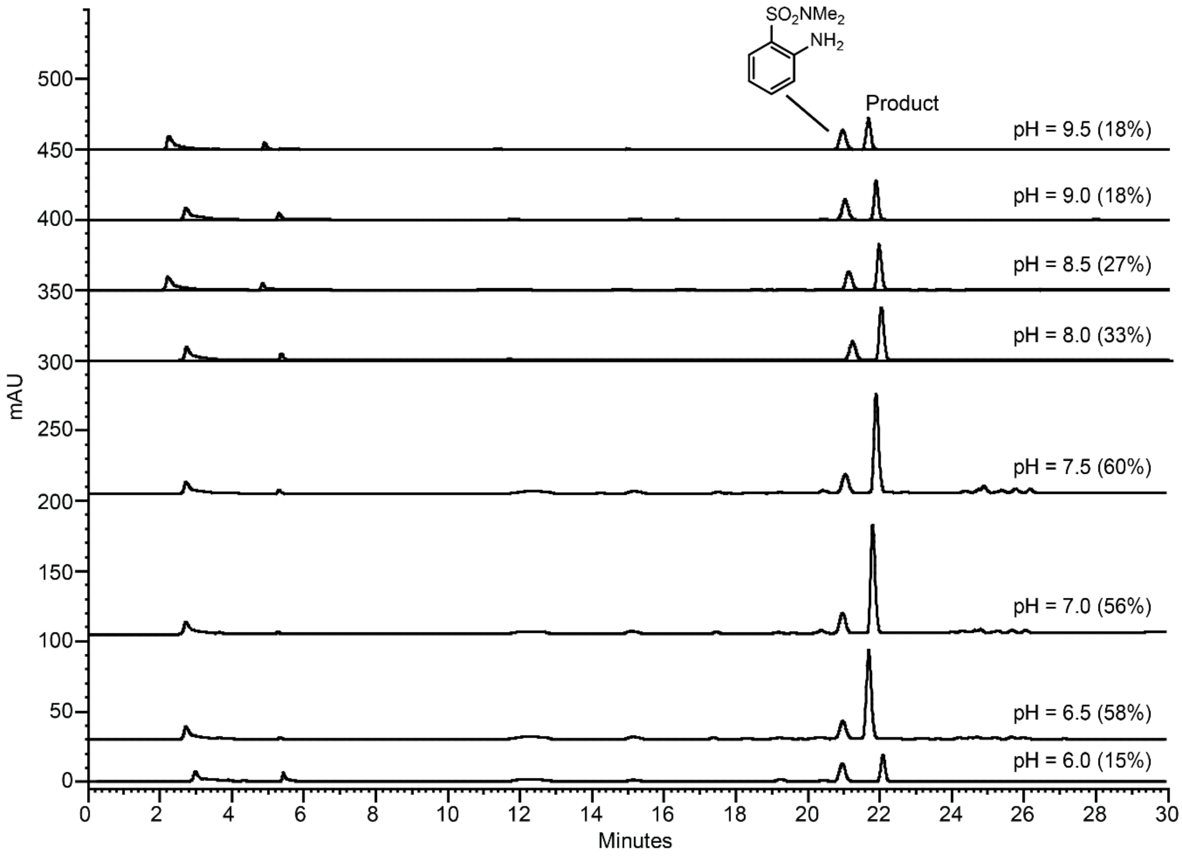

Figure S27. RP-HPLC trace of the reaction of angiotensin IV (**5**) at various pH at 310 nm. Ramp: 20-60% MeCN 28 mins.

Copper(II) Concentration

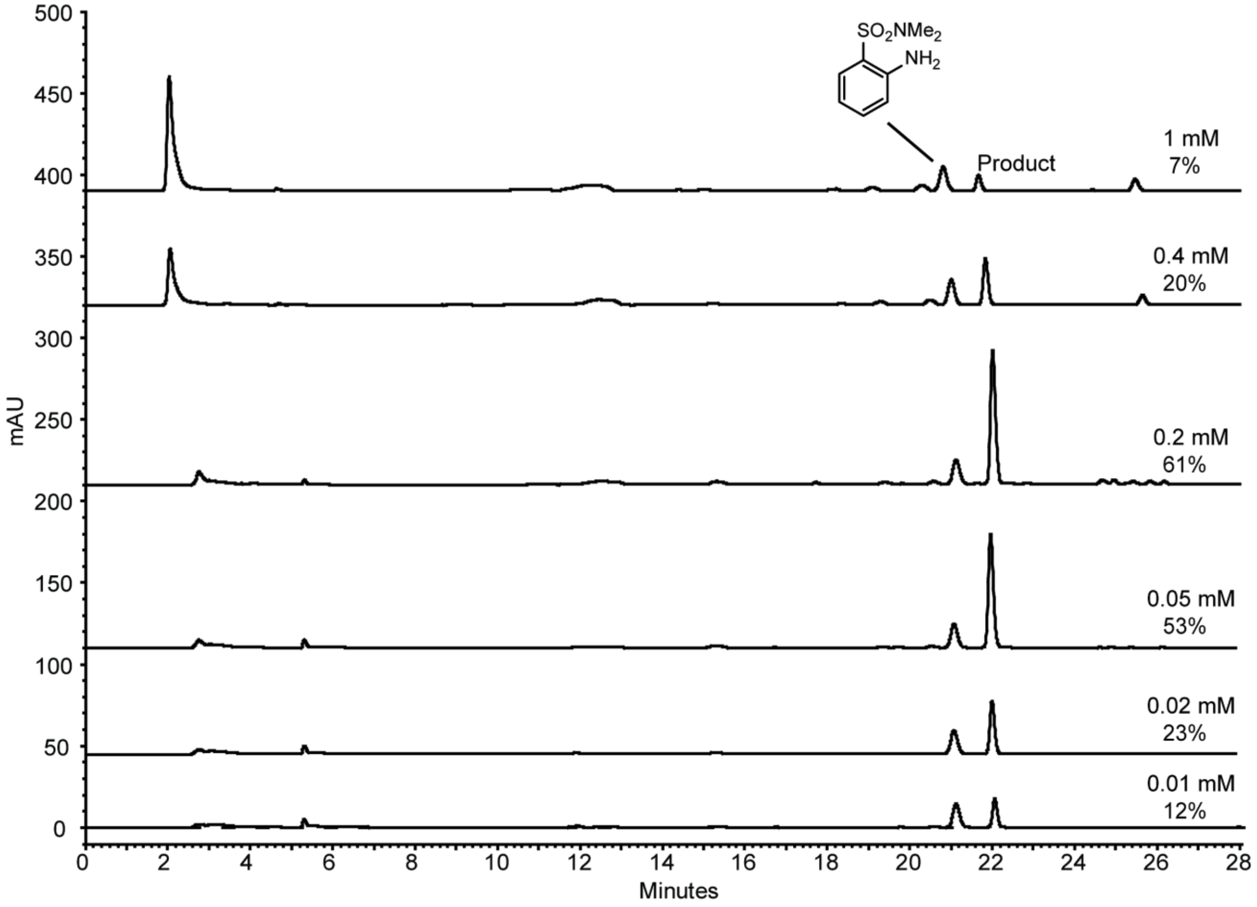

Figure S28. RP-HPLC trace of the reaction of angiotensin IV (**5**) at various copper(II) concentrations at 310 nm. Ramp: 20-60% MeCN 28 mins.

Boronic Acid Concentration

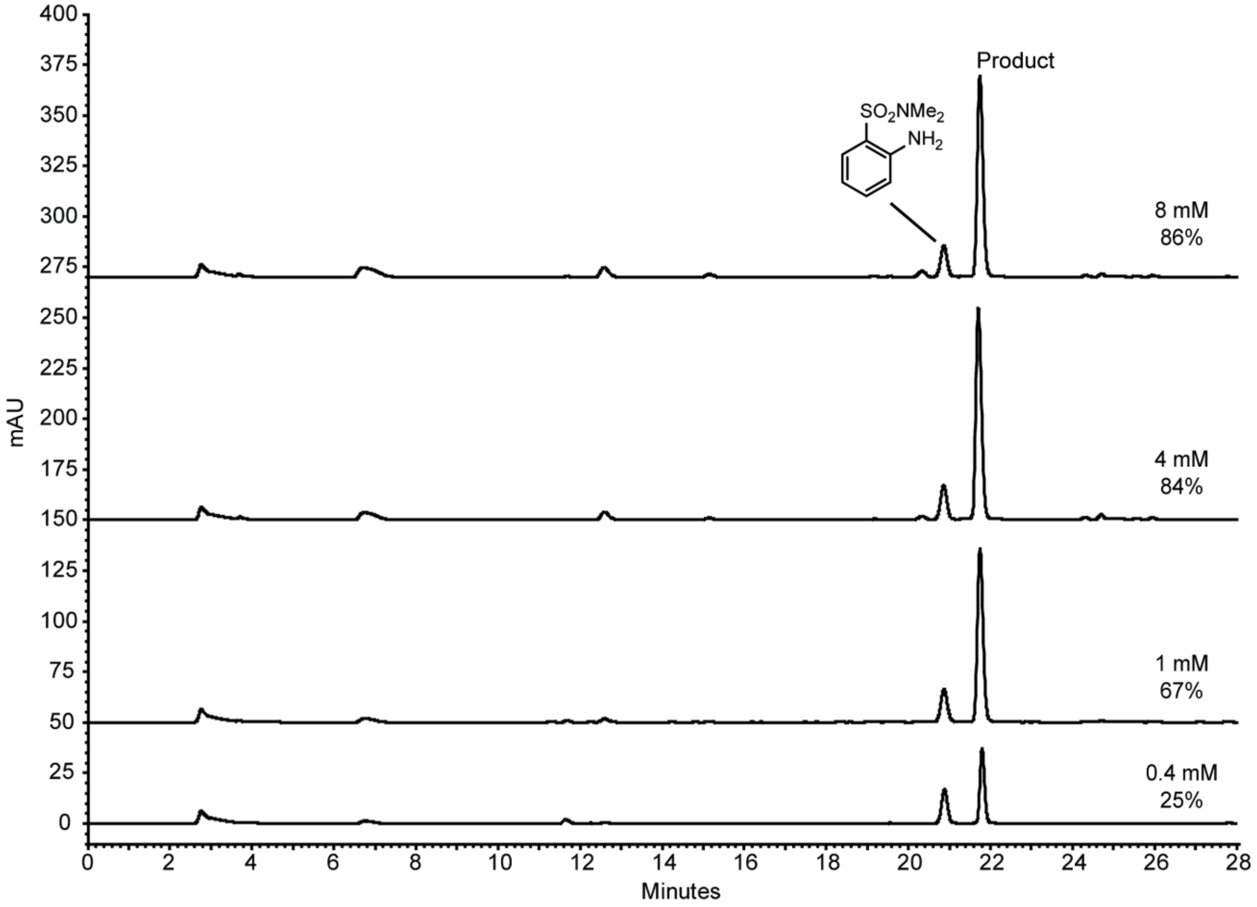

Figure S29. RP-HPLC trace of the reaction of angiotensin IV (**5**) at various boronic acid concentrations at 310 nm. Ramp: 20-60% MeCN 28 mins.

Buffer Identity

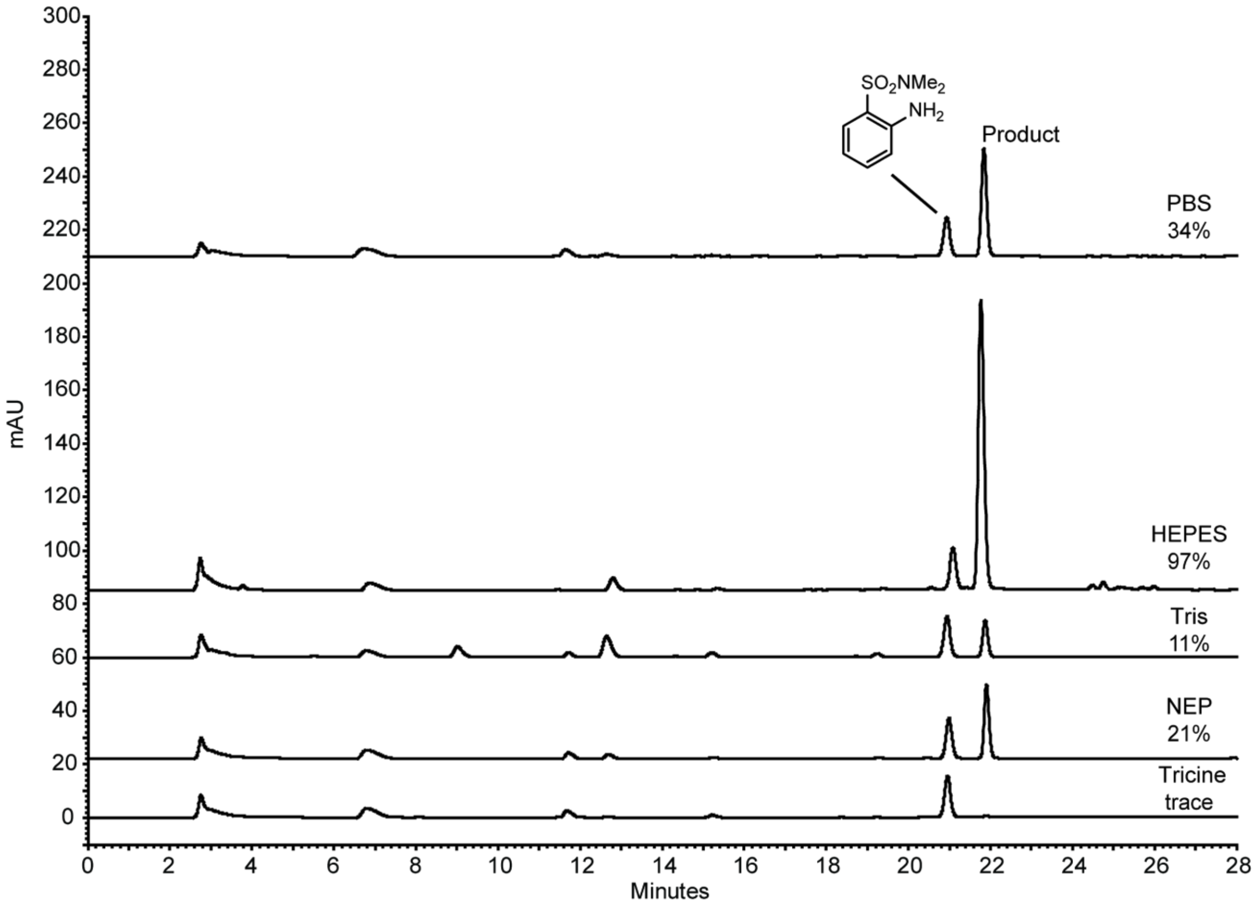

Figure S30. RP-HPLC trace of the reaction of angiotensin IV (**5**) with various buffers at 310 nm. Ramp: 20-60% MeCN 28 mins.

Cosolvent Identity

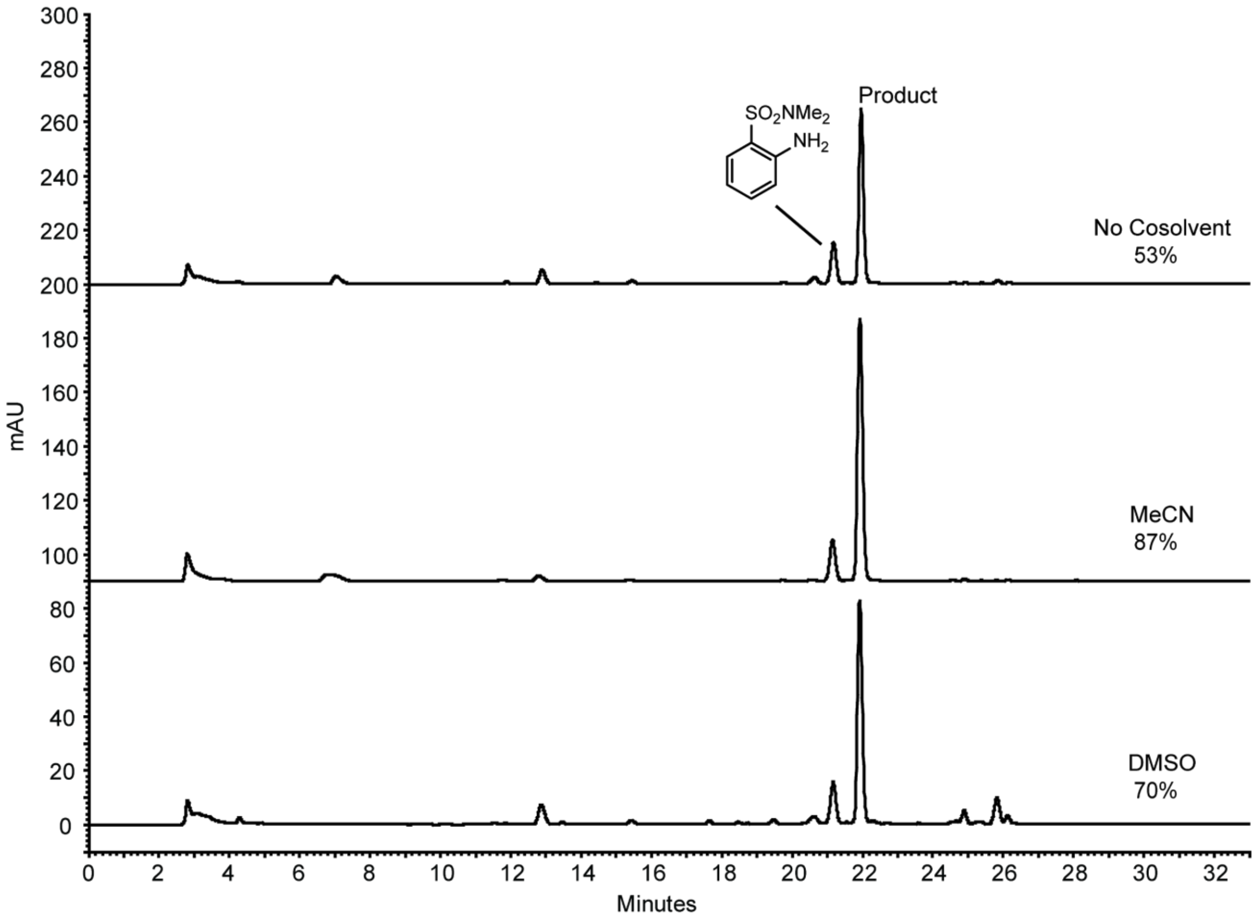

Figure S31. RP-HPLC trace of the reaction of angiotensin IV (**5**) using various cosolvents at 310 nm. Ramp: 20-60% MeCN 28 mins.

Percent Cosolvent

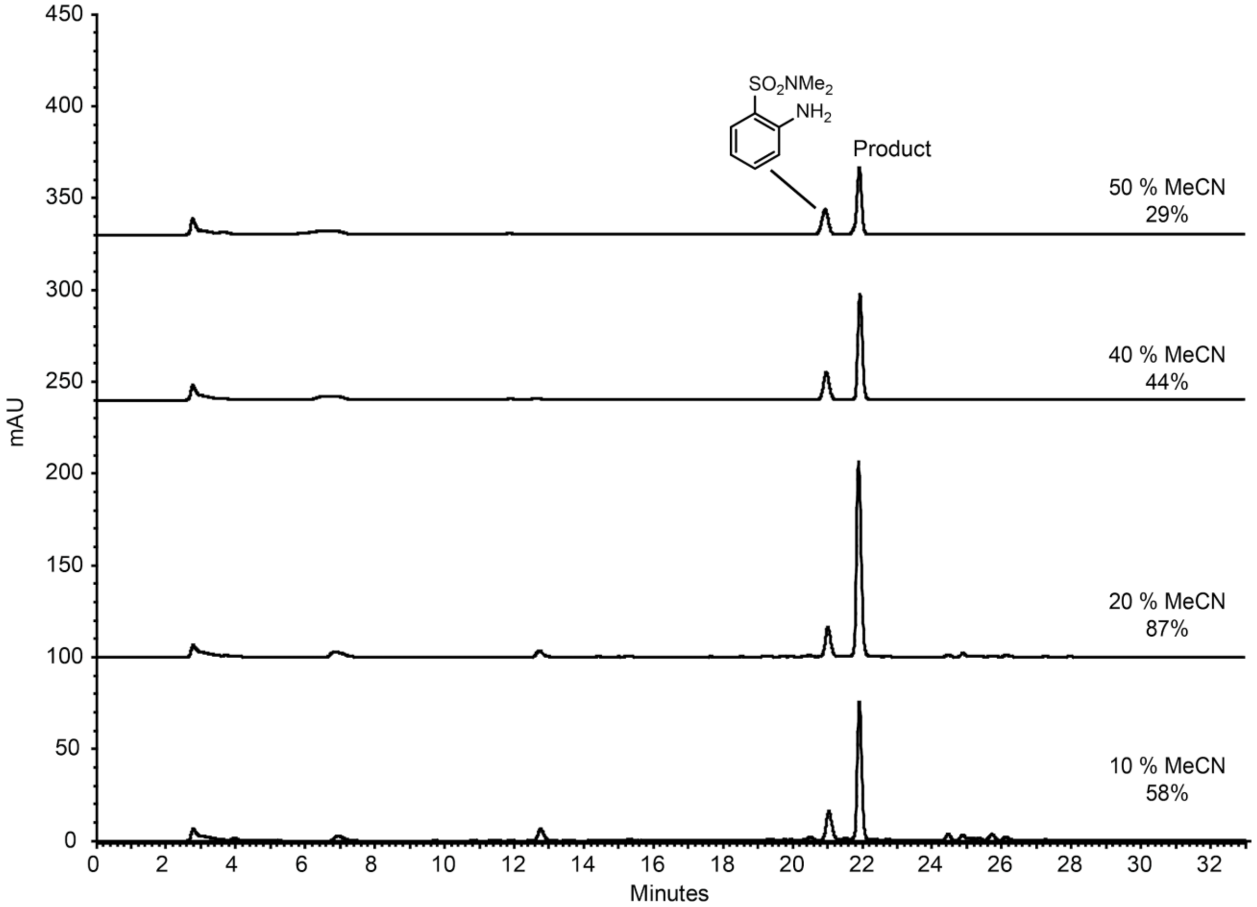

Figure S32. RP-HPLC trace of the reaction of angiotensin IV (**5**) using various amounts of acetonitrile at 310 nm. Ramp: 20-60% MeCN 28 mins.

For Table 2

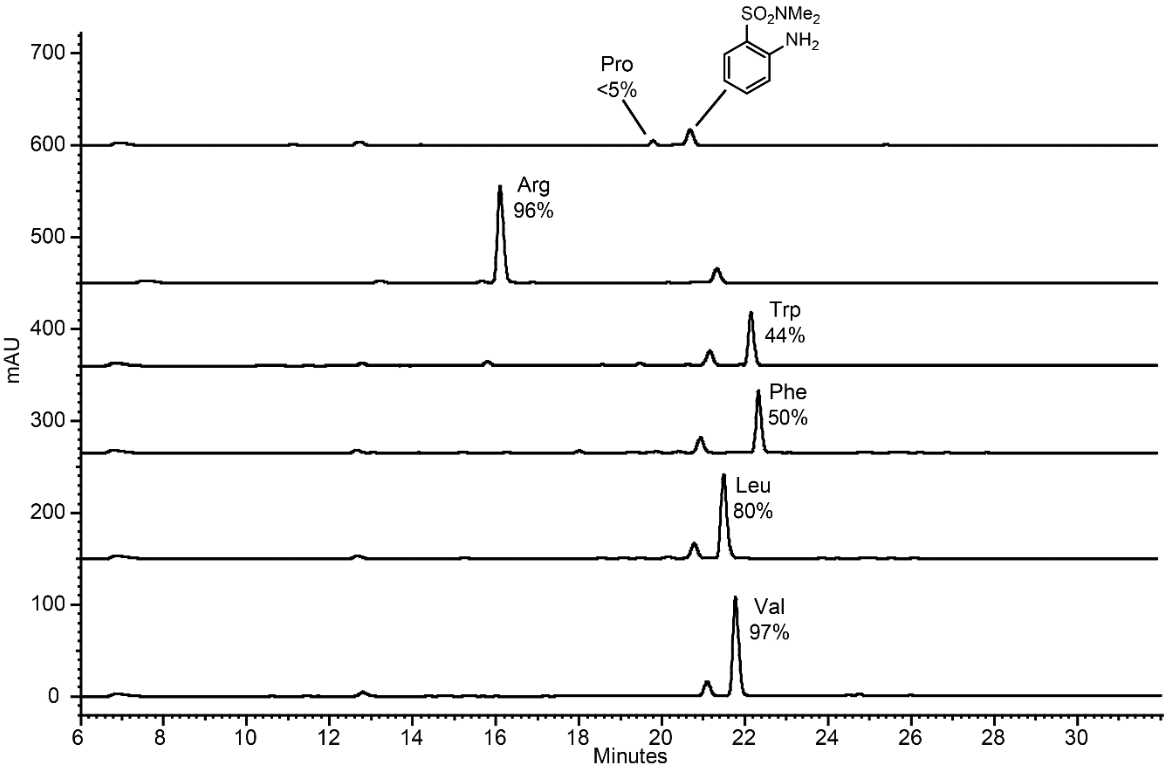

Figure S33. RP-HPLC trace of the reaction of angiotensin IV N-terminal mutants from Table 2 at 310 nm. Ramp: 20-60% MeCN 28 mins.

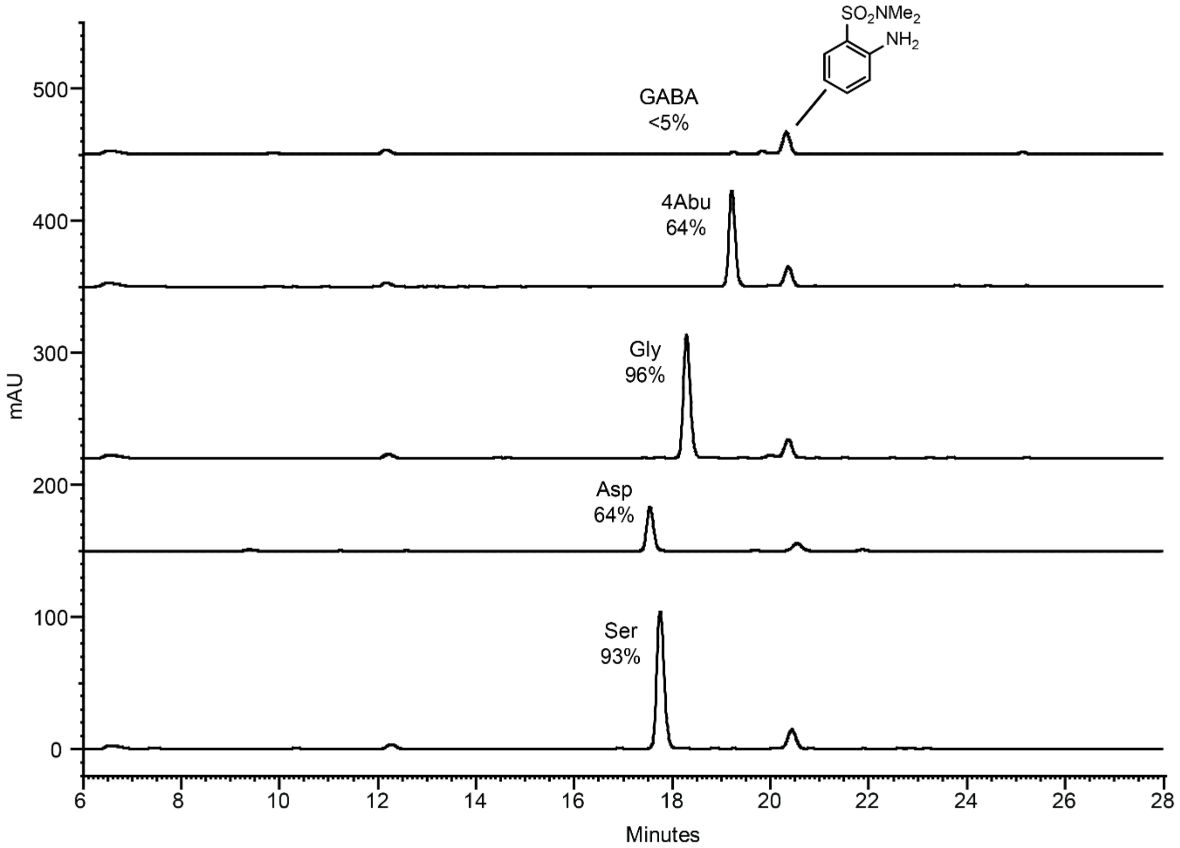

Figure S34. RP-HPLC trace of the reaction of angiotensin IV N-terminal mutants from Table 2 at 310 nm. Ramp: 20-60% MeCN 28 mins.

For Table 3

Spantide I (H-RPKPQQWFWLL-NH_2) (**16**)

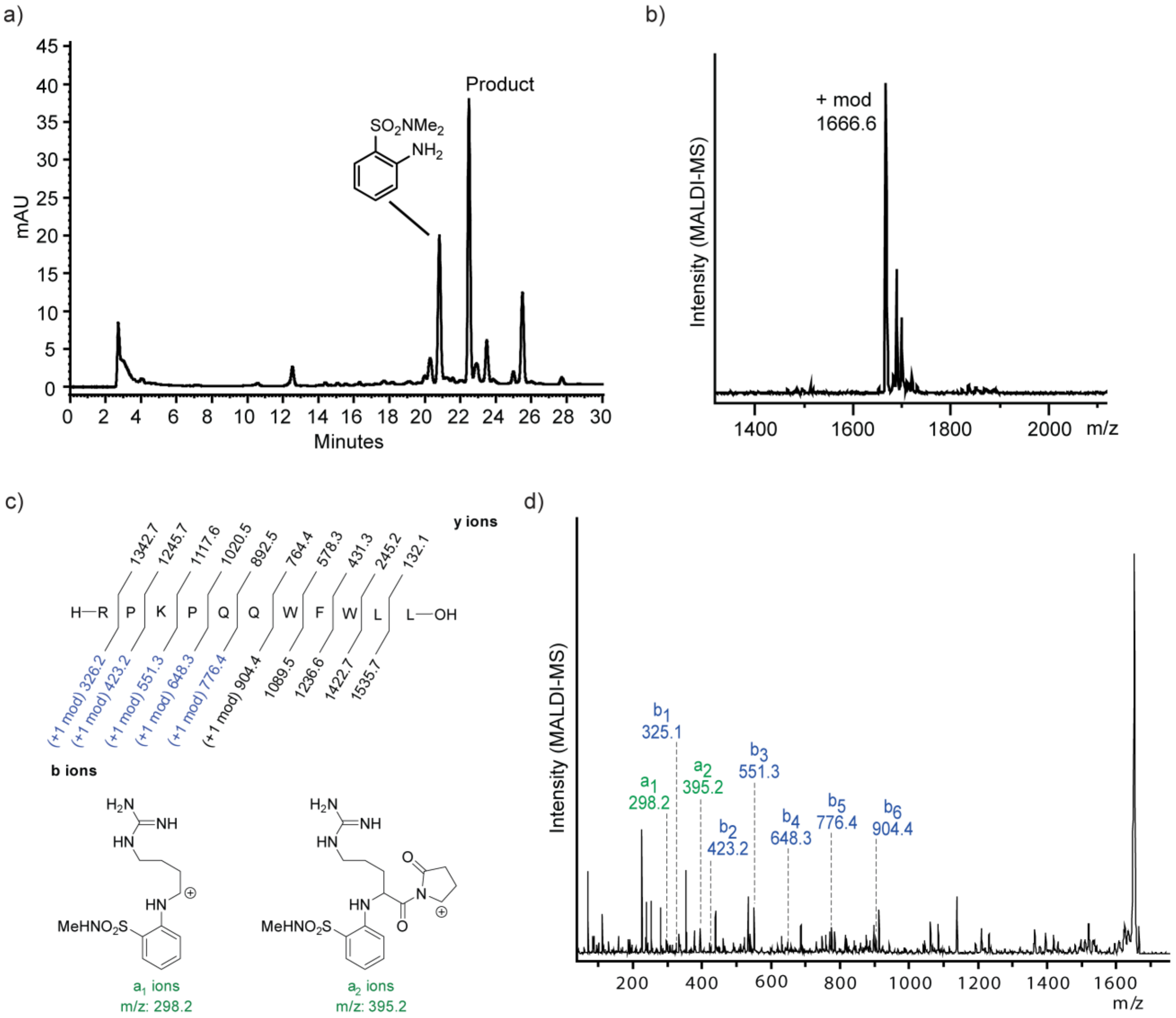

Figure S35. (a) HPLC trace for the modification of peptide **16** at 310 nm. Ramp: 20-60% MeCN 28 mins. (b) MALDI-MS of the crude reaction mixture of peptide **16**. Modified product was noted at 1666.6 m/z. Sinapic acid was used as a matrix. (c) Sequence and fragmentation ladder of peptide **16** modified with boronic acid **1a**. Observed *b* and *y* ions are indicated. (d) MALDI-MS/MS spectrum of peptide **16** modified with boronic acid **1a** modified at the N-terminus. The selected peaks are labeled.

MOG Peptide (H-MEVGWYRSPFSRVVHLYRNGK-OH) (**18**)

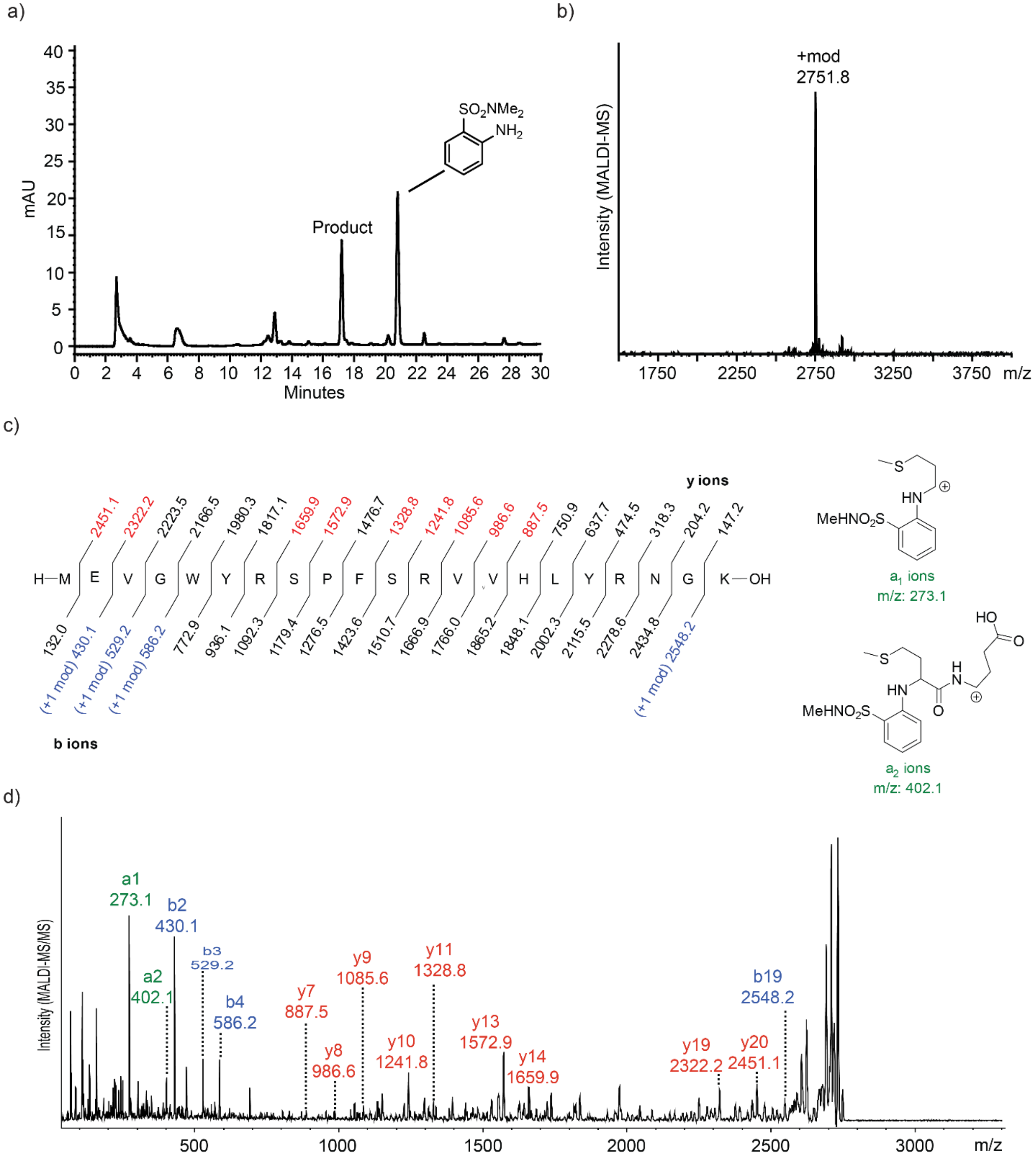

Figure S36. (a) HPLC trace for the modification of peptide **18** at 310 nm. Ramp: 20-60% MeCN 28 mins. (b) MALDI-MS of the crude reaction mixture of peptide **18** modified with boronic acid **1a**. Modified product was noted at 2751.8 m/z. Sinapic acid was used as a matrix. (c) Sequence and fragmentation ladder of peptide **18** modified with boronic acid **1a**. Observed *b* and *y* ions are indicated. (d) MALDI-MS/MS spectrum of peptide **18** modified with boronic acid **1a** modified at the N-terminus. The selected peaks are labeled.

Angiotensin I (H-DRVYIHPFHL-OH) (**17**)

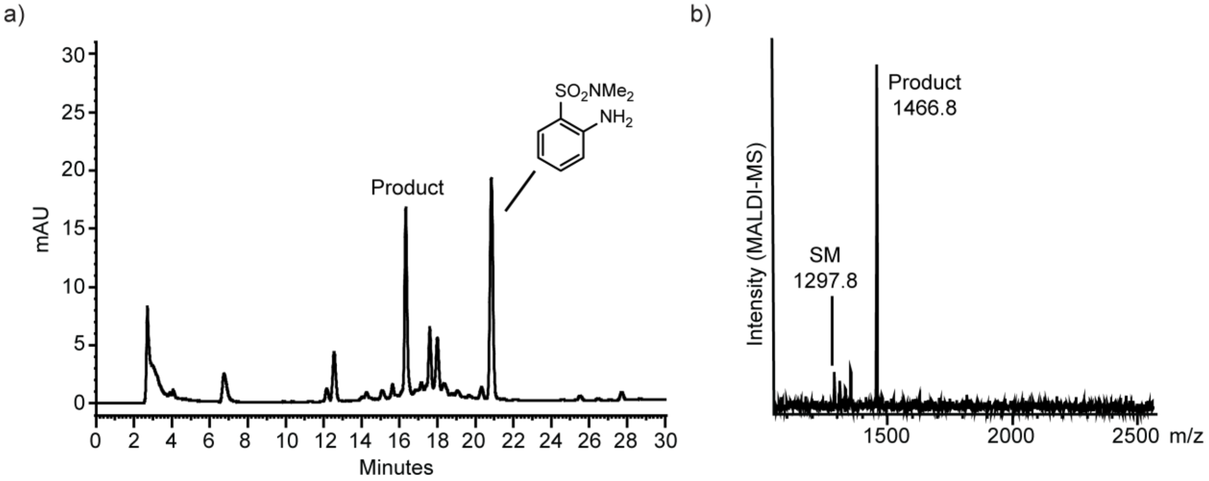

Figure S37. (a) HPLC trace for the modification of peptide **17** with boronic acid **1a** at 310 nm. Ramp: 20-60% MeCN 28 mins. (b) MALDI-MS of the crude reaction mixture of peptide **17**. Modified product was noted at 1466.8 m/z. Sinapic acid was used as a matrix.

UV-visible Absorption Spectroscopy

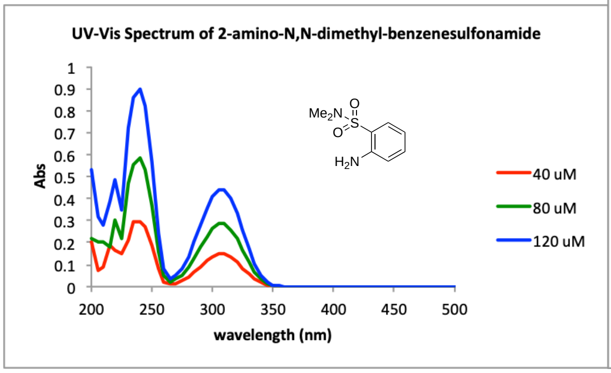

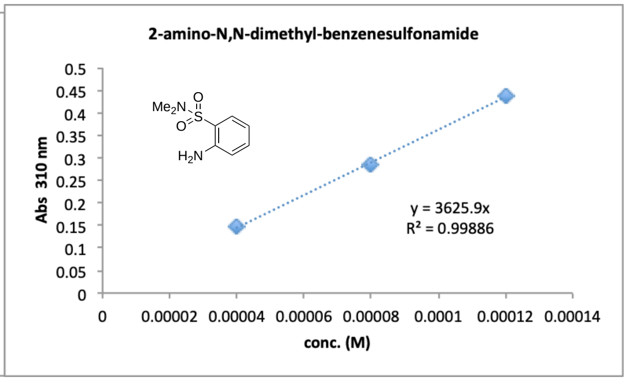

conc. (M)	0.00004	0.00008	0.00012
Abs 310 nm	0.146621138	0.284186631	0.438505858
ε_{310} ($M^{-1}cm^{-1}$)	3600		

Figure S38. UV-Vis spectrum and calculation of the extinction coefficient for 2-amino-N,N-dimethyl-benzenesulfonamide (**19**)

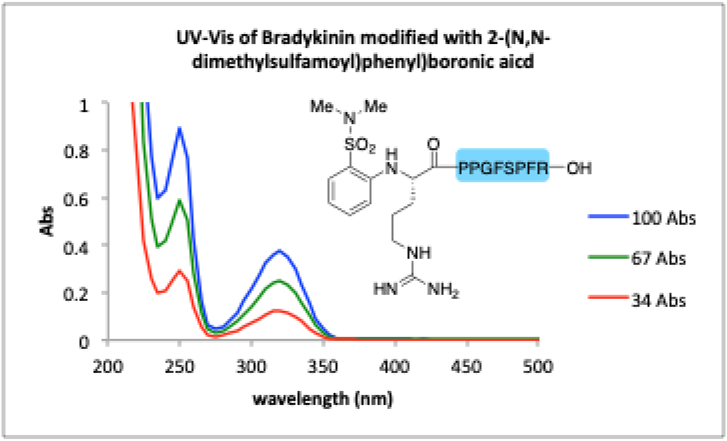

ε_{310} (M^{-1} cm^{-1})	3300

Figure S39. UV-Vis spectrum and calculated extinction coefficient for arylated bradykinin (**2b**)

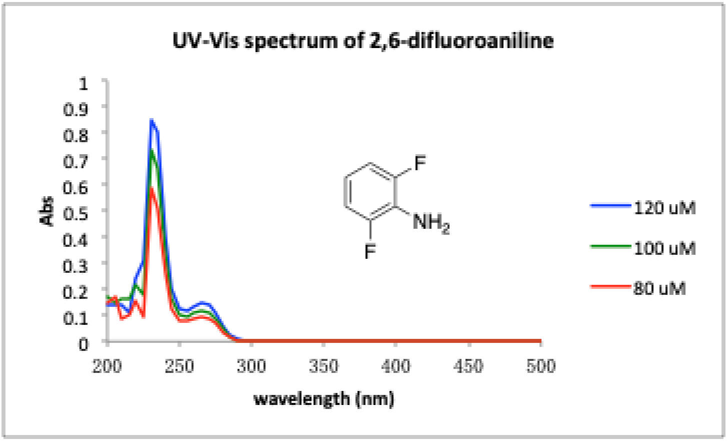

ε_{240} (M^{-1} cm^{-1})	3400

Figure S40. UV-Vis spectrum and calculated extinction coefficient for 2,6-difluoroaniline (**S1**). Internal standard for modified peptide **2h**.

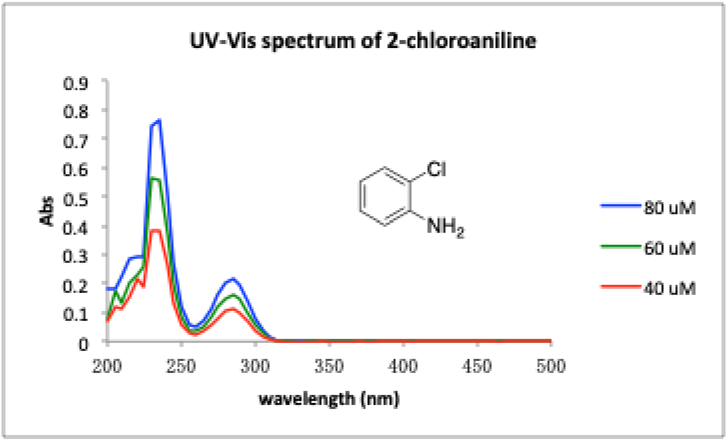

ε_{240} (M^{-1} cm^{-1})	6400

Figure S41. UV-Vis spectrum and calculated extinction coefficient for 2-chloroaniline (**S2**). Internal standard for modifed peptide **2f**.

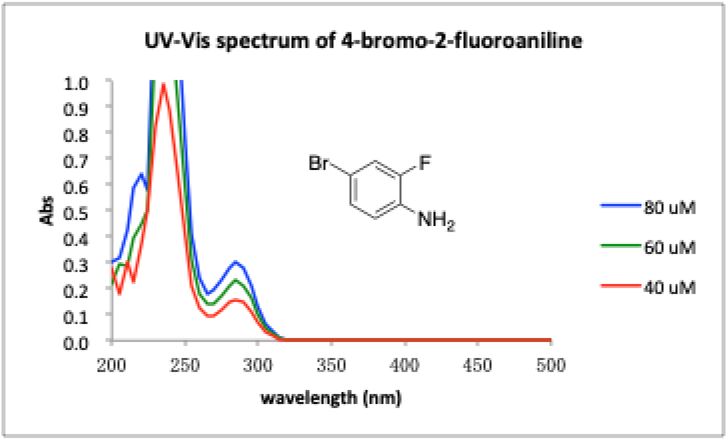

ε_{290} (M^{-1} cm^{-1})	3500

Figure S42. UV-Vis spectrum and calculated extinction coefficient for 4-bromo-2-fluoroaniline (**S3**). Internal standard for modified peptide **2i**.

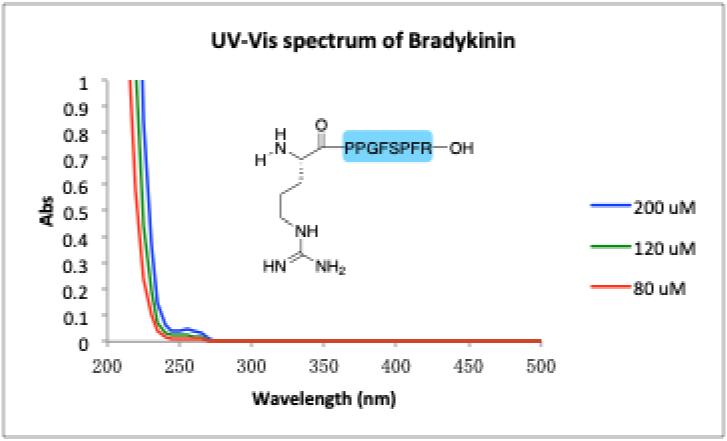

ε_{310} (M^{-1} cm^{-1})	0
ε_{290} (M^{-1} cm^{-1})	0
ε_{240} (M^{-1} cm^{-1})	290

Figure S43. UV-Vis spectrum and calculated extinction coefficient for bradykinin (**2**)

Characterization- HPLC, ESI-MS, ^{1}H and ^{13}C NMR

HPLC and ESI-MS of Synthesized peptides from Table 2

H-WYIHPF-NH$_2$ (8)

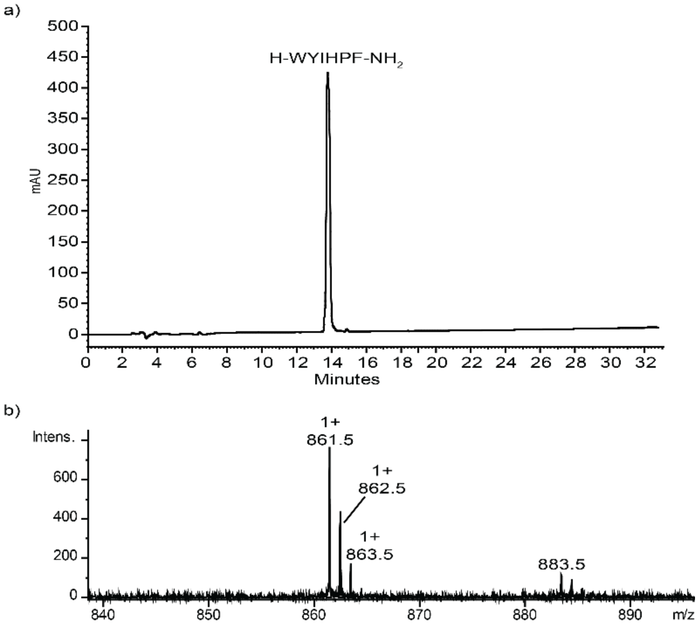

Figure S44. (a) HPLC spectrum for the purified peptide **8**. Ramp: 20-60% MeCN 28 mins. (b) ESI-MS spectrum of peptide **8**. m/z 861.5 and 883.5 correspond to $[M+H]^+$ and $[M+Na]^+$, respectively.

H-GYIHPF-NH_2 (13)

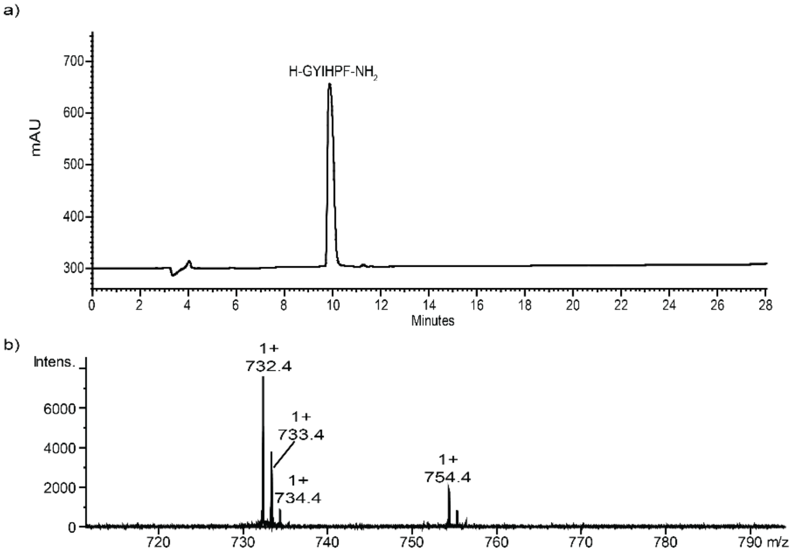

Figure S45. (a) HPLC spectrum for the purified peptide **13**. Ramp: 20-60% MeCN 28 mins. (b) ESI-MS spectrum of peptide **13**. m/z 732.4 and 754.4 correspond to $[M+H]^+$ and $[M+Na]^+$, respectively.

H-LYIHPF-NH_2 (6)

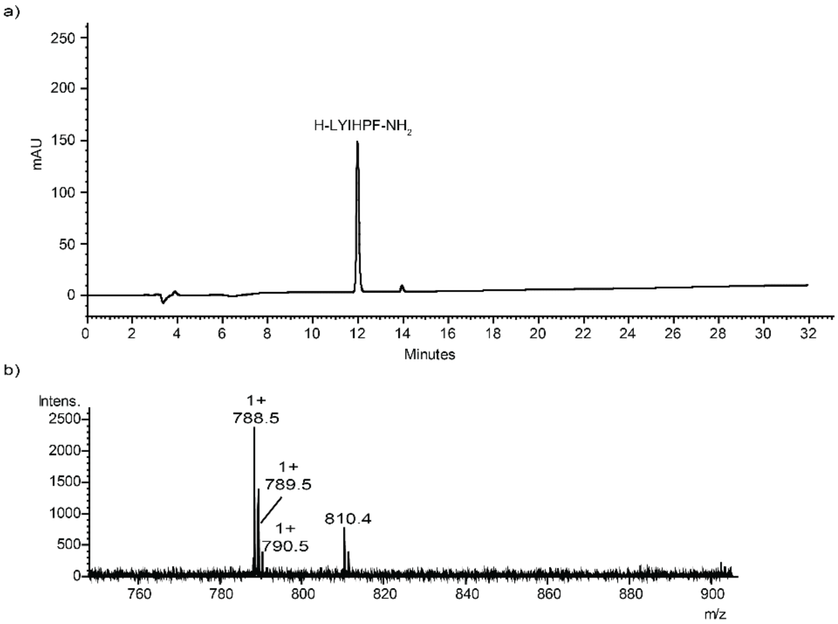

Figure S46. (a) HPLC spectrum for the purified peptide **6**. Ramp: 20-60% MeCN 28 mins. (b) ESI-MS spectrum of peptide **6**. m/z 788.5 and 810.0 correspond to $[M+H]^+$ and $[M+Na]^+$, respectively.

H-FYIHPF-NH_2 (7)

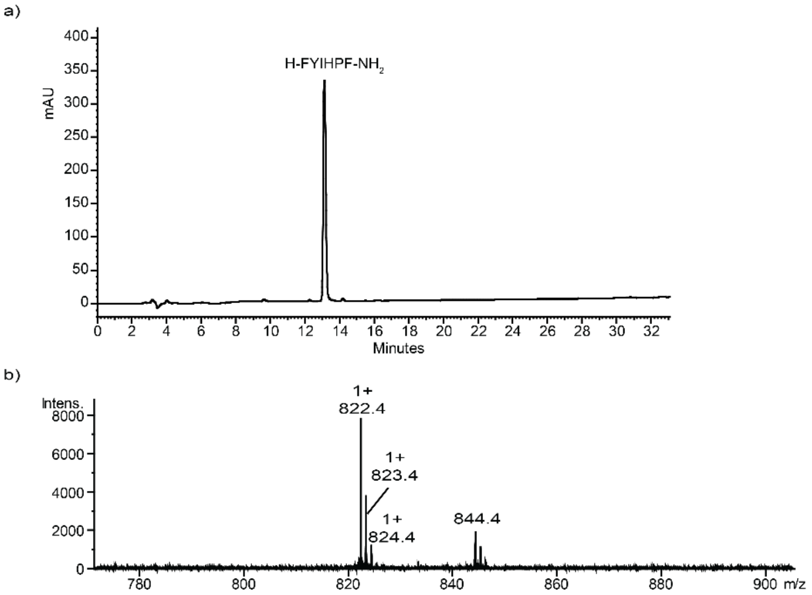

Figure S47. (a) HPLC spectrum for the purified peptide **7**. Ramp: 20-60% MeCN 28 mins. (b) ESI-MS spectrum of peptide **7**. m/z 822.4 and 844.4 correspond to $[M+H]^+$ and $[M+Na]^+$, respectively.

H-RYIHPF-NH_2 (9)

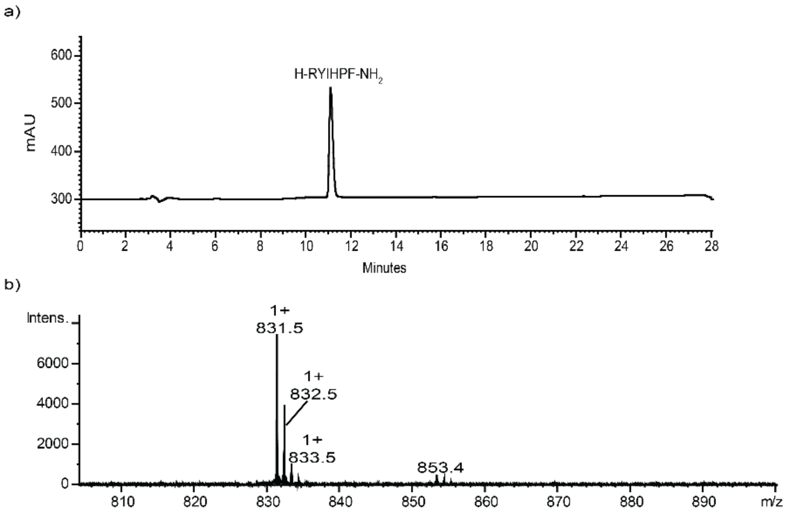

Figure S48. (a) HPLC spectrum for the purified peptide **9**. Ramp: 20-60% MeCN 28 mins. (b) ESI-MS spectrum of peptide **9**. m/z 831.5 and 853.4 correspond to $[M+H]^+$ and $[M+Na]^+$, respectively.

H-DYIHPF-NH$_2$ (12)

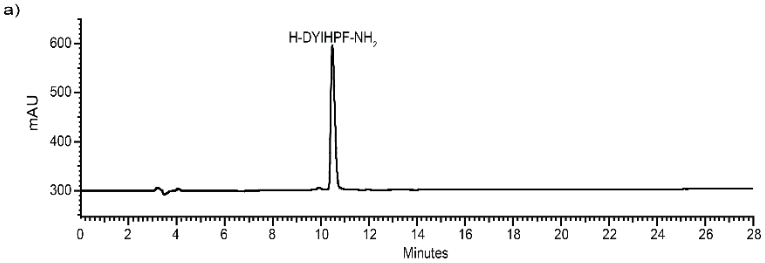

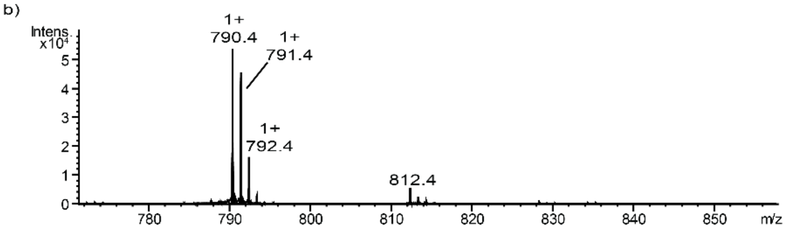

Figure S49. (a) HPLC spectrum for the purified peptide **12**. Ramp: 20-60% MeCN 28 mins. (b) ESI-MS spectrum of peptide **12**. m/z 790.4 and 812.4 correspond to $[M+H]^+$ and $[M+Na]^+$, respectively.

H-SYIHPF-NH$_2$ (11)

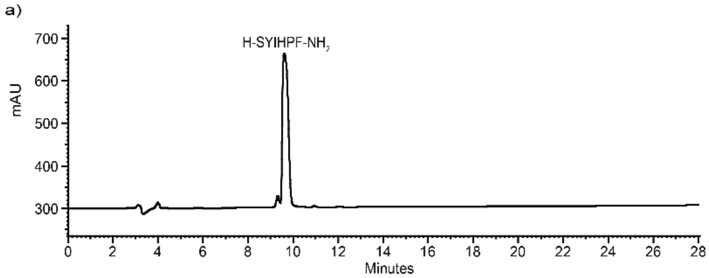

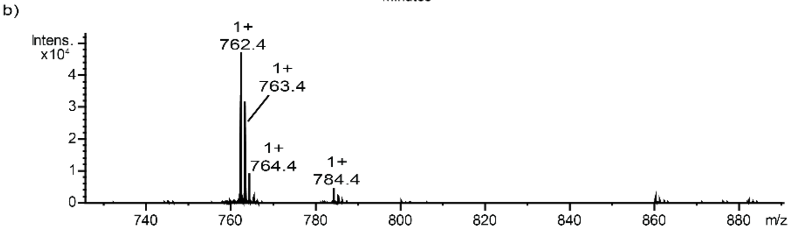

Figure S50. (a) HPLC spectrum for the purified peptide **11**. Ramp: 20-60% MeCN 28 mins. (b) ESI-MS spectrum of peptide **11**. m/z 762.4 and 784.4 correspond to $[M+H]^+$ and $[M+Na]^+$, respectively.

H-PYIHPF-NH$_2$ (10)

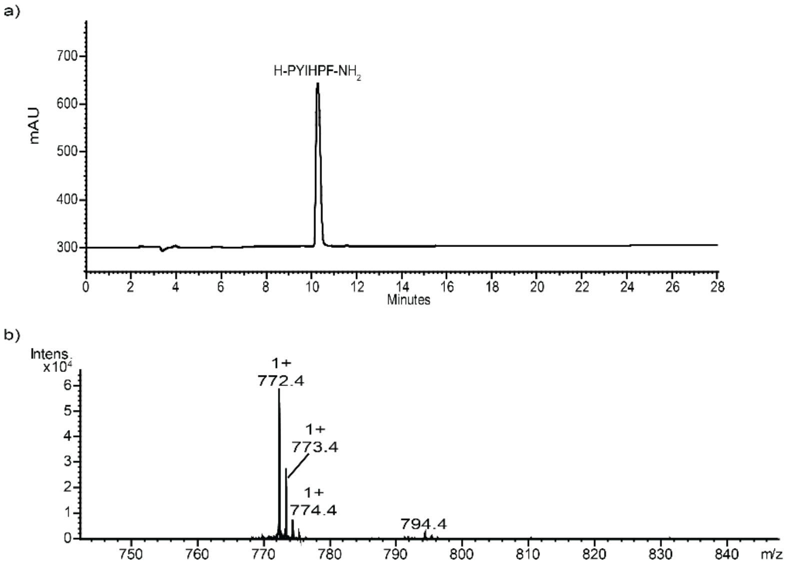

Figure S51. (a) HPLC spectrum for the purified peptide **10**. Ramp: 20-60% MeCN 28 mins. (b) ESI-MS spectrum of peptide **10**. m/z 772.4 and 794.4 correspond to $[M+H]^+$ and $[M+Na]^+$, respectively.

H-bAYIHPF-NH$_2$ (14)

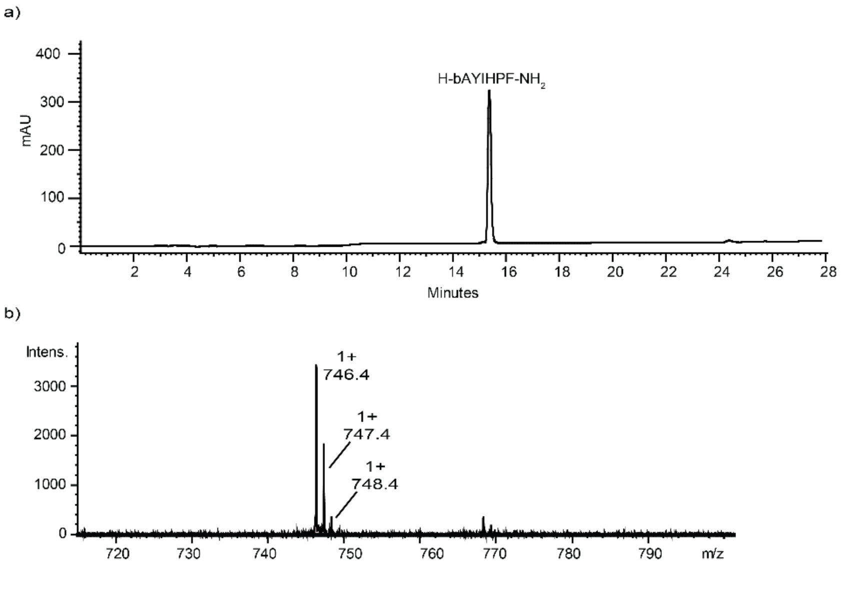

Figure S52. (a) HPLC spectrum for the purified peptide **14**. Ramp: 20-60% MeCN 28 mins. (b) ESI-MS spectrum of peptide **14**. m/z 746.4 corresponds to $[M+H]^+$.

H-(4Abu)YIHPF-NH_2 (15)

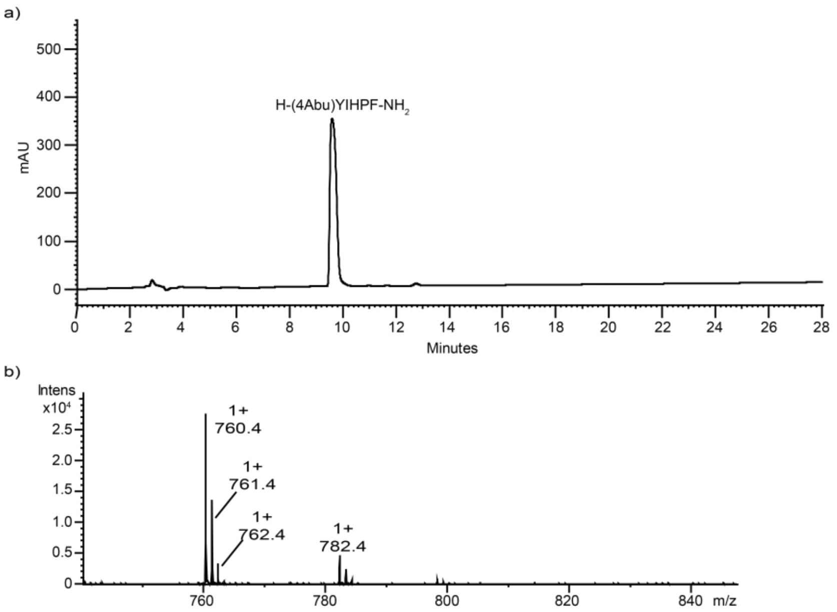

Figure S53. (a) HPLC spectrum for the purified peptide **15**. Ramp: 20-60% MeCN 28 mins. (b) ESI-MS spectrum of peptide **15**. m/z 760.4 and 782.4 correspond to $[M+H]^+$ and $[M+Na]^+$, respectively.

ESI-MS of Synthesized Compounds

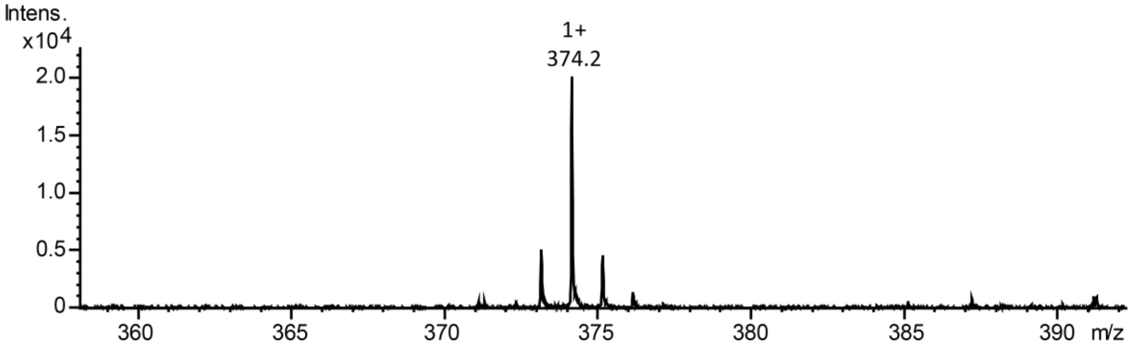

Figure S54. *N*-benzyl-2-(4,4,5,5-tetramethyl-1,3,2-dioxaborolan-2-yl)benzenesulfonamide (**S5**) (positive mode). m/z 374.2 corresponds to $[M+H]^+$.

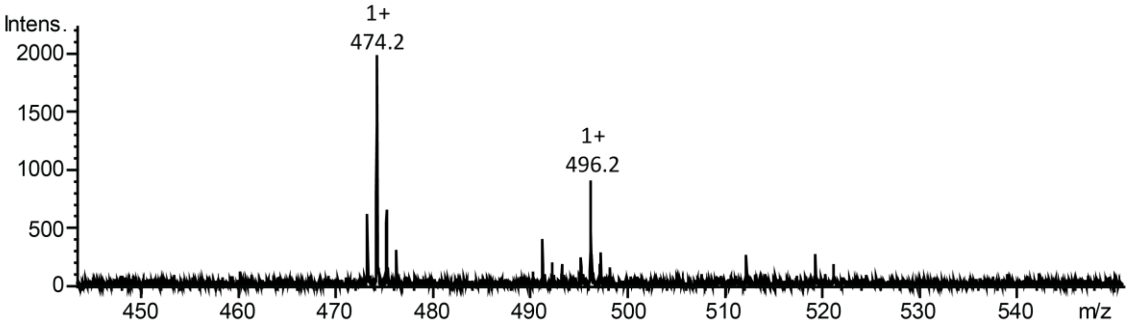

Figure S55. *tert*-butyl benzyl((2-(4,4,5,5-tetramethyl-1,3,2-dioxaborolan-2-yl)phenyl)sulfonyl)carbamate (**S6**) (positive mode). m/z 474.2 and 496.2 correspond to $[M+H]^+$ and $[M+Na]^+$, respectively.

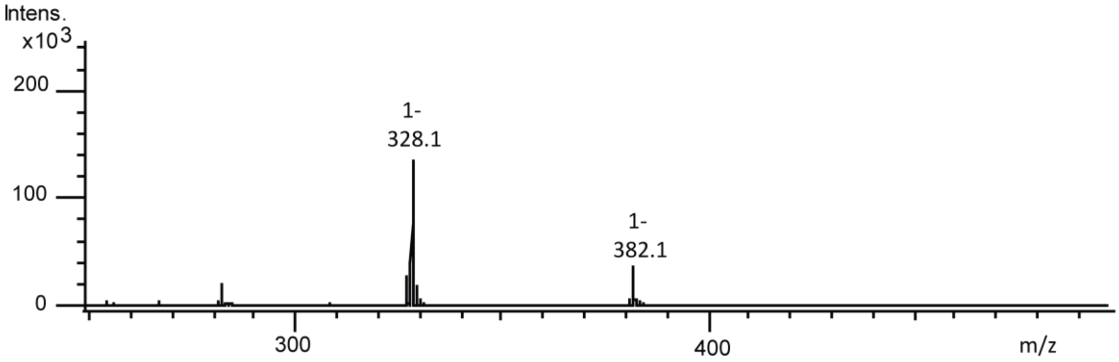

Figure S56. *tert*-butyl ((2-(4,4,5,5-tetramethyl-1,3,2-dioxaborolan-2-yl)phenyl)sulfonyl)carbamate (**S7**) (negative mode). m/z 328.1 and 382.1 correspond to the methanol adduct of $[M-H]^-$ and $[M-H]^-$, respectively.

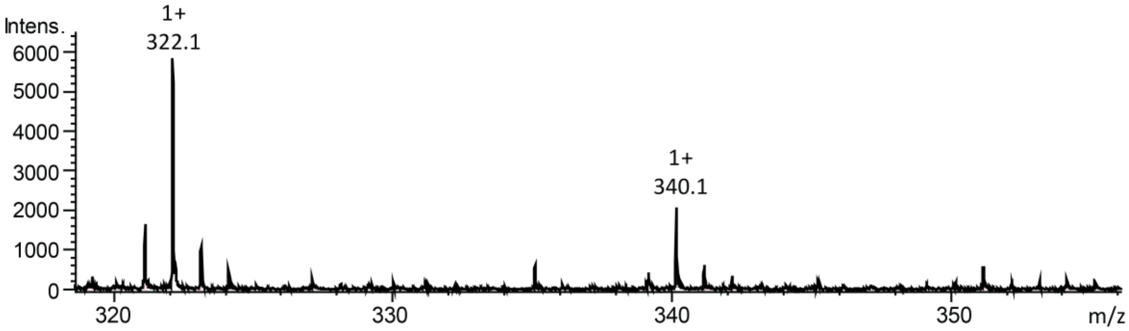

Figure S57. *N*-(prop-2-yn-1-yl)-2-(4,4,5,5-tetramethyl-1,3,2-dioxaborolan-2-yl)benzenesulfonamide (**1o**) (positive mode). m/z 322.1 and 340.1 correspond to $[M+H]^+$ and $[M+Na]^+$, respectively.

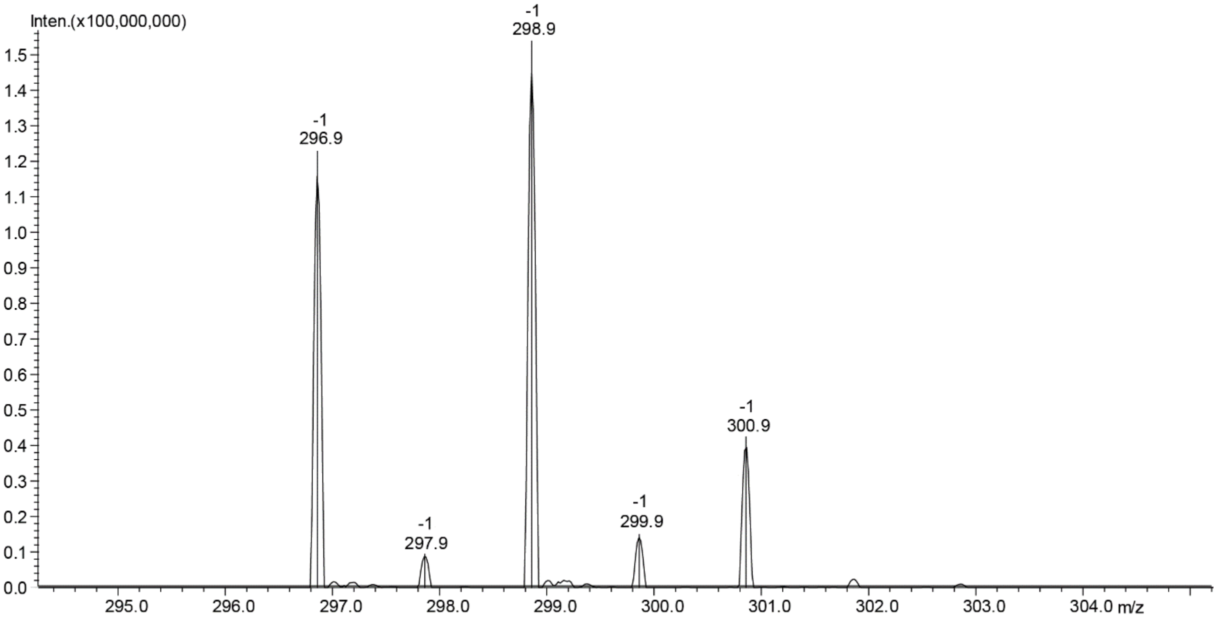

Figure S58. 4-bromo-3-(chlorosulfonyl)benzoic acid (**S8**) (negative mode). m/z 296.9 corresponds to $[M-H]^-$.

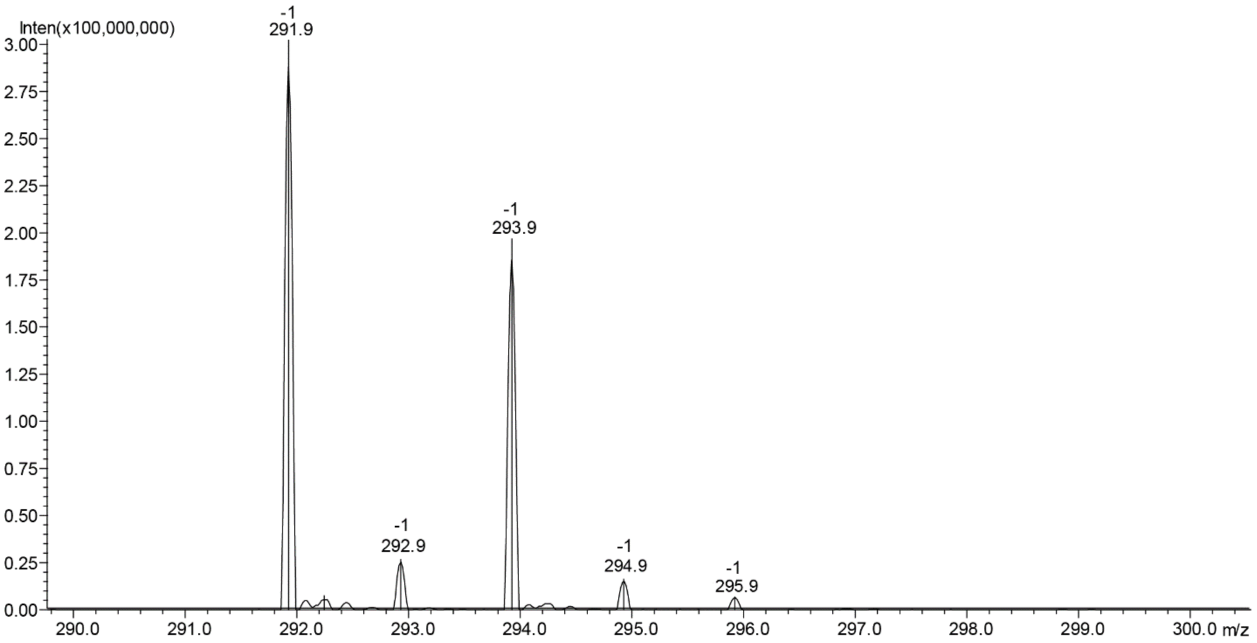

Figure S59. 4-bromo-3-(*N*-methylsulfamoyl)benzoic acid (**S9**) (negative mode). m/z 291.9 corresponds to $[M-H]^-$.

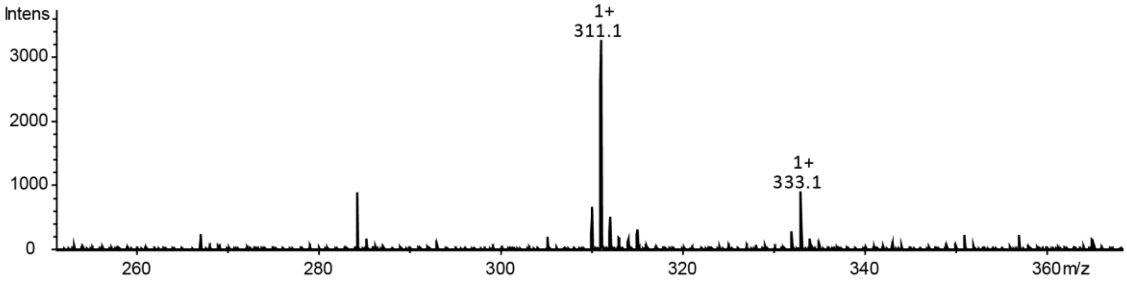

Figure S60. (4-(but-3-yn-1-ylcarbamoyl)-2-(N- methylsulfamoyl)phenyl)boronic acid (**1p**) (positive mode). m/z 311.1 and 333.1 correspond to $[M+H]^+$ and $[M+Na]^+$, respectively.

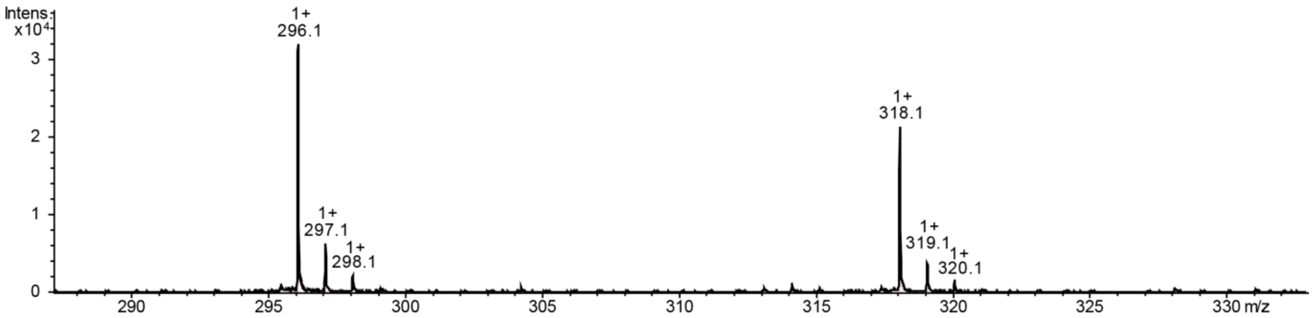

Figure S61. *N*-(but-3-yn-1-yl)-4-(methylamino)-3-(*N*-methylsulfamoyl)benzamide (**S12**) (positive mode). m/z 296.1 and 318.1 correspond to $[M+H]^+$ and $[M+Na]^+$, respectively.

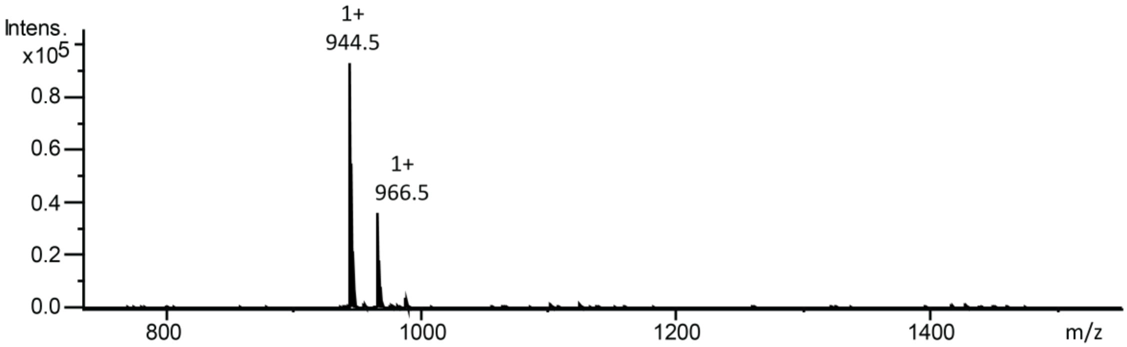

Figure S62. Isolated aryl angiotensin IV (**5a**) (positive mode). m/z 944.5 and 966.5 correspond to $[M+H]^+$ and $[M+Na]^+$, respectively.

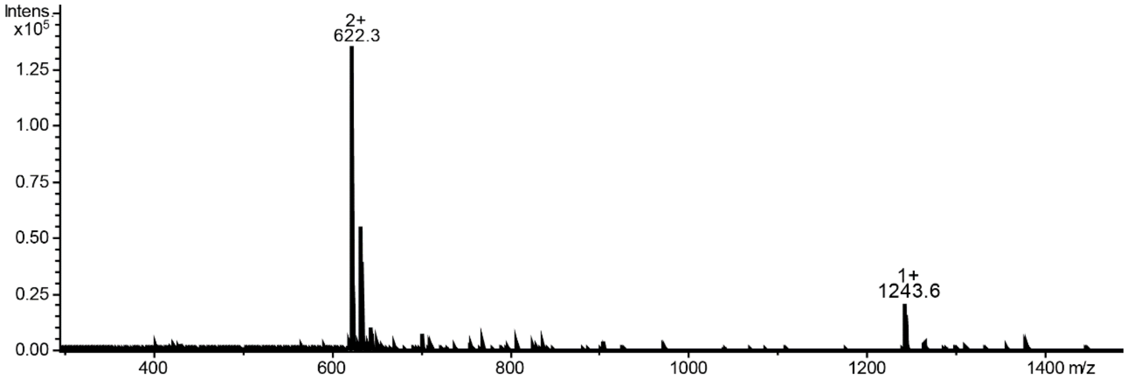

Figure S63. Isolated aryl bradykinin (**2a**) (positive mode). m/z 622.3 and 1243.6 correspond to $[M+2H]^{2+}$ and $[M+H]^+$, respectively.

^{1}H and ^{13}C NMR spectra of synthesized compounds

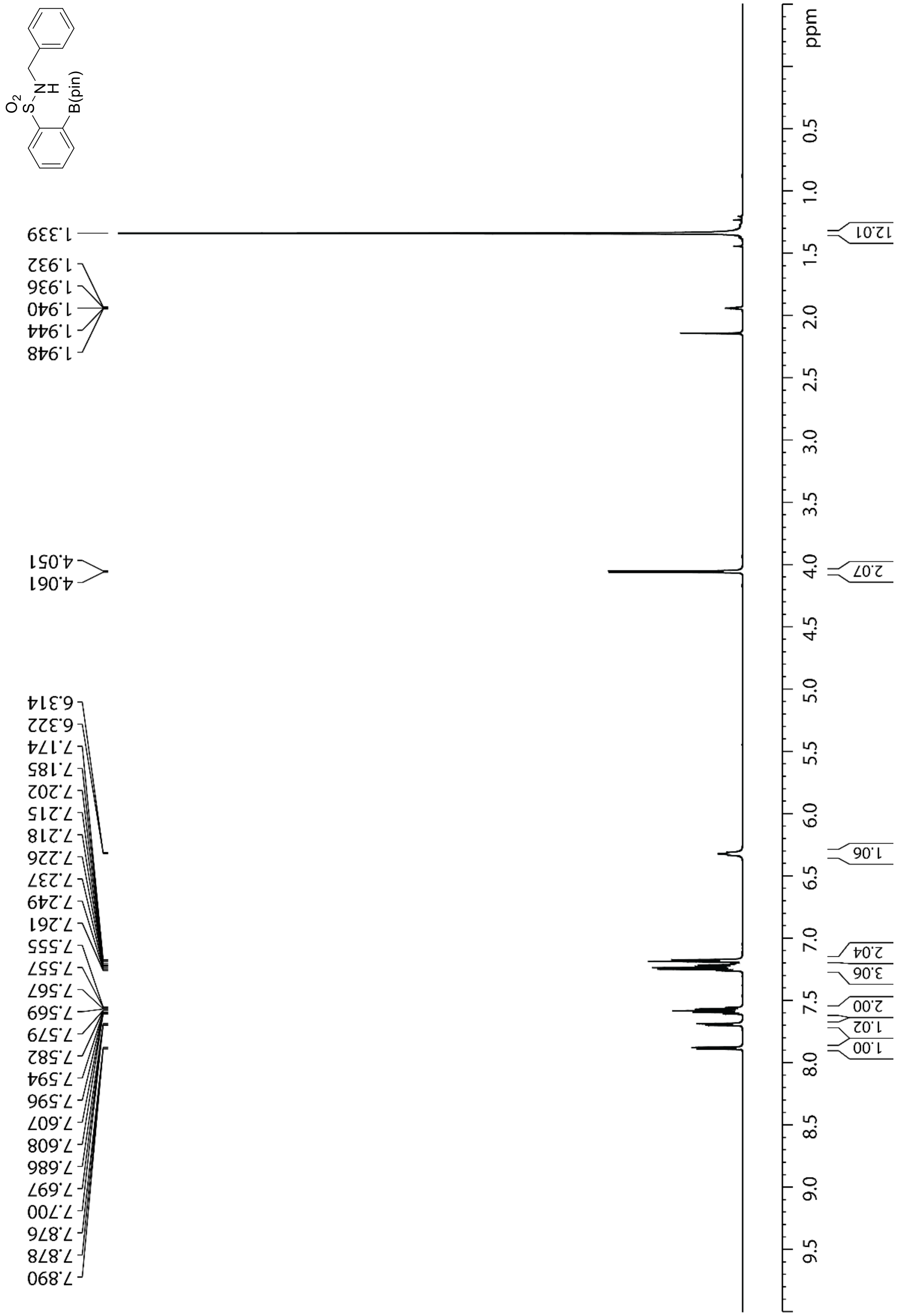

Figure S64. ^{1}H NMR spectrum of *N*-benzyl-2-(4,4,5,5-tetramethyl-1,3,2-dioxaborolan-2-yl)benzenesulfonamide **S5** in CD_3CN

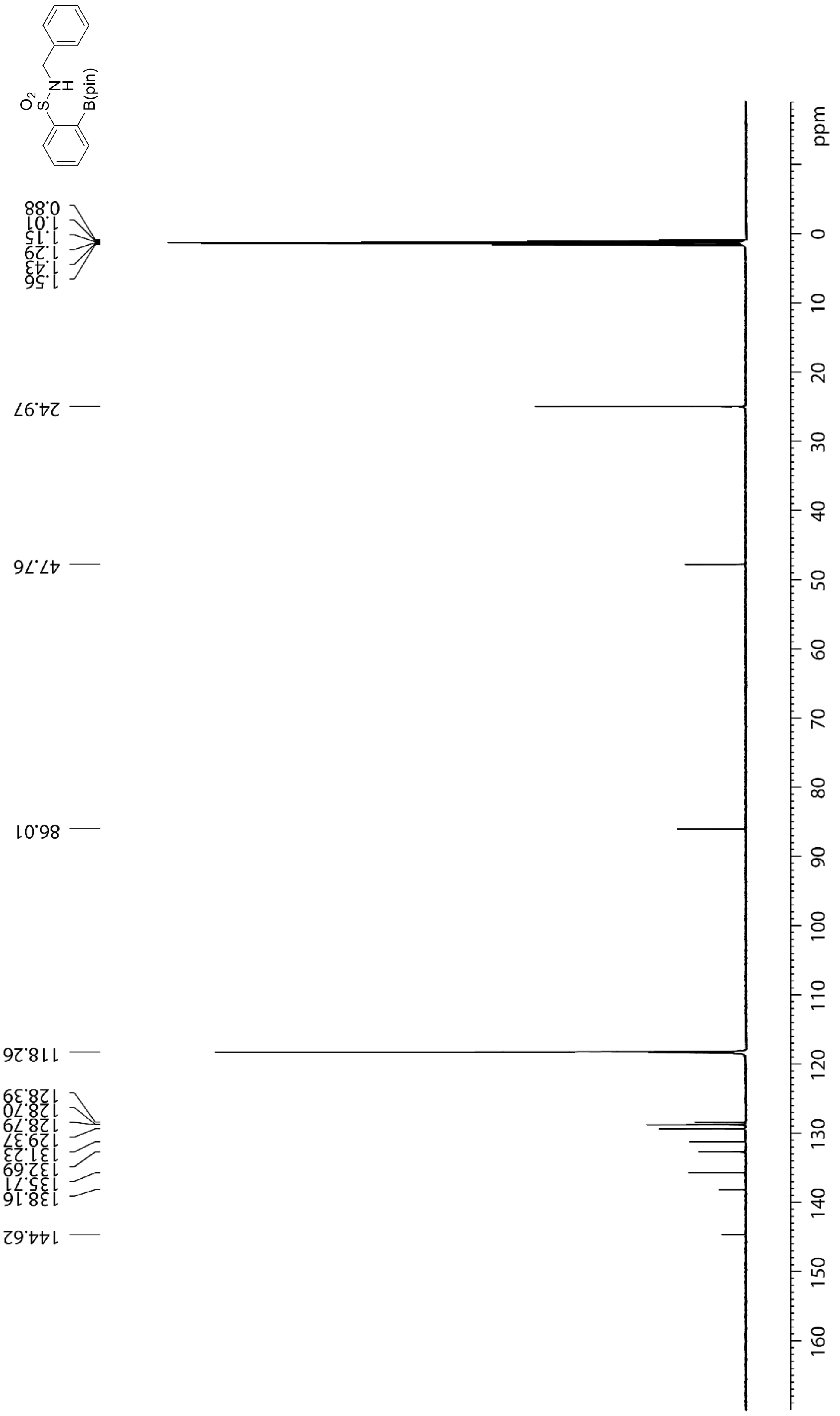

Figure S65. ^{13}C NMR spectrum of *N*-benzyl-2-(4,4,5,5-tetramethyl-1,3,2-dioxaborolan-2-yl)benzenesulfonamide **S5** in CD_3CN

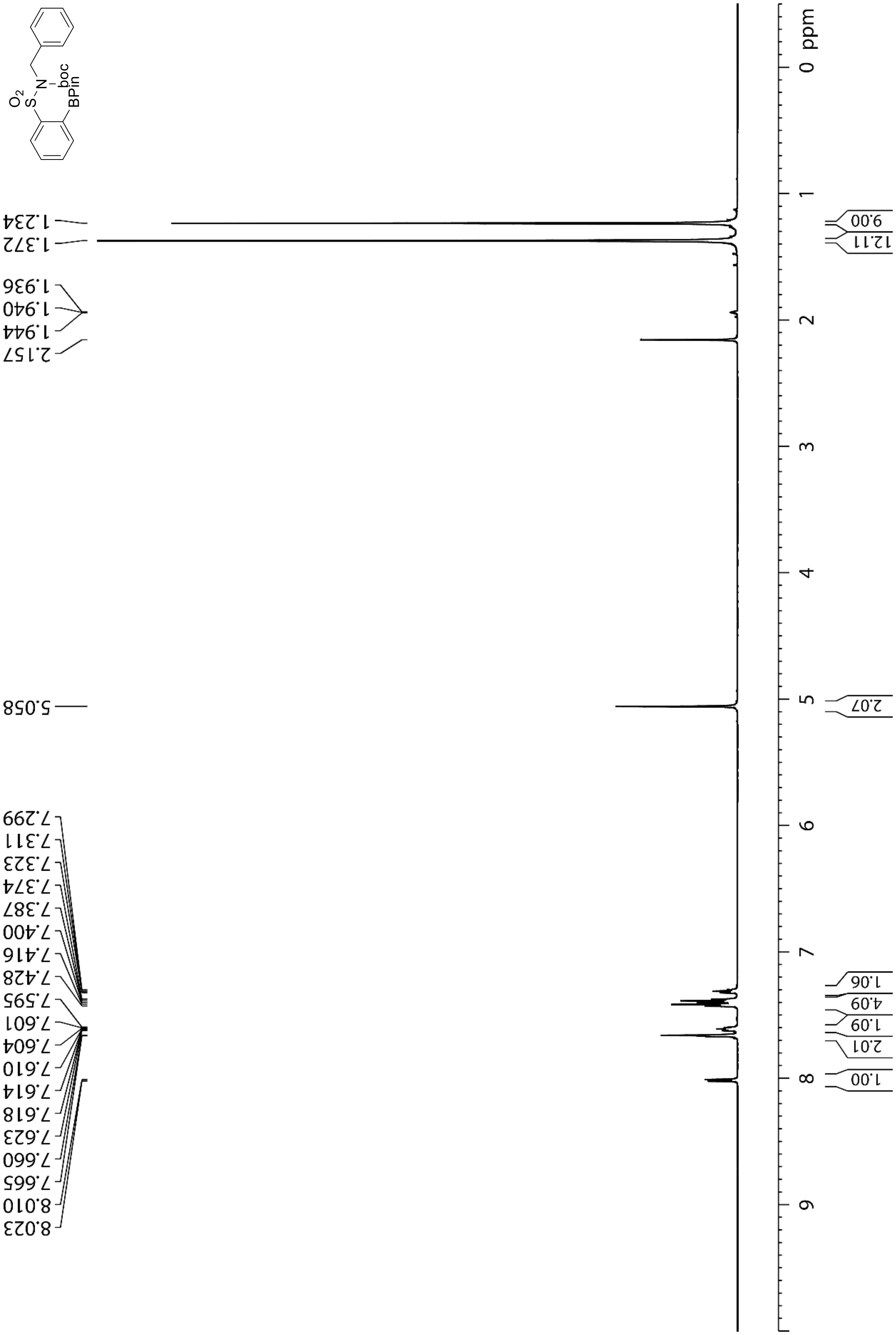

Figure S66. ^{1}H NMR spectrum of *tert*-butyl benzyl((2-(4,4,5,5-tetramethyl-1,3,2-dioxaborolan-2-yl)phenyl)sulfonyl)carbamate **S6** in CD_3CN

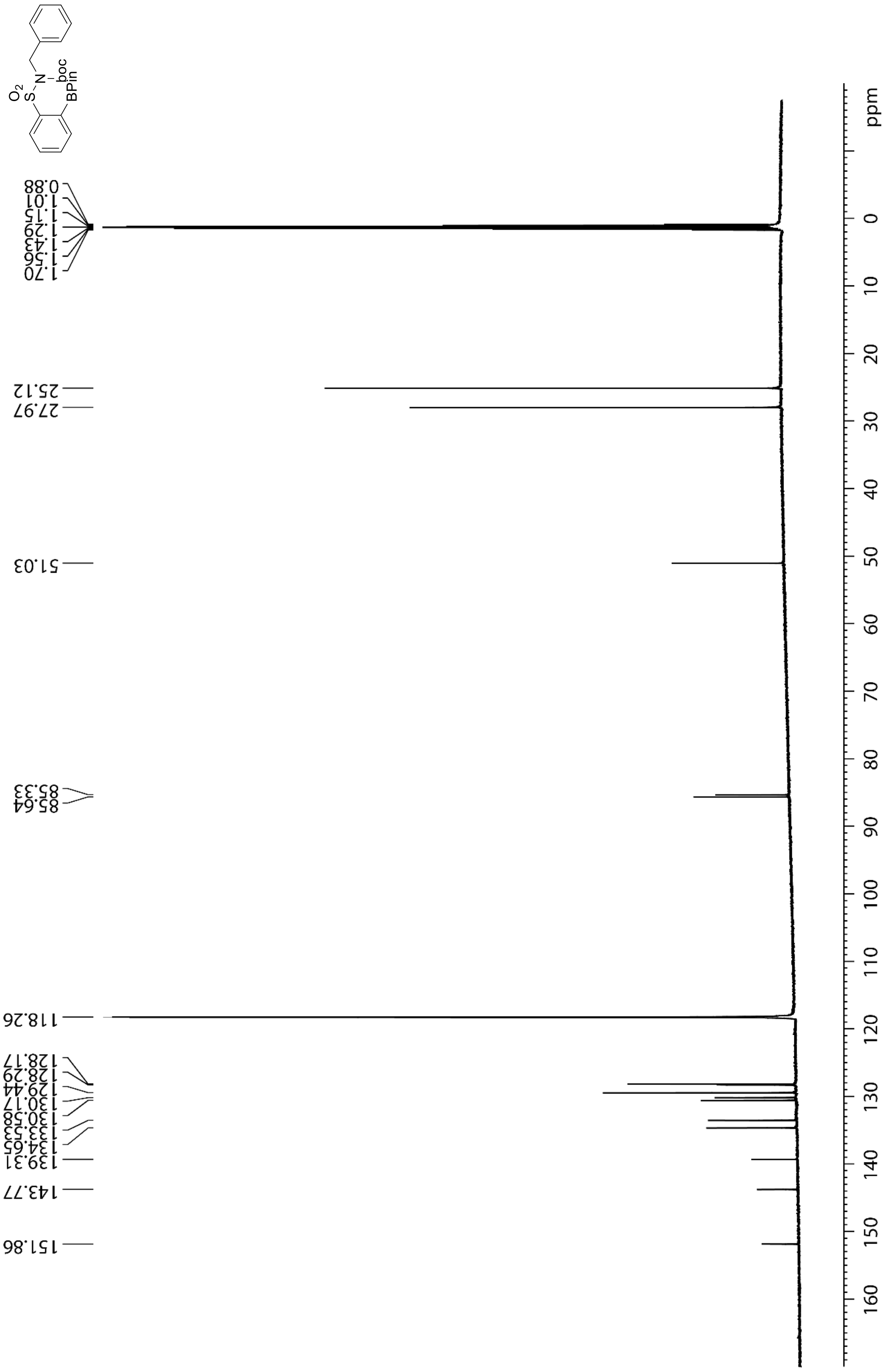

Figure S67. ^{13}C NMR spectrum of *tert*-butyl benzyl((2-(4,4,5,5-tetramethyl-1,3,2-dioxaborolan-2-yl)phenyl)sulfonyl)carbamate **S6** in CD_3CN

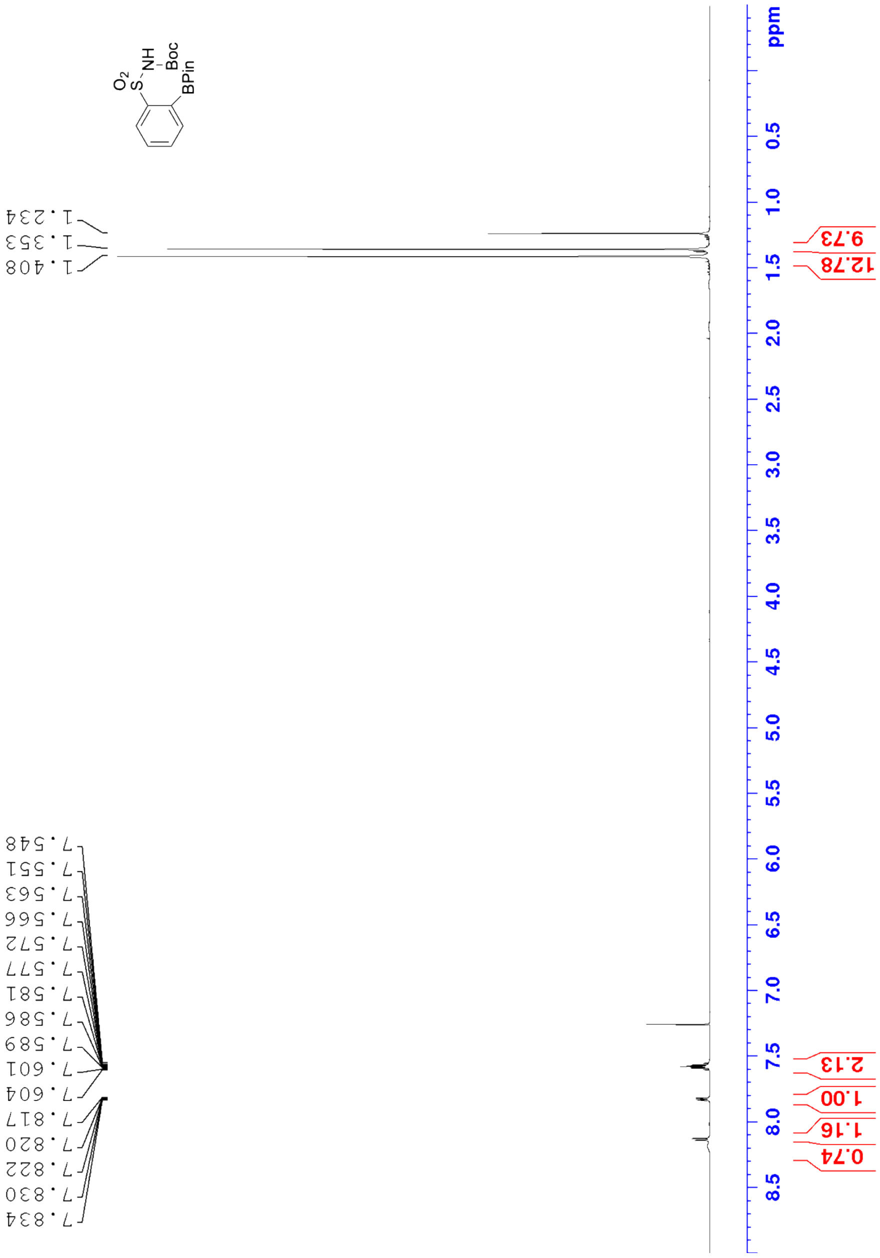

Figure S68. ^{1}H NMR spectrum of *tert*-butyl ((2-(4,4,5,5-tetramethyl-1,3,2-dioxaborolan-2-yl)phenyl)sulfonyl)carbamate **S7** in $CDCl_3$

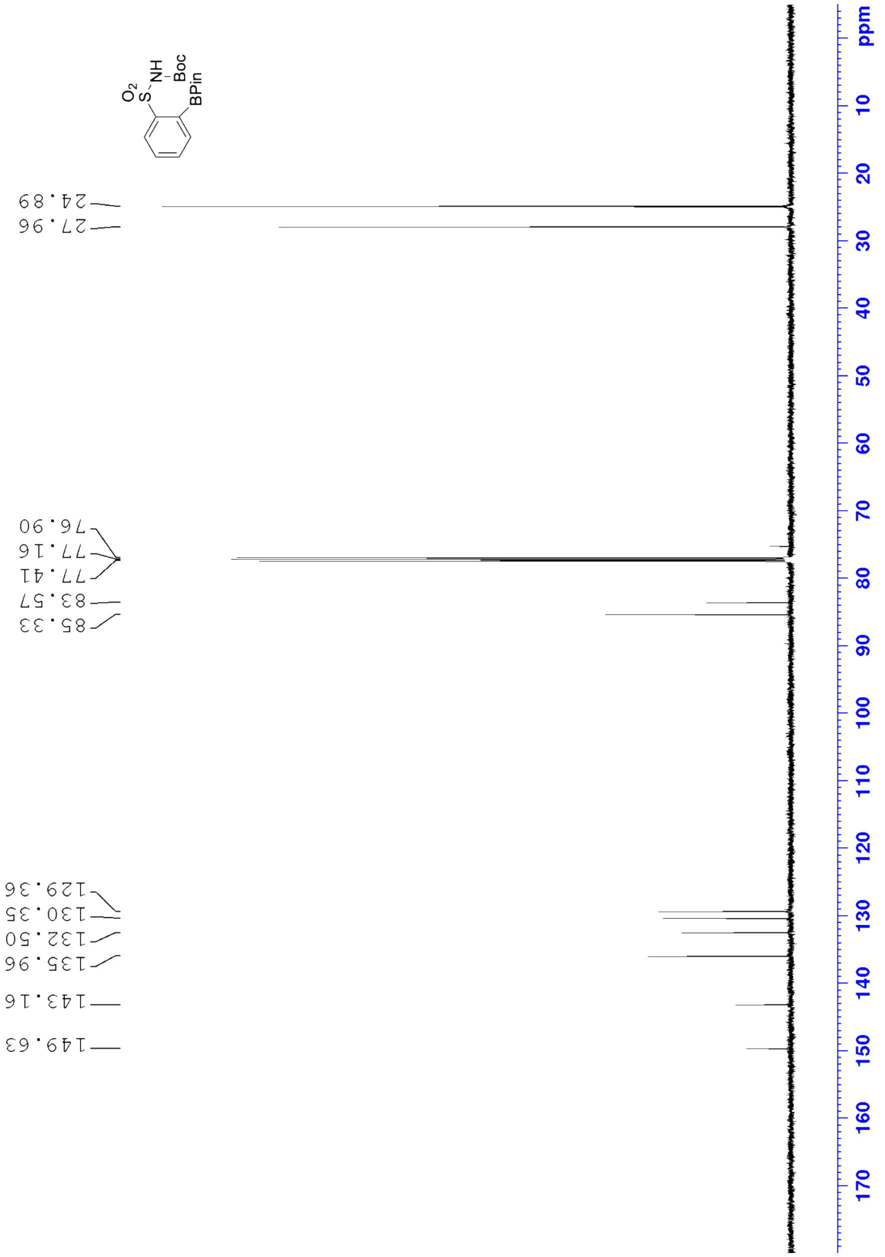

Figure S69. ^{13}C NMR spectrum of *tert*-butyl ((2-(4,4,5,5-tetramethyl-1,3,2-dioxaborolan-2-yl)phenyl)sulfonyl)carbamate **S7** in $CDCl_3$

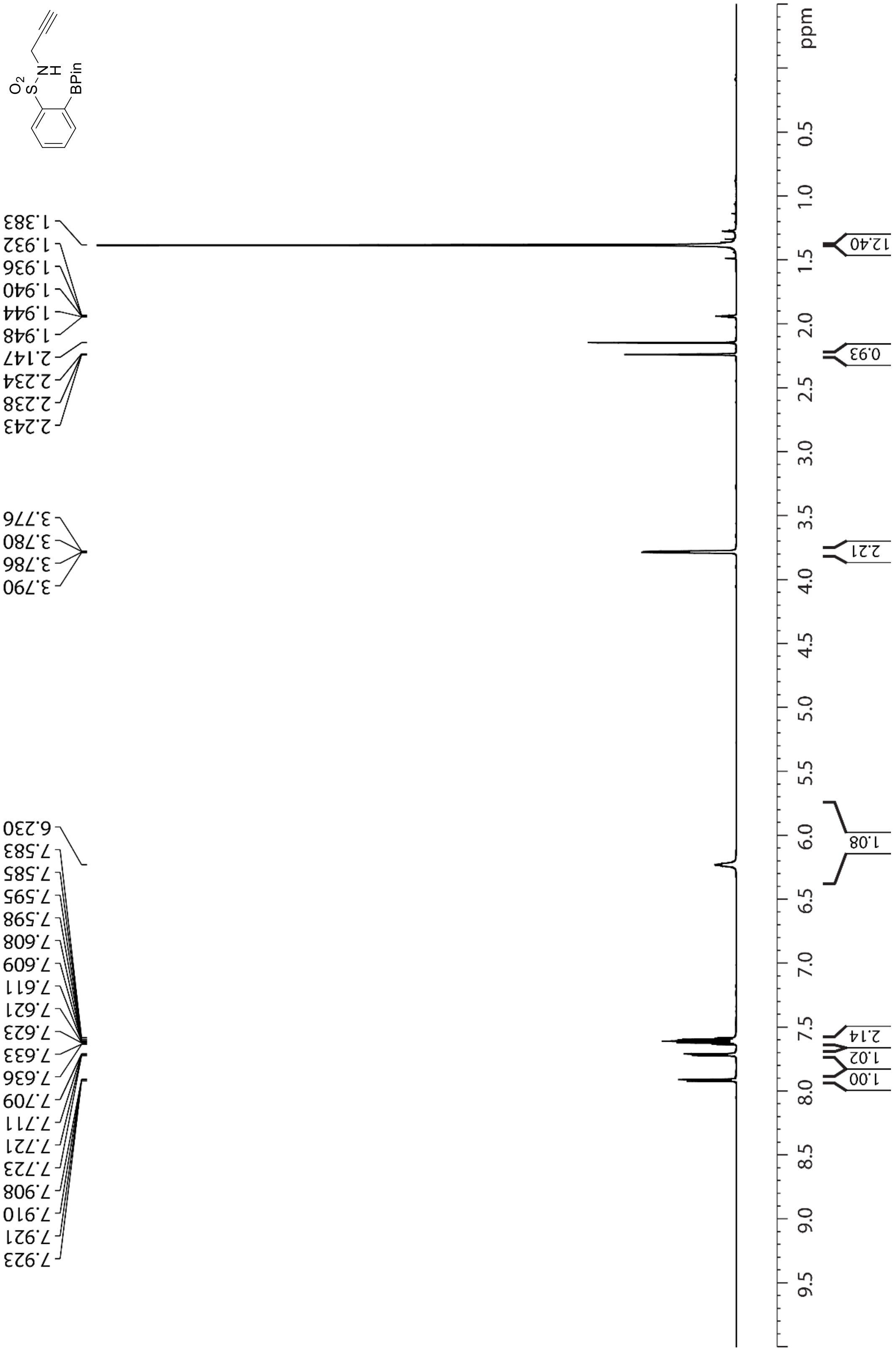

Figure S70. ^{1}H NMR spectrum of *N*-(prop-2-yn-1-yl)-2-(4,4,5,5-tetramethyl-1,3,2-dioxaborolan-2-yl)benzenesulfonamide **1o** in CD_3CN

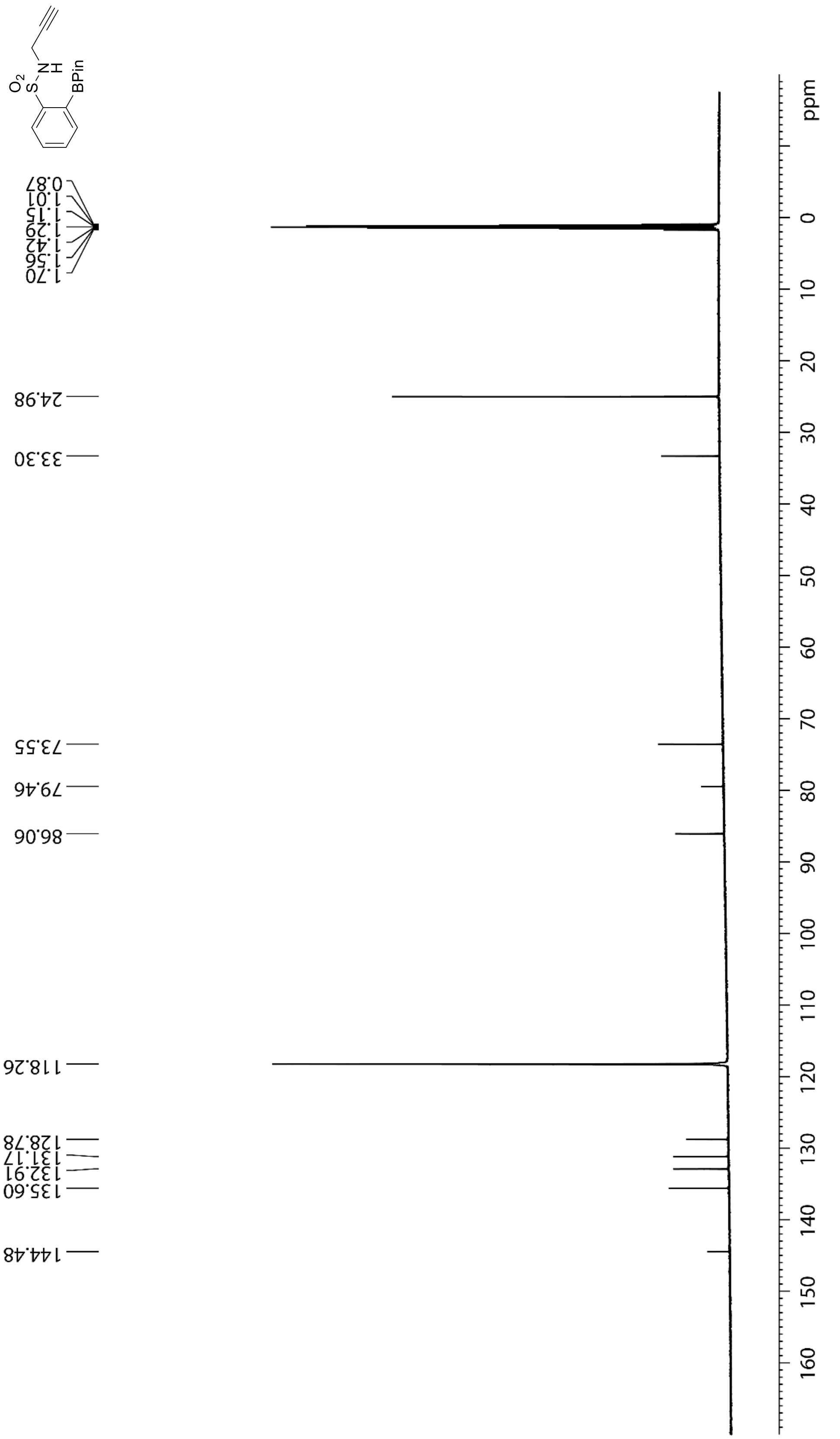

Figure S71. ^{13}C NMR spectrum of *N*-(prop-2-yn-1-yl)-2-(4,4,5,5-tetramethyl-1,3,2-dioxaborolan-2-yl)benzenesulfonamide **1o** in CD_3CN

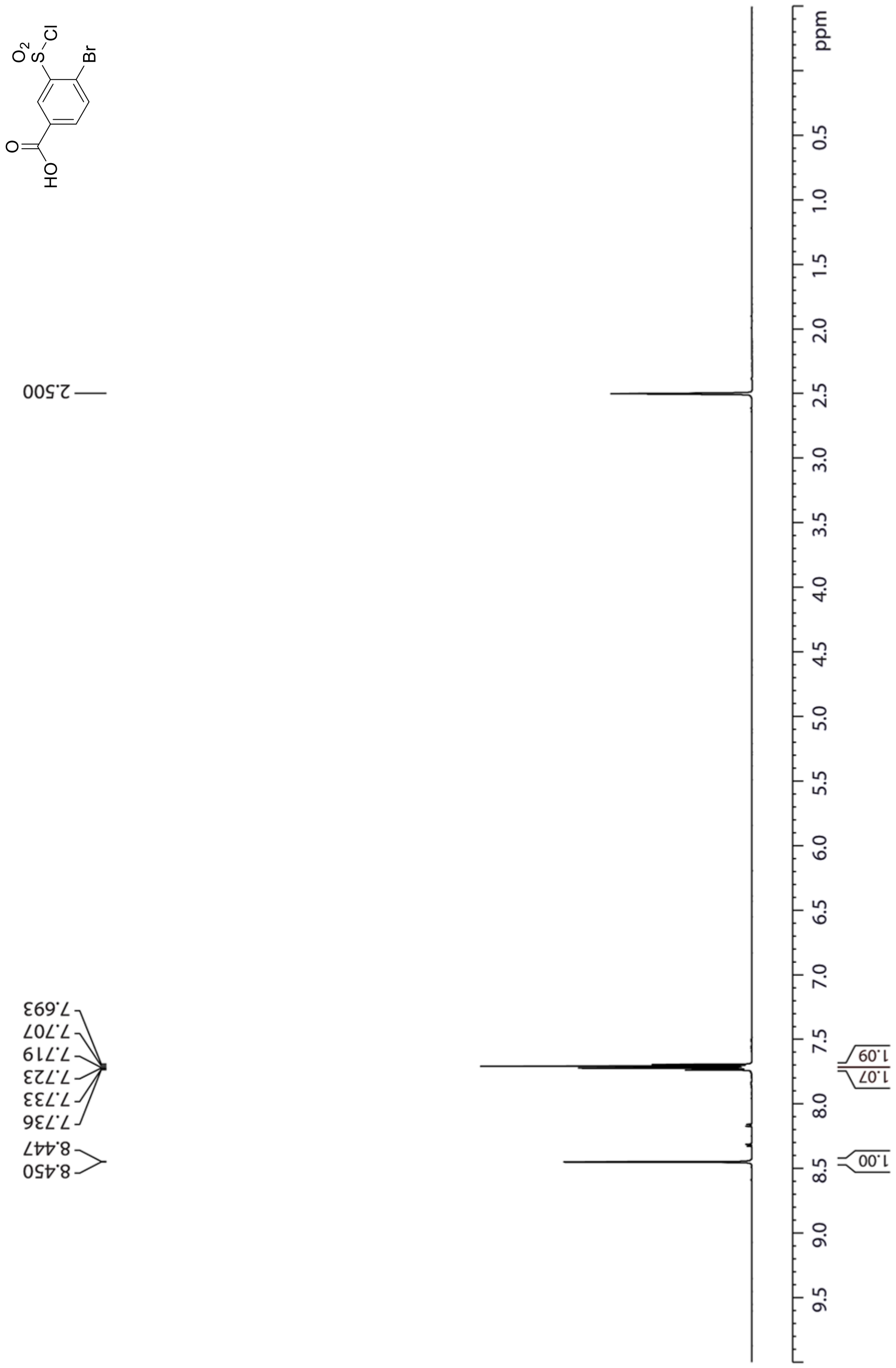

Figure S72. ^{1}H NMR spectrum of 4-bromo3-(chlorosulfonyl)benzoic acid **S8** in DMSO-d_6

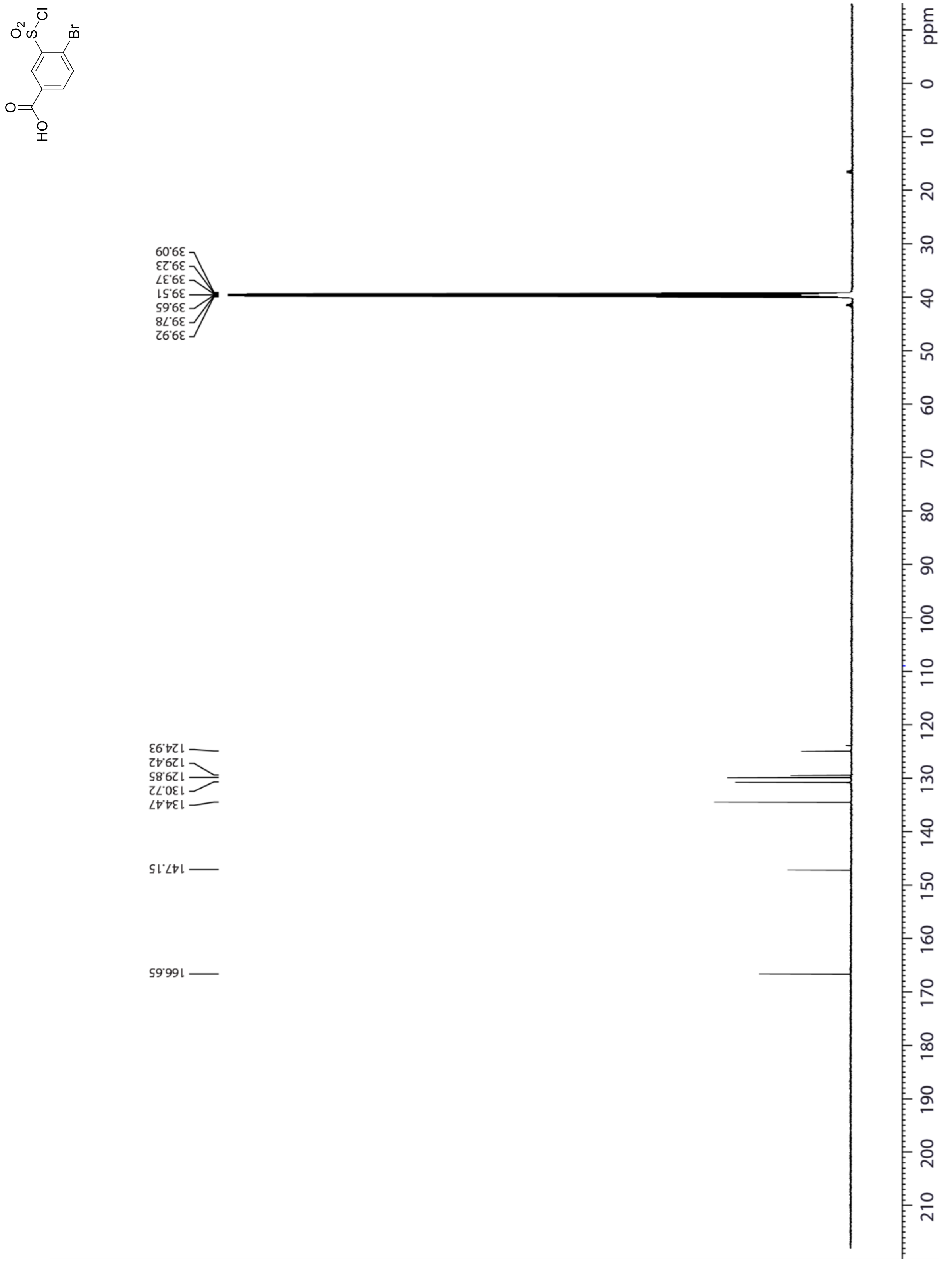

Figure S73. ^{13}C NMR spectrum of 4-bromo3-(chlorosulfonyl)benzoic acid **S8** in DMSO-d_6

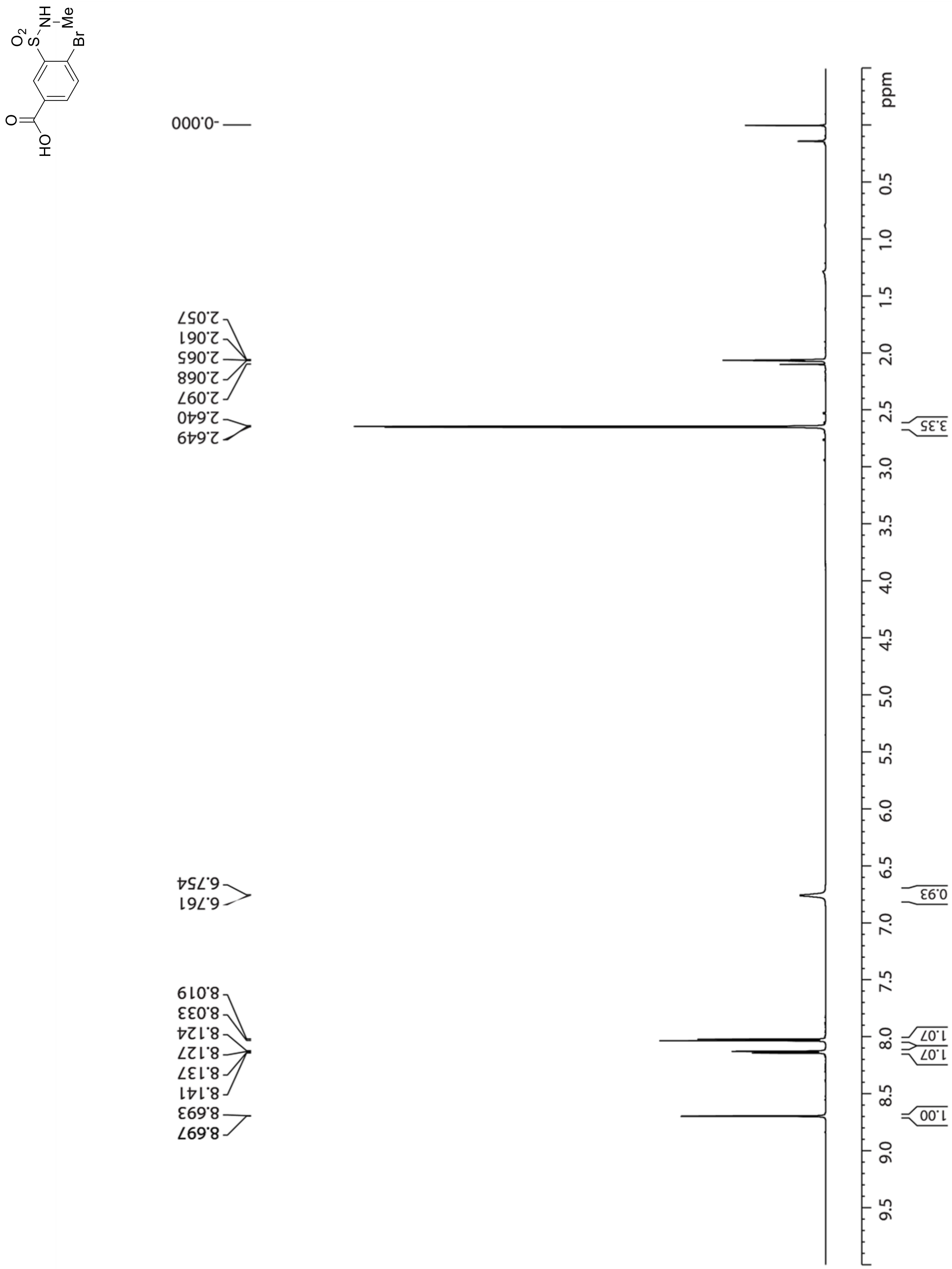

Figure S74. ^{1}H NMR spectrum of 4-bromo-3-(*N*-methylsulfamoyl)benzoic acid **S9** in acetone-d_6

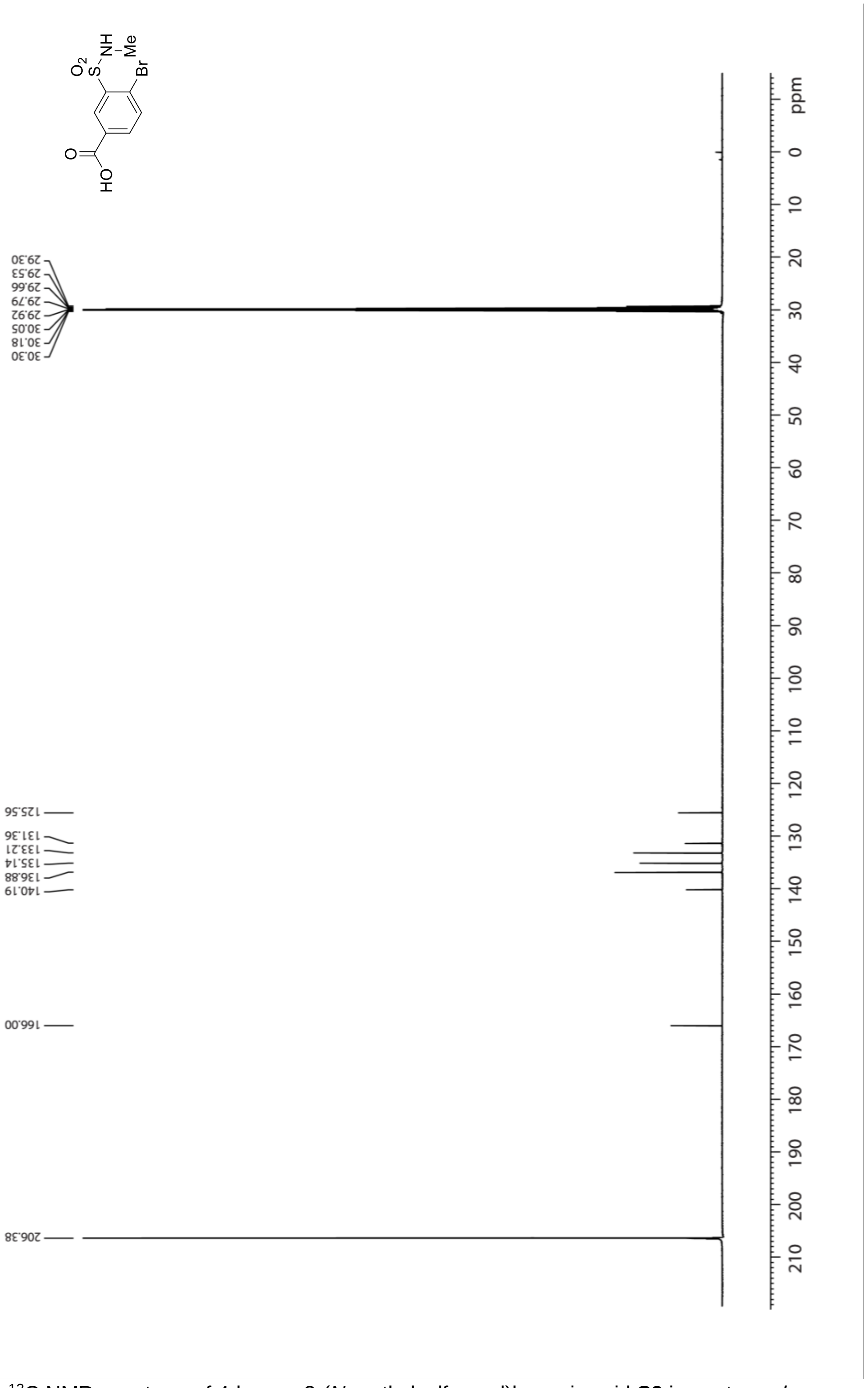

Figure S75. ^{13}C NMR spectrum of 4-bromo-3-(*N*-methylsulfamoyl)benzoic acid **S9** in acetone-d_6

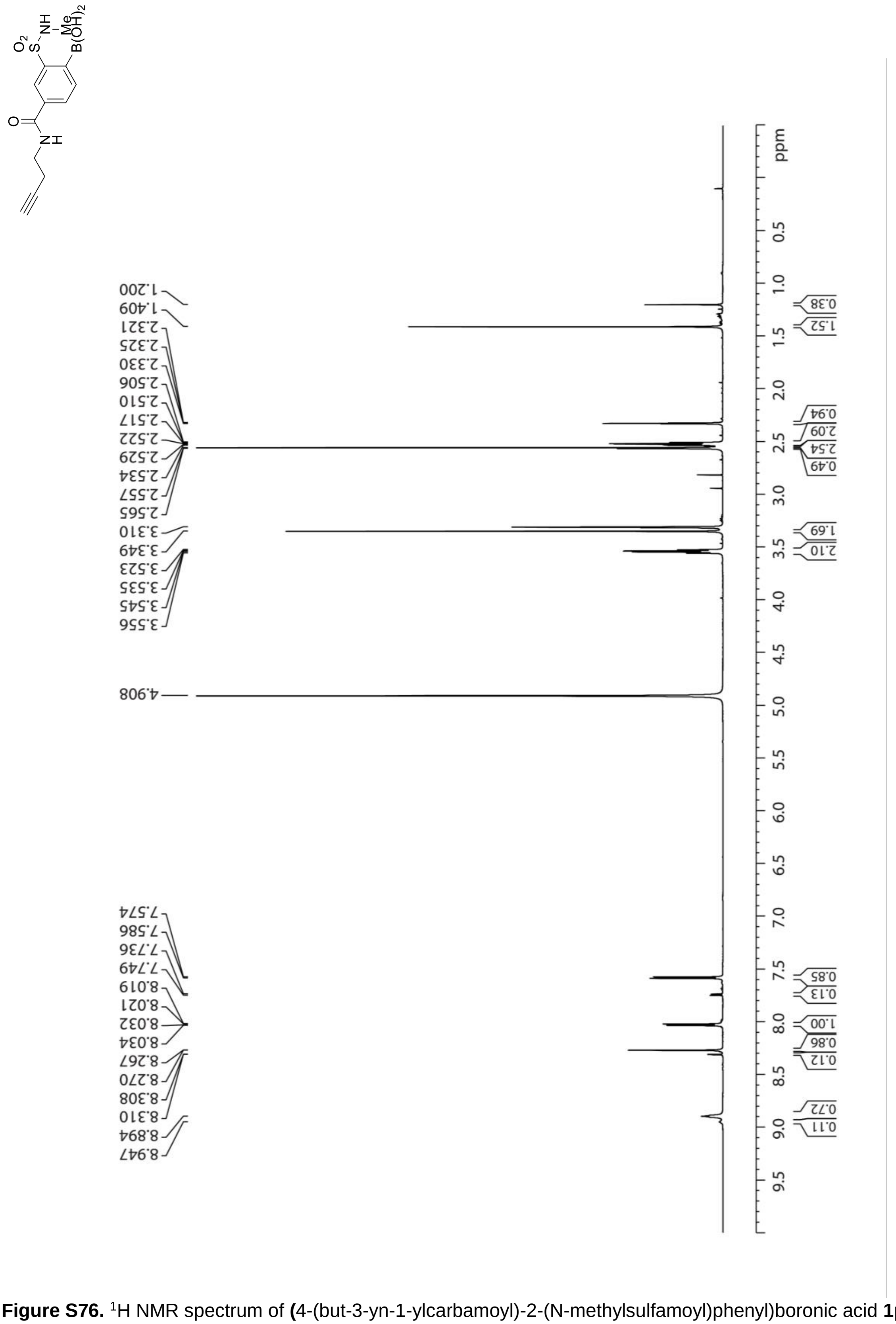

Figure S76. ^{1}H NMR spectrum of **(**4-(but-3-yn-1-ylcarbamoyl)-2-(N-methylsulfamoyl)phenyl)boronic acid **1p** in MeOD-d_4

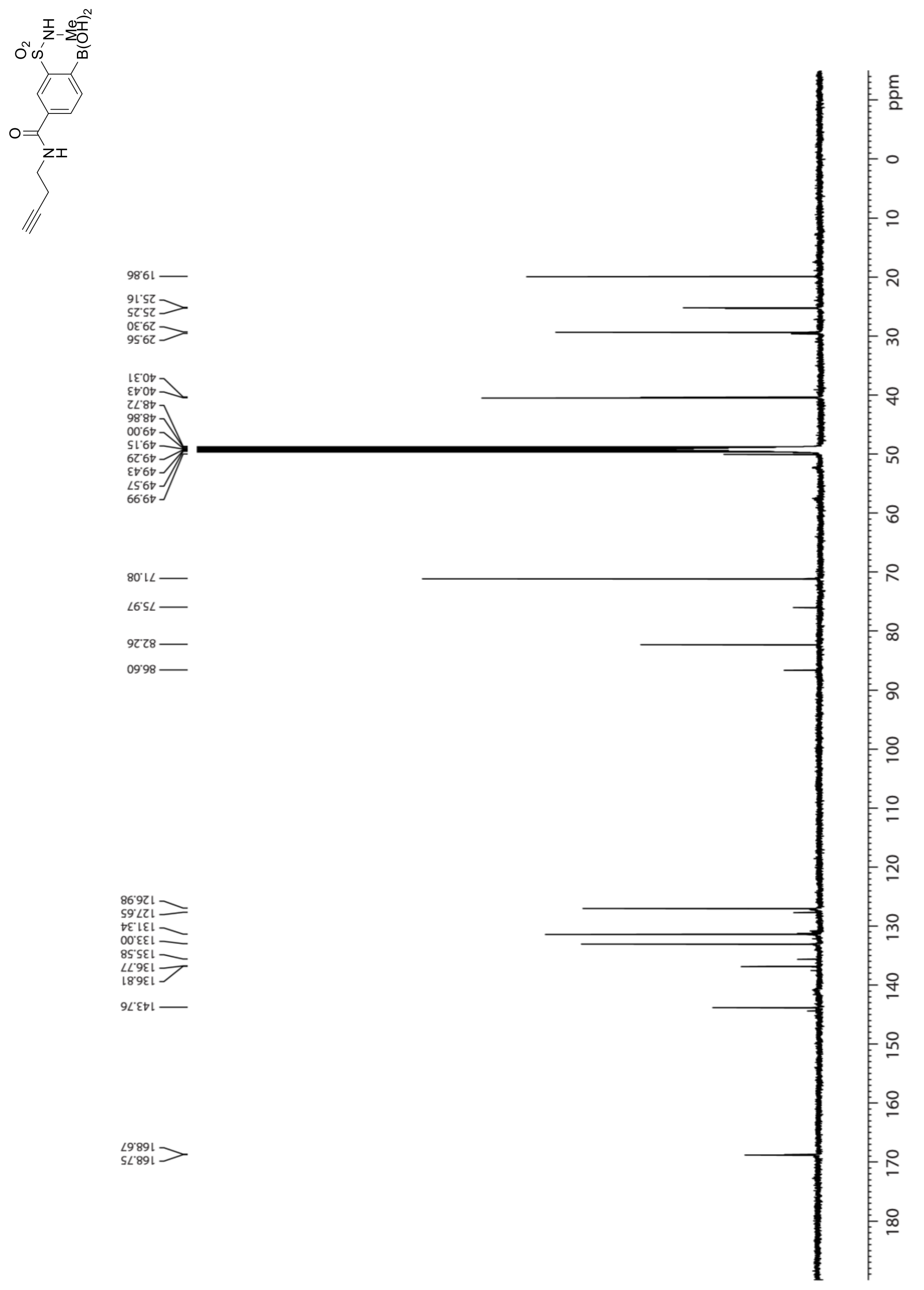

Figure S77. ^{13}C NMR spectrum of (4-(but-3-yn-1-ylcarbamoyl)-2-(N-methylsulfamoyl)phenyl)boronic acid 1p in MeOD-d_4

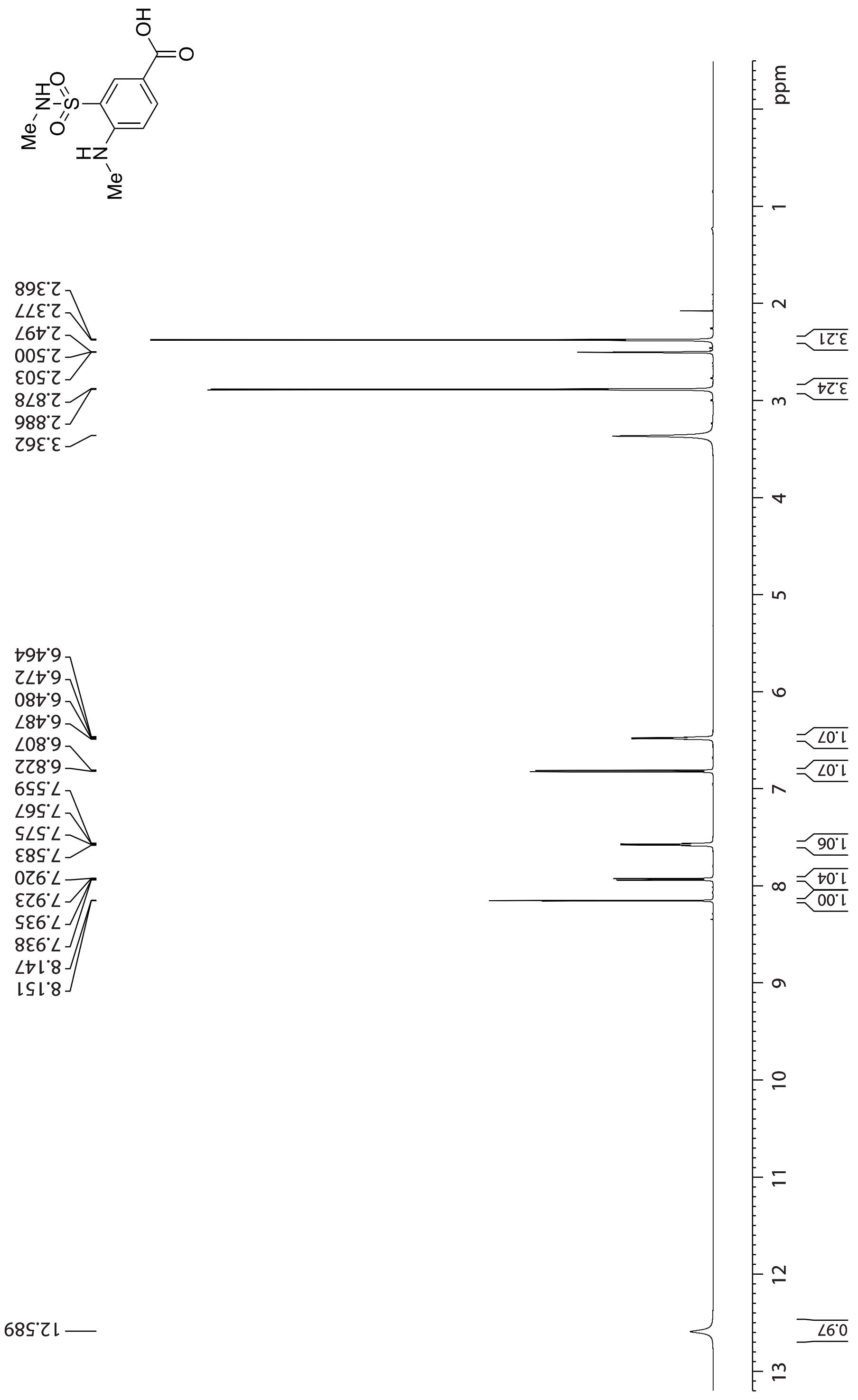

Figure S78. ¹H NMR spectrum of 4-(methylamino)-3-(N-methylsulfamoyl)benzoic acid **S11** in DMSO-d_6.

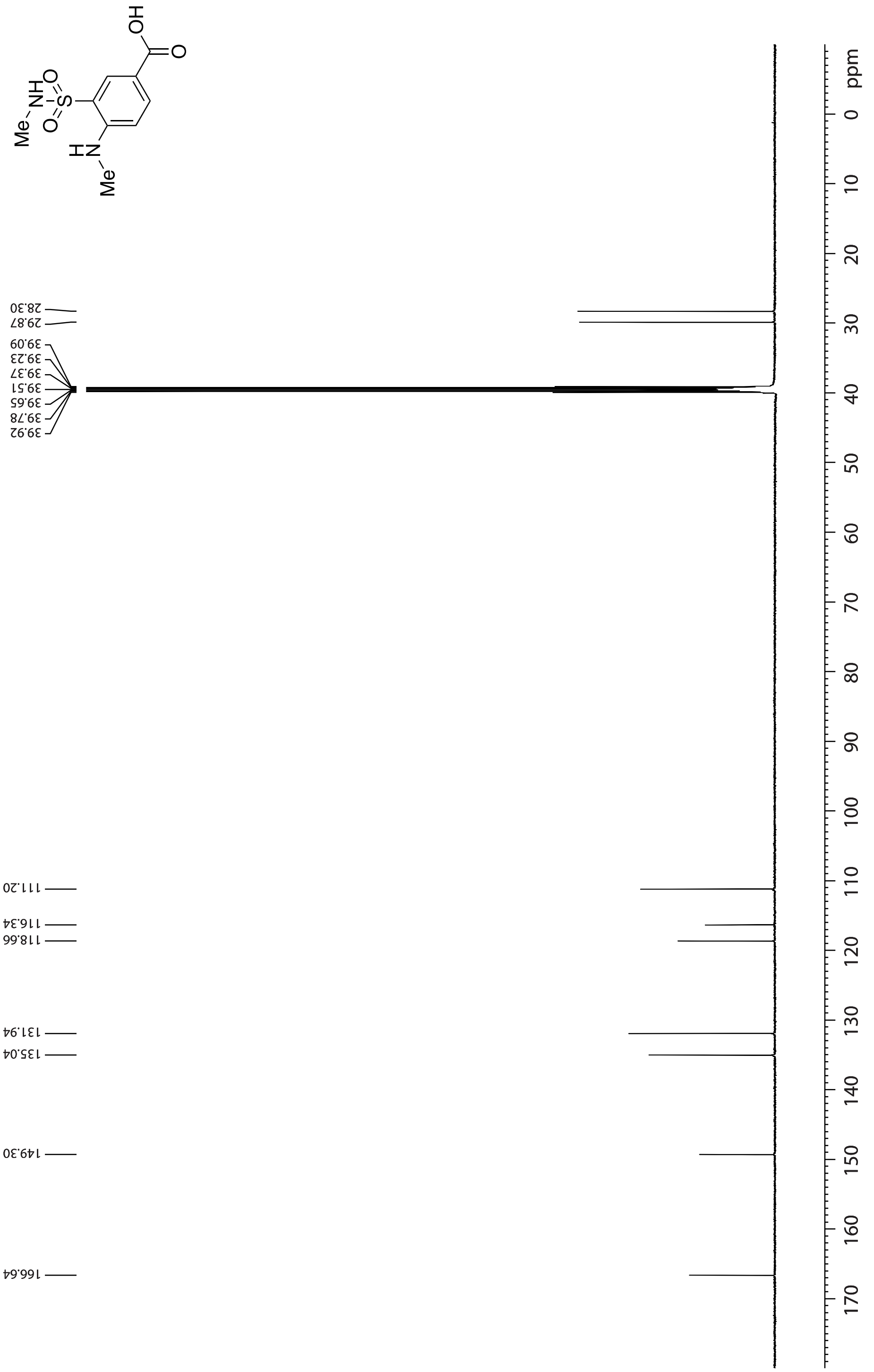

Figure S79. ^{13}C NMR spectrum of 4-(methylamino)-3-(N-methylsulfamoyl)benzoic acid **S11** in DMSO-d_6.

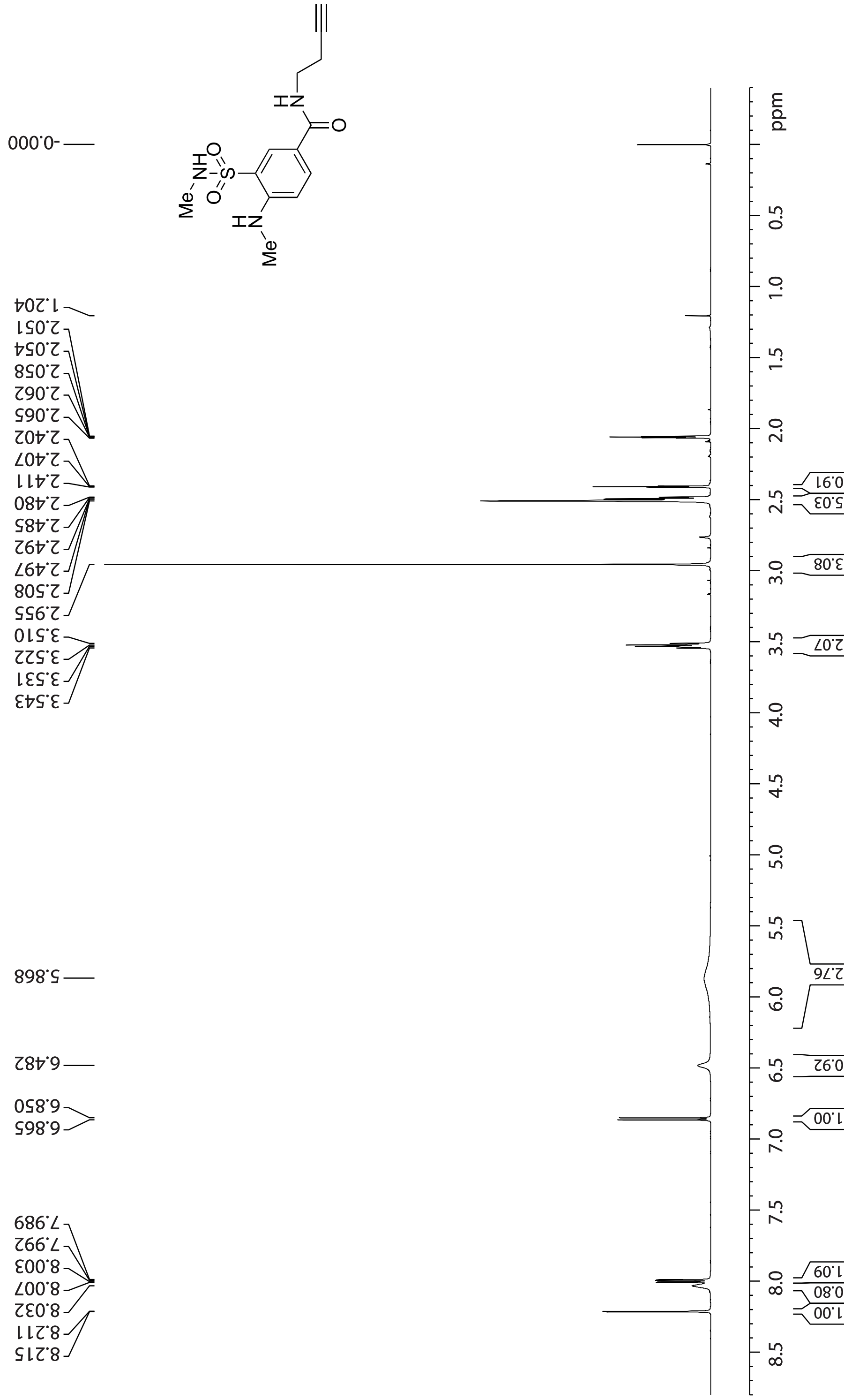

Figure S80. ^{1}H NMR spectrum of *N*-(but-3-yn-1-yl)-4-(methylamino)-3-(*N*-methylsulfamoyl)benzamide **S12** in Acetone-d_6.

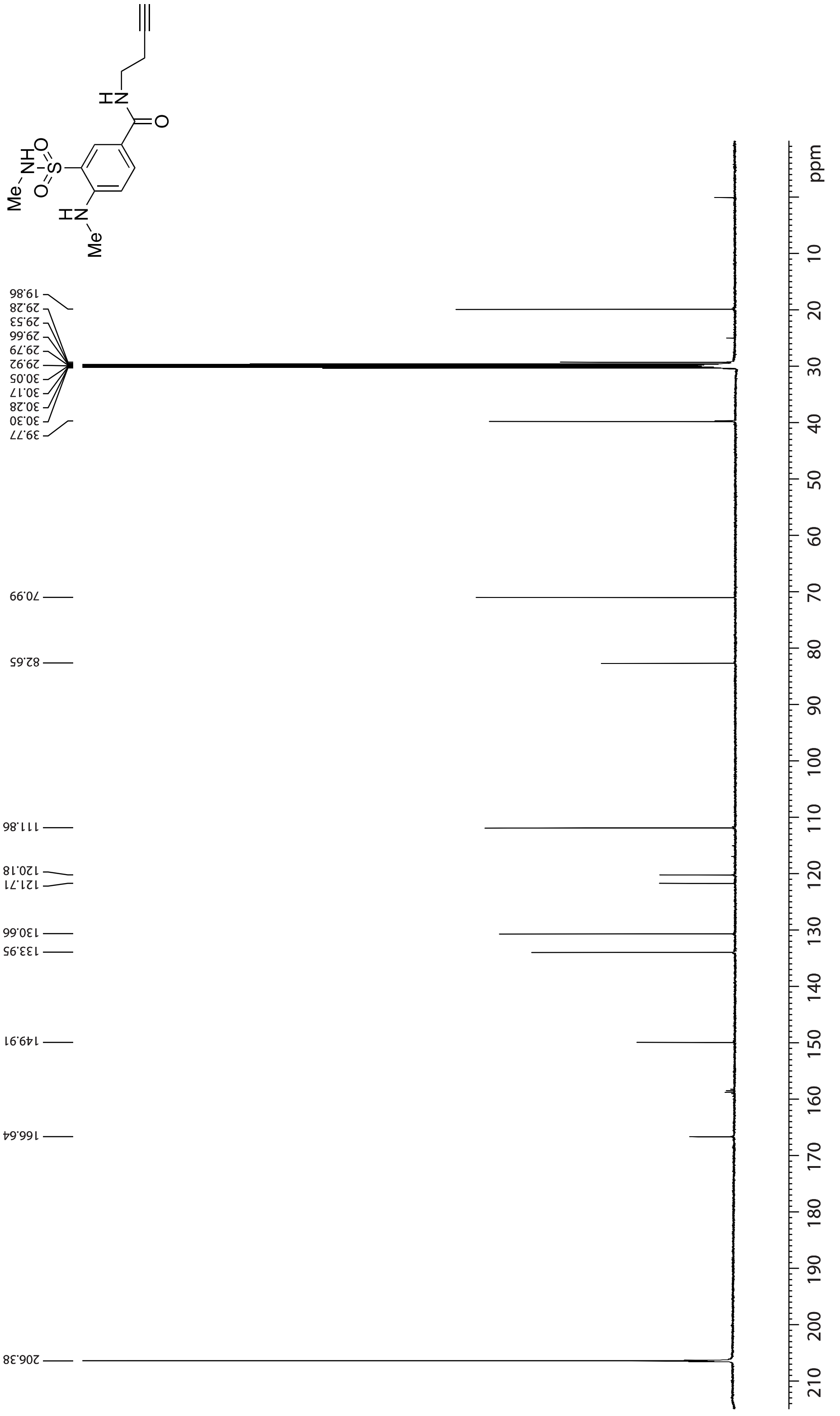

Figure S81. ^{13}C NMR spectrum of *N*-(but-3-yn-1-yl)-4-(methylamino)-3-(*N*-methylsulfamoyl)benzamide **S12** in Acetone-d_6.

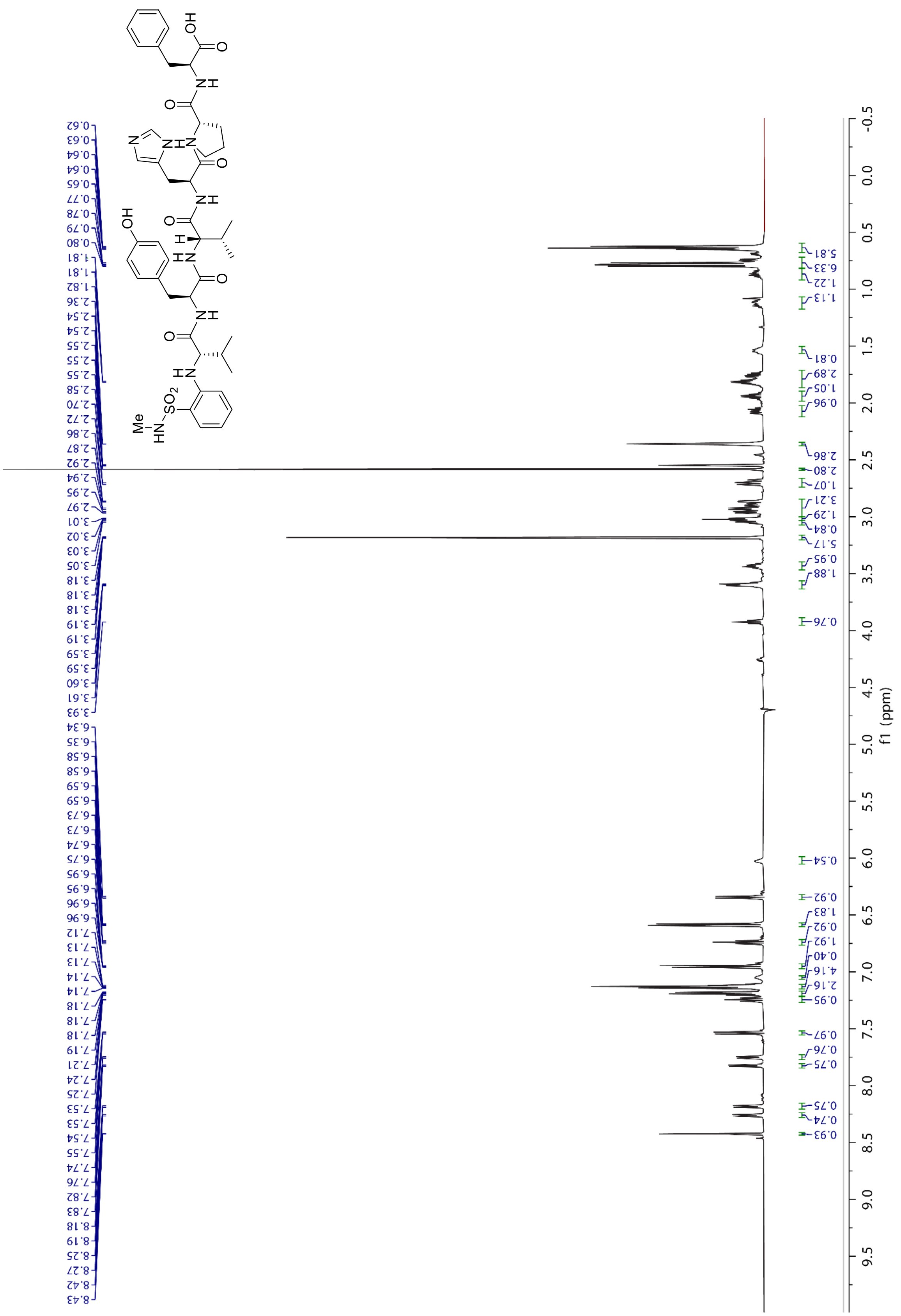

Figure S82. ^{1}H NMR spectrum of **5a** in 85:15 H_2O:D_2O.

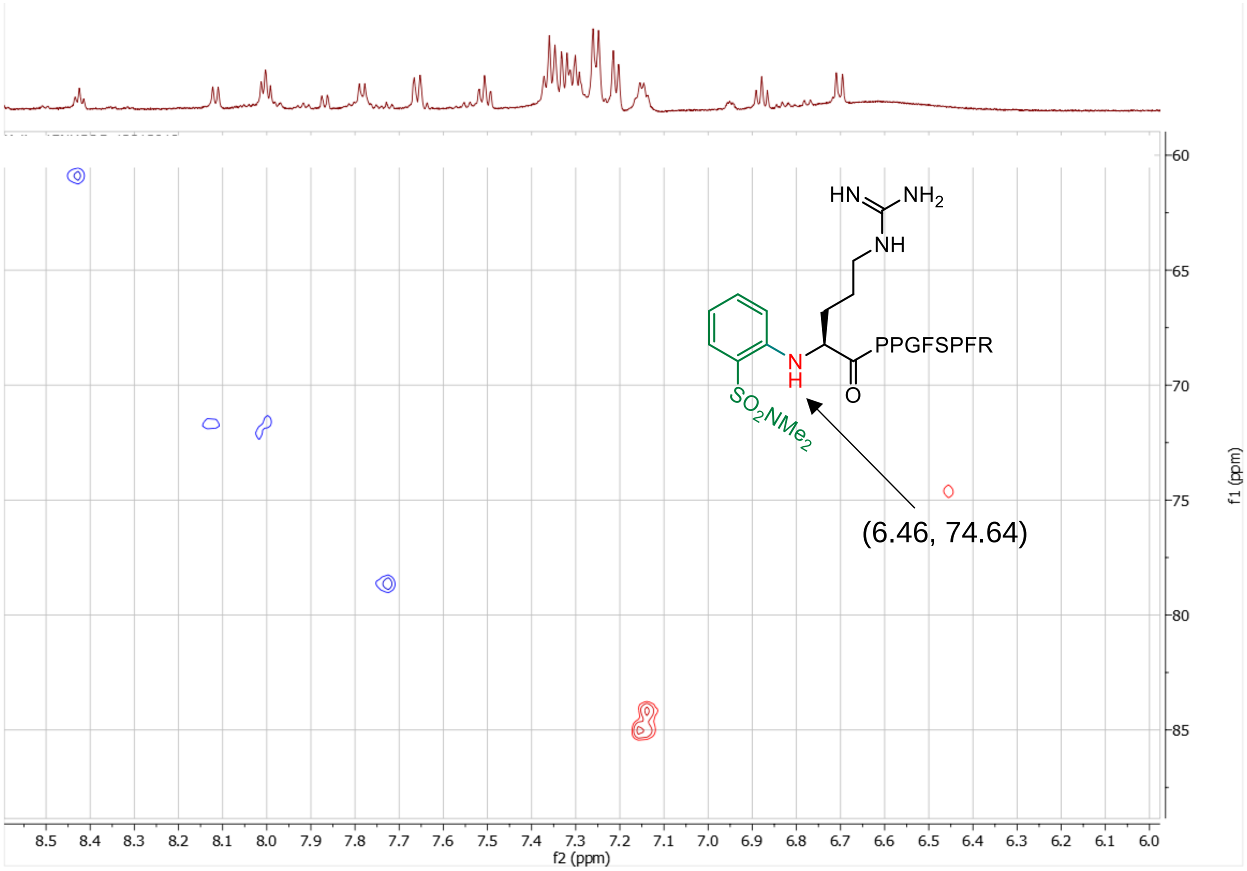

Figure S83. ^{15}N HSQC spectrum of **2a** in 85:15 H_2O:D_2O. Blue signals correspond to amide backbone protons, red signals at 7.15 ppm correspond to arginine Hε protons, and the labeled red signal at 6.46 ppm corresponds to the arylated N-terminus proton.

References

1. Wellings, D. A.; Atherton, E. “Methods in Enzymology Volume 289: Solid-Phase Peptide Synthesis” Ed. Fields, G. B. Academic Press: San Diego, 1997, p. 44-67.
2. Wu, F. Polycyclic Anaplastic Lymphoma Kinase Inhibitor. EP3202765, August 22, 2018.
3. Qiu, D.; He, J.; Yue, X.; Shi, J.; Li, Y. “Diamination of Domino Aryne Precursor with Sulfonamides.” *Org. Lett.* **2016**, *18*, 3130-3133.
4. Dolle, R., E.; Worm, K. SULFAMOYL BENZAMIDES AS CANNABINOID RECEPTOR MODULATORS. WO2007/58960, May 24, 2007.
5. Atli, T.; Craig, J., Ruble; Donna, L., Romero. ANTIBACTERIAL AGENTS. WO2004/18414, March 4, 2004.
6. Trice, Sarah Little Jane, "Palladium-Catalyzed Borylation And Cross-Coupling Of Aryl And Heteroaryl Halides Utilizing Dibora Derivatives" (2012).*Publicly Accessible Penn Dissertations*. 712. http://repository.upenn.edu/edissertations/712
7. Bao, X.; Rong, X.; Liu, Z.; Gu, Y.; Liang, G.; Xia, Q. Potassium Tert-Butoxide-Mediated Metal-Free Synthesis of Sulfonamides from Sodium Sulfinates and N,N-Disubstituted Formamides. *Tetrahedron Lett.* **2018**, *59* (29), 2853–2858. https://doi.org/10.1016/j.tetlet.2018.06.031.
8. Surry, D. S.; Fox, D. J.; Macdonald, S. J. F.; Spring, D. R. Aryl–Aryl Coupling via Directed Lithiation and Oxidation. *Chem. Commun.* **2005**, *0* (20), 2589–2590. https://doi.org/10.1039/B501939G.
